# Macromolecular Diamidobenzimidazole Conjugates Can Activate Stimulator of Interferon Genes

**DOI:** 10.1021/jacs.5c13195

**Published:** 2025-09-12

**Authors:** Karan Arora, Taylor L. Sheehy, Jacob A. Schulman, Jack R. Loken, Zachary Lehmann, Blaise R. Kimmel, Caitlin McAtee, Vijaya Bharti, Payton T. Stone, Alissa M. Weaver, Matthew Tyska, Rakesh Kumar Pathak, John T. Wilson

**Affiliations:** † Department of Chemical and Biomolecular Engineering, 5718 Vanderbilt University, Nashville, Tennessee 37212, United States; ‡ Department of Biomedical Engineering, Vanderbilt University, Nashville, Tennessee 37240, United States; § Interdisciplinary Materials Science Program, 5718 Vanderbilt University, Nashville, Tennessee 37240, United States; ∥ Department of Cell and Developmental Biology, 12328 Vanderbilt University, Nashville, Tennessee 37240, United States; ⊥ Department of Chemical Sciences, Indian Institutes of Science Education and Research (IISER Berhampur), Berhampur, Odisha 760010, India; # Vanderbilt Institute of Chemical Biology, Vanderbilt University, Nashville, Tennessee 37240, United States; ∇ Vanderbilt Institute of Nanoscale Science and Engineering, Vanderbilt University, Nashville, Tennessee 37212, United States; ○ Vanderbilt Institute for Infection, Immunology, and Inflammation, Vanderbilt University, Nashville, Tennessee 37232, United States; ◆ Vanderbilt Center for Immunobiology, Vanderbilt University Medical Center, Nashville, Tennessee 37232, United States; ¶ Vanderbilt Ingram Cancer Center, Nashville, Tennessee 37232, United States

## Abstract

A promising class of stimulator of interferon genes (STING) agonists is the non-nucleotide, small molecule, dimeric amidobenzimidazoles (diABZI), which have recently been incorporated into polymer- and antibody-drug conjugates to improve pharmacokinetics and modulate biodistribution for disease-specific applications. These approaches have leveraged diABZI variants functionalized at the 7-position of the benzimidazole for conjugation and tunable drug release from carriers. However, since this position does not interact with STING and is exposed from the binding pocket when bound in an “open lid” configuration, we sought to evaluate the activity of macromolecular diABZI conjugates that lack stimuli-responsive release and are instead conjugated to polymers via a stable amide linker. By synthesizing stable mPEG-diABZI conjugates and *N*,*N*-dimethylacrylamide (DMA) homopolymers from a diABZI-functionalized reversible addition–fragmentation chain-transfer (RAFT) agent, we found that these conjugates could activate STING *in vitro* with similar kinetics to highly potent diABZI analogues. Our data indicate that although diABZI-DMA conjugates enter cells via endocytosis, they can still colocalize with the ER, suggesting that intracellular trafficking processes can promote the delivery of endocytosed macromolecular diABZI compounds to STING. Furthermore, we demonstrated the *in vivo* activity of these macromolecular diABZI platforms, which inhibited tumor growth to a similar extent as small molecule variants. In conclusion, we have described new chemical strategies for the synthesis of stable macromolecular diABZI conjugates with unexpected immunostimulatory activity—findings that have potential implications for the design of polymer-diABZI conjugates and further motivate investigation of endosomal and intracellular trafficking as an alternative route for STING activation.

## Graphical abstract

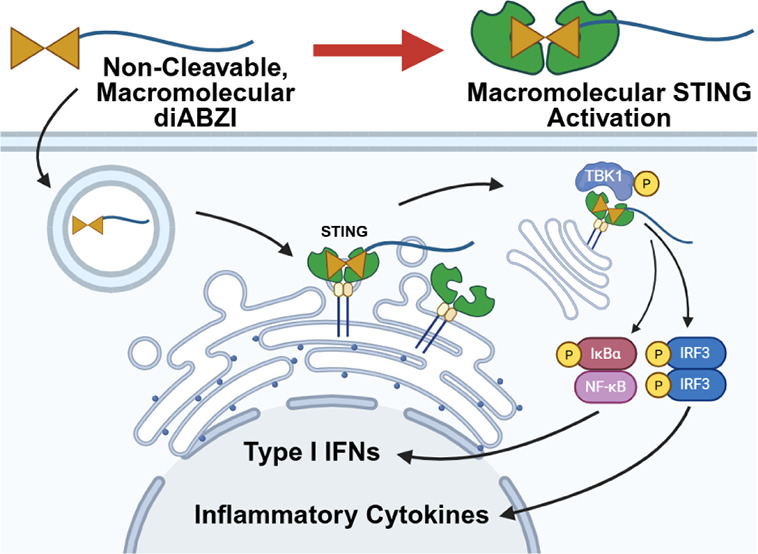

## Introduction

The stimulator of interferon genes (STING) pathway is an ancient and evolutionarily conserved innate immune sensing mechanism with critical roles in pathogen detection, tumor immune surveillance, and maintenance of tissue homeostasis.^1^ STING is a transmembrane protein predominantly localized on the endoplasmic reticulum (ER) and is activated upon the binding of several cyclic dinucleotides (CDNs), including 2′3′-cGAMP, the endogenous STING ligand that is synthesized by the enzyme cyclic GMP-AMP synthase (cGAS) in response to the detection of cytosolic double-stranded DNA.^2,3^ Activation of STING triggers the secretion of type-I interferons (IFN-Is) and other proinflammatory cytokines that promote immune responses against viruses, bacteria, and cancers.^4,5^ Accordingly, STING agonists are an emerging and promising class of therapeutics with broad applications as antiviral agents, cancer immunotherapies, and vaccine adjuvants.^6,7^

Although CDNs have been widely explored for diverse applications and continue to be optimized for clinical translation, their activity and efficacy are typically limited due to poor drug-like properties that result in rapid clearance, poor cellular uptake, and limited access to the cytosol for STING binding.^8,9^ To address this, several non-CDN small molecule STING agonists have been developed with improved membrane permeability and favorable properties for systemic administration.^10–12^ One promising class of small molecule STING agonists is dimeric amidobenzimidazoles (diABZI), which were first described by Ramanjulu et al. and are currently under clinical investigation for immuno-oncology (e.g., NCT03843359).^10^ While relatively large by conventional small molecule standards (e.g., >700 Da), diABZI compounds can diffuse across cell membranes, allowing for cytosolic access and binding to STING.^10^ Hence, diABZI compounds have increased potency compared to CDNs and pharmacological properties that confer improved therapeutic efficacy in mouse tumor models when administered systemically.^10,13^ However, the reported serum half-life of diABZI is relatively short (i.e., ∼90 min),^10^ and membrane permeability and lack of cell or organ (e.g., tumor) tropism result in indiscriminate STING activation with the potential for inflammatory toxicities (e.g., cytokine release syndrome).

Akin to other classes of therapeutics (e.g., chemotherapy drugs), the development of carrier-drug conjugates has the potential to overcome these pharmacological barriers.^14,15^ Indeed, we and others have recently described the development of polymer- and protein-drug conjugates for improved delivery of diABZI compounds.^16–20^ Central to this approach is the synthesis of diABZI variants with reactive handles installed at the 7-position of one of the benzimidazole groups, which does not interact with STING and is exposed from the binding pocket when bound in an “open lid” configuration (Figure 1B).^10,21^ In contrast, the binding of CDNs and synthetic, non-nucleotide agonists (e.g., MSA-2) to STING results in a “closed lid” conformation that wraps around the agonist and effectively buries it within the protein (Figure 1A).^22^ A second important consideration is the choice of linker chemistry, which has the potential to allow for diABZI release from carriers under specific intracellular or microenvironmental conditions. To date, however, only cathepsin-cleavable linkers for intracellular release have been utilized. While it is logical to integrate cleavable linkers that allow potent diABZI analogues to be released from macromolecular carriers, because diABZI binds to STING in an “open lid” configuration, it is also conceivable that diABZI compounds covalently linked to bulky groups or even macromolecules could still bind to and activate STING. This possibility is supported by evidence demonstrating that conjugation of diABZI to small molecules (<1000 Da), such as fluorescent dyes and PET probes, can bind to and activate STING when used for *in vivo* imaging of STING expression.^23^ Notably, a recent study demonstrated that conjugation of diABZI to peptide antigens can target the peptide to the ER membrane via STING binding, resulting in increased MHC-I antigen presentation with concurrent STING activation.^24,25^ Hence, there is mounting evidence that covalently modified diABZI analogues can access and bind to STING on the ER membrane. However, while these modifications are likely to dramatically impair membrane permeability, the resultant conjugates are still relatively small (∼1500–2500 Da), and it remains unknown if larger macromolecules can also be conjugated to diABZI while still allowing for STING binding and activation.

**Figure 1:**
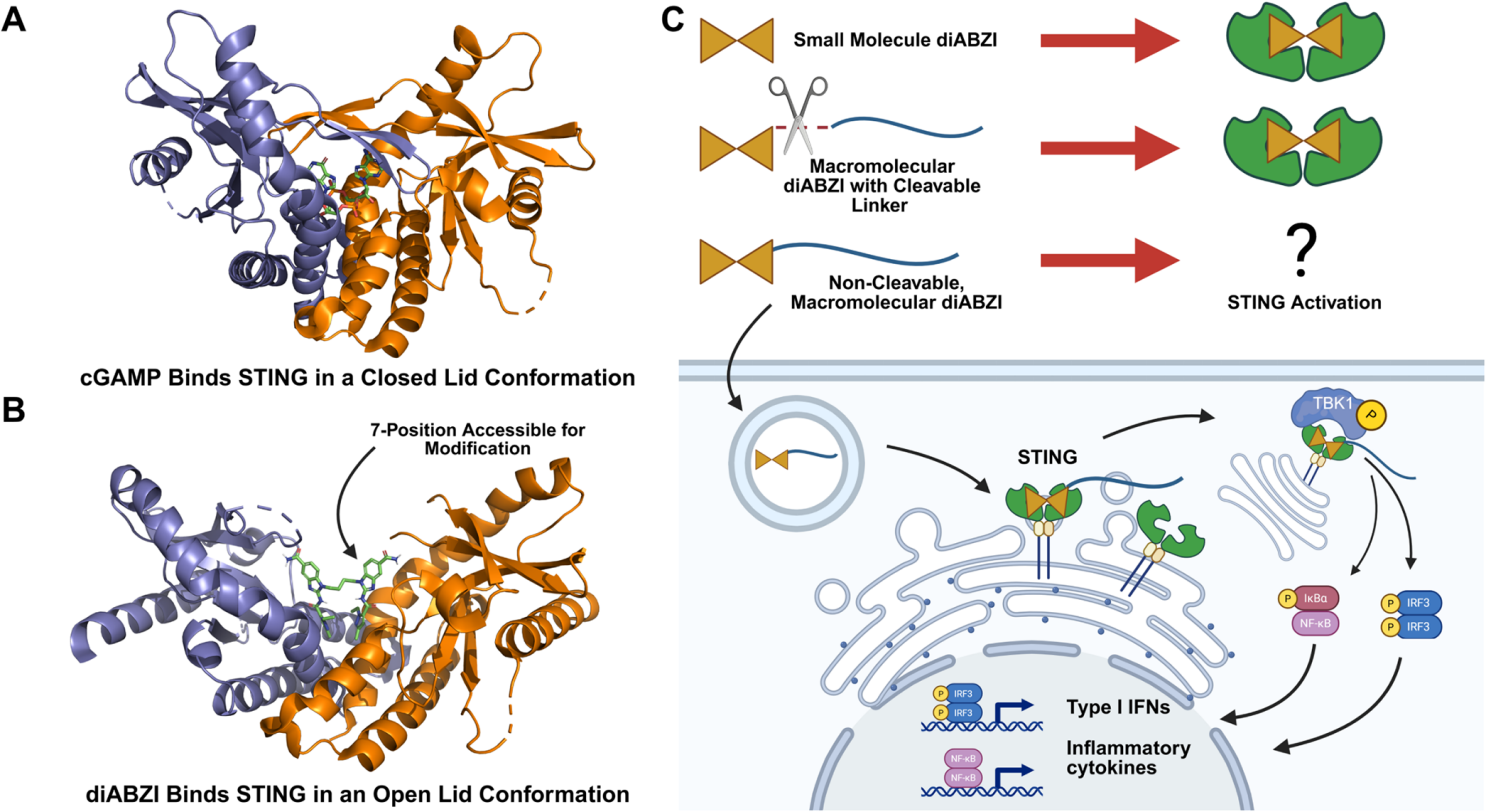
Dimeric amidobenzimidazole (diABZI) compounds conjugated to hydrophilic macromolecules have the potential to bind and activate STING. (A) Crystal structure of cGAMP binding to STING demonstrates binding in a “closed lid” conformation (PDB 4KSY). (B) Crystal structure of diABZI binding to STING demonstrates binding in an “open lid” conformation (PDB 6DXL).^10,21^ The 7-position of the benzimidazole extends from the binding pocket, providing a potential site for covalent ligation of diABZI to large macromolecules while still allowing for STING binding and activation. (C) Schematic illustrating that small molecule diABZI compounds and diABZI linked to polymeric carriers via cleavable linkers can activate STING, with the possibility that macromolecular diABZI conjugates can access, bind, and activate STING to stimulate type-I interferons (IFN-Is) and proinflammatory cytokines. Images were created using BioRender.

Toward addressing this knowledge gap, we asked whether diABZI molecules conjugated to water-soluble polymer chains via stable amide linkages could still activate STING signaling (Figure 1C). To do so, we synthesized stable mPEG-diABZI conjugates and large *N*,*N*-dimethylacrylamide (DMA) homopolymers using a diABZI-functionalized reversible addition–fragmentation chain-transfer (RAFT) agent. Surprisingly, we found that these conjugates could activate STING *in vitro* with kinetics similar to those of highly potent diABZI analogues. Additionally, these polymer-diABZI conjugates were able to activate STING *in vivo*, leading to the inhibition of tumor growth in mouse models. Through mechanisms that remain to be fully elucidated, we observed that polymer-diABZI conjugates enter cells via endocytosis but can colocalize with the ER, where STING is typically activated. These data suggest that diABZI covalently linked to large, hydrophilic, macromolecular carriers may access, bind, and activate STING, potentially mediated by vesicular transport processes that allow endocytosed polymer-diABZI conjugates to be trafficked to the ER. Therefore, our findings have important implications for the design of polymer-diABZI conjugates and may offer insight into how endocytic and intracellular trafficking can be harnessed to target diABZI to the ER for activation of STING.

## Results

### Synthesis and Characterization of diABZI-PEG Conjugates

We first sought to determine whether the covalent linkage of diABZI to a bulky, hydrophilic macromolecular carrier through a stable amide bond would allow for STING binding and *in vitro* activation. As a model polymeric carrier, we selected linear poly­(ethylene glycol) (PEG) based on its common use as a biocompatible carrier for enhancing aqueous solubility and improving circulation half-life.^26,27^ To enable these investigations, we synthesized an amine-functionalized diABZI analog, diABZI-NH_2_ (**1**), which provides a versatile precursor for further functionalization with other reactive handles and ligation to diverse macromolecules (Figures S11 and S24, S28). We also synthesized a maleimide-functionalized diABZI with a valine-citrulline-PABC linker (diABZI-V/C-Mal; **5**) commonly used in antibody-drug conjugates^28,29^ to enable the synthesis of cathepsin-B-cleavable PEGylated diABZI analogues (**6** and **7**) (Scheme S2). All compounds and intermediates were characterized by ^1^H NMR, ^13^C NMR, and ESI-MS (Figures S14–S16 and S25, S29)

We validated the enzyme cleavability of diABZI-V/C-Mal via incubation with cathepsin B for 48 h followed by MALDI-MS to detect the regeneration of diABZI-NH_2_ upon enzymatic cleavage. Incubation of 50 μM diABZI-V/C-Mal with 0.2 mM cathepsin B resulted in a shift in molecular weight from 1400.8 to 801.8 Da, consistent with the molecular weight of the diABZI-NH_2_ sodium adduct (Figure S1), whereas no change in molecular weight was observed under the same conditions for diABZI-Mal **(9)**, an analogue that lacks an enzyme-cleavable spacer (Scheme S3 and Figures S17, S26, S30). We validated the activity of both diABZI-NH_2_ and diABZI-V/C-Mal in human monocyte-derived THP-1 dual reporter cells, which are engineered to secrete luciferase upon activation of interferon regulatory factor 3 (IRF3) signaling, as well as in primary murine splenocytes (Figure S2), which comprise a mixture of different immune cell populations. Both analogues activated STING with comparable potency, with lower EC_50_ values observed for diABZI-NH_2_ that can likely be attributed to differences in membrane permeability.

We synthesized diABZI-PEG conjugates by reacting diABZI-NH_2_ with commercially available MeO-PEG-NHS esters for the nucleophilic substitution of the NHS ester with diABZI-NH_2_. This yielded PEGylated diABZI variants conjugated to PEG chains of 5 **(2)** or 20 kDa **(3)** via an amide linkage (Schemes 1 and S1). To synthesize the cathepsin B-cleavable diABZI-PEG analogues, diABZI-V/C-PEG_5 kDa_ (**6**) and diABZI-V/C-PEG_20 kDa_ (**7**), diABZI-V/C-Mal was reacted with MeO-PEG_XkDa_-SH under basic conditions, where the terminal thiol attacks the maleimide group to form a thioether linkage (Scheme S2). All diABZI-PEG conjugates were characterized by ^1^H NMR (Figures S12,S13 and S15, S16).

**Scheme 1:**
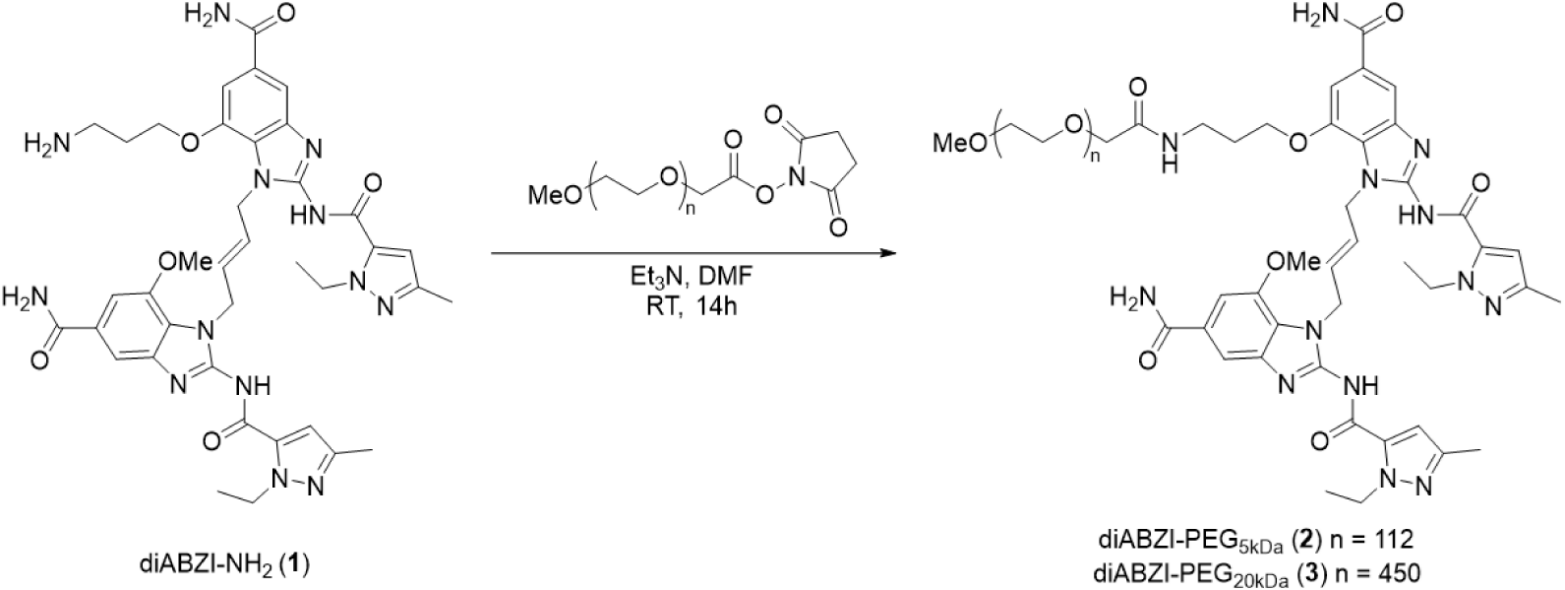
Synthesis of diABZI-PEG_5 kDa_ (**2**) and diABZI-PEG_20 kDa_ (**3**)

### PEG-diABZI Conjugates Bind and Activate STING

We first tested the activity of PEG-diABZI conjugates in THP-1 dual reporter cells relative to several small molecule analogues with different chemical substitutions at the 7-position of the benzimidazole, including a primary amine (diABZI-NH_2_), a Boc-protected amine (diABZI-Boc), hydroxyl (diABZI-OH), and morpholine (i.e., diABZI compound 3^10^) (Figure 2A,B). For small molecule diABZI analogues, we observed an expected decrease in activity with increased hydrophilicity of the substituent, with the Boc group (EC_50_ = 0.37 nM) being the most potent and the amine group (EC_50_ = 53 nM) the least. Surprisingly, however, we found that both PEG-diABZI_5 kDa_ and PEG-diABZI_20 kDa_ were active in the nanomolar range, with the 20 kDa conjugate exhibiting activity (EC_50_ = 73 nM) similar to that of diABZI-NH_2_. We next sought to characterize the effect of large diABZI modifications on binding to murine STING using a homogeneous time-resolved fluorescence (HTRF)-based competitive binding assay (Figure 2C). Interestingly, when evaluating the binding of macromolecular diABZI-PEG_20 kDa_, we did not observe a significant difference in the FRET inhibition (IC_50_ = 164 fM) compared to that of the free diABZI compound (IC_50_ = 140 fM), suggesting that the diABZI moiety can still access its STING binding domain even with a large PEG chain attached. We also found that noncleavable amide-linked analogues exhibited slightly higher activity (Figure 2A,D) than those synthesized with a cathepsin B-cleavable linker. Paradoxically, for both stable and enzyme-cleavable conjugates, activity was slightly higher for the longer 20 kDa PEG chain. In all cases, no activity was observed in STING knockout THP-1 dual reporter cells, confirming the dependence on STING for activation of IRF3 signaling (Figure S3). Together, these findings suggest that diABZI can access, bind, and activate STING even when conjugated to large PEG chains via a stable amide linkage.

**Figure 2:**
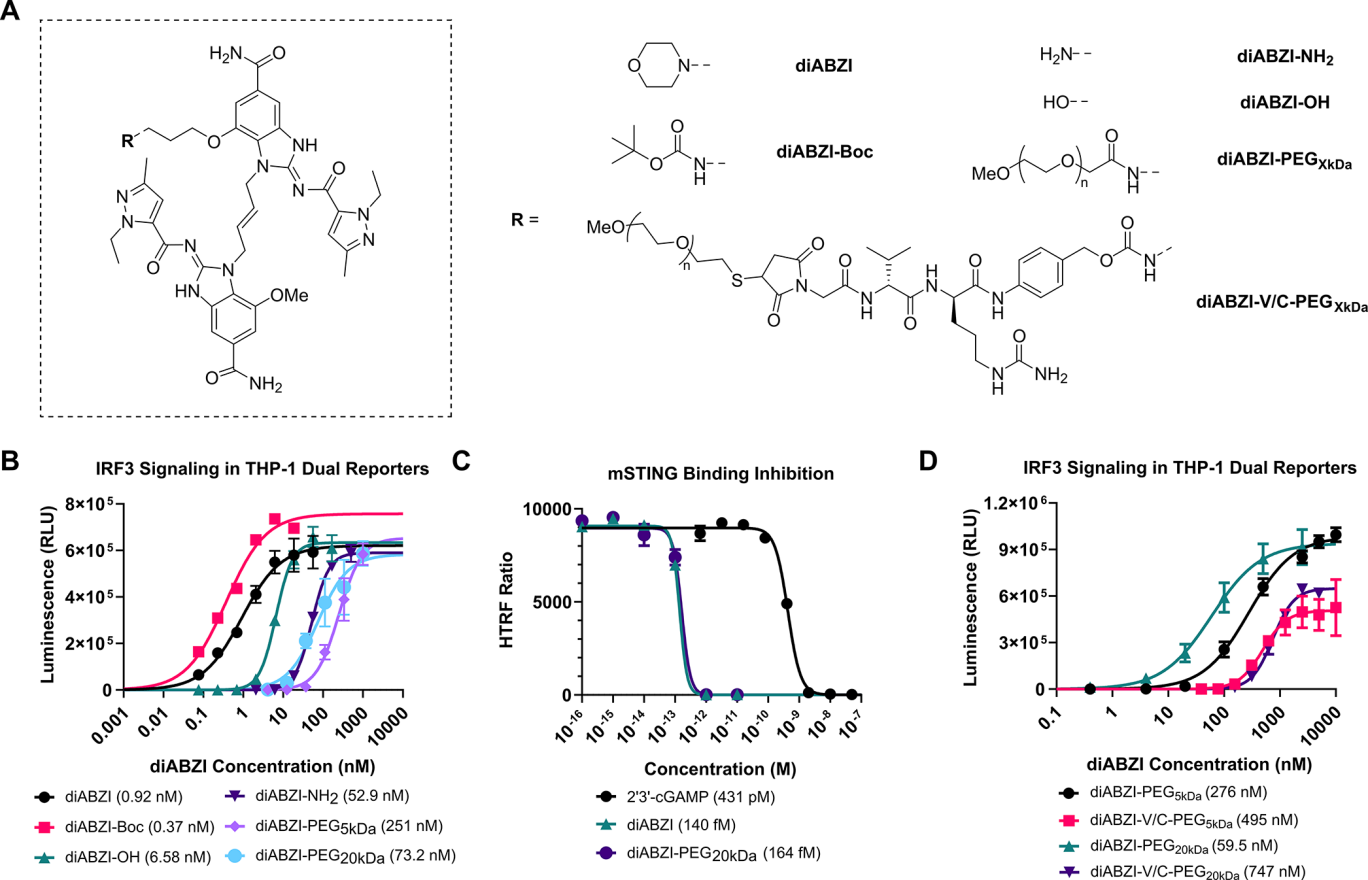
Chemical and biological characterization of polyethylene glycol (PEG)-functionalized diABZI compounds. (A) Chemical structures of diABZI (compound 3), diABZI-Boc, diABZI-NH_2_, diABZI–OH, amide-linked diABZI-PEG conjugates, and cathepsin B-cleavable diABZI-V/C-PEG conjugates. (B) Dose–response curves for relative IRF3 signaling by THP-1 dual reporter cells treated with diABZI, diABZI-Boc, diABZI-NH_2_, diABZI-OH, or amide-linked diABZI-PEGs (*n* = 3). (C) Inhibition curves from the HTRF STING binding assay to determine IC_50_ for binding of diABZI-PEG_20 kDa_ to murine recombinant STING; commercially available diABZI (compound 3) and 2′3′-cGAMP were used as controls. (D) Dose–response curves for relative IRF3 signaling by THP-1 dual reporter cells treated with cleavable and noncleavable diABZI-PEG conjugates (*n* = 3). Dose–response curves were fit to a variable slope (four-parameter) nonlinear regression to estimate EC_50_ values.

### Synthesis and Characterization of diABZI-Functionalized Polymers via RAFT Polymerization

To further validate these findings and to determine if the observed phenomenon could be extrapolated to other types of polymers with increased molecular weight, we devised a strategy for synthesizing well-defined polymers containing a single diABZI molecule linked to the end of a polymer chain via a stable amide linkage. Central to our approach was the synthesis of a diABZI-functionalized 4-cyano-4-[(ethylsulfanylthiocarbonyl)­sulfanyl]­pentanoic acid (diABZI-ECT) RAFT chain-transfer agent, which we postulated would serve as a versatile tool for controlled free-radical polymerization of bespoke polymers with well-defined properties containing a diABZI molecule on the α terminus of the polymer. To synthesize diABZI-ECT (**11**), the carboxylic acid of ECT was converted into an activated PNP ester using DCC-DMAP coupling and treated with diABZI-NH_2_ to obtain the desired product, which was characterized by ^1^H NMR, ^13^C NMR, and ESI-MS (Figure S18 and S27, S31).

We then used diABZI-ECT to synthesize a series of diABZI-functionalized *N*,*N*-dimethyl acrylamide (DMA) homopolymers (polyDMA), a biocompatible, water-soluble polymer that has been explored as a drug carrier and for the production of hydrogels.^30–32^ A distinct advantage of a CTA-based approach is the ability to synthesize diABZI-functionalized polymers with a defined degree of polymerization over a large range of molecular weights from a single diABZI molecule. Here, we synthesized diABZI-DMA with molecular weights of 25 kDa **(12)**, 50 kDa **(13)**, and 175 kDa **(14)**. The polymerization was performed in *N*,*N*-dimethylformamide (DMF) under an inert atmosphere at 70 °C at a mole ratio of initial monomer (n): diABZI-ECT: initiator of n:1:0.2 (Schemes 2 and S4). The resultant polymers were purified by extensive dialysis, lyophilized, and characterized by ^1^H NMR and GPC/LS (Figures S19–S21 and S33). Monomer conversion for the 25 kDa, 50 kDa, and 175 kDa diABZI-DMA constructs was 90.1%, 98.1%, and 99.3%, respectively. GPC/LS analysis showed monodisperse diABZI-DMA constructs, with PDI values of 1.01 for the 25 kDa variant, 1.08 for the 50 kDa variant, and 1.17 for the 175 kDa variant (Figure S33). While herein we leveraged only diABZI-ECT for synthesis of polyDMA and employed a stable amide linkage, the functionalization of RAFT chain-transfer agents with diABZI, or other STING agonists, provides a facile chemical tool for the synthesis of well-defined, physiochemically diverse polymers for activation of STING signaling.

**Scheme 2:**
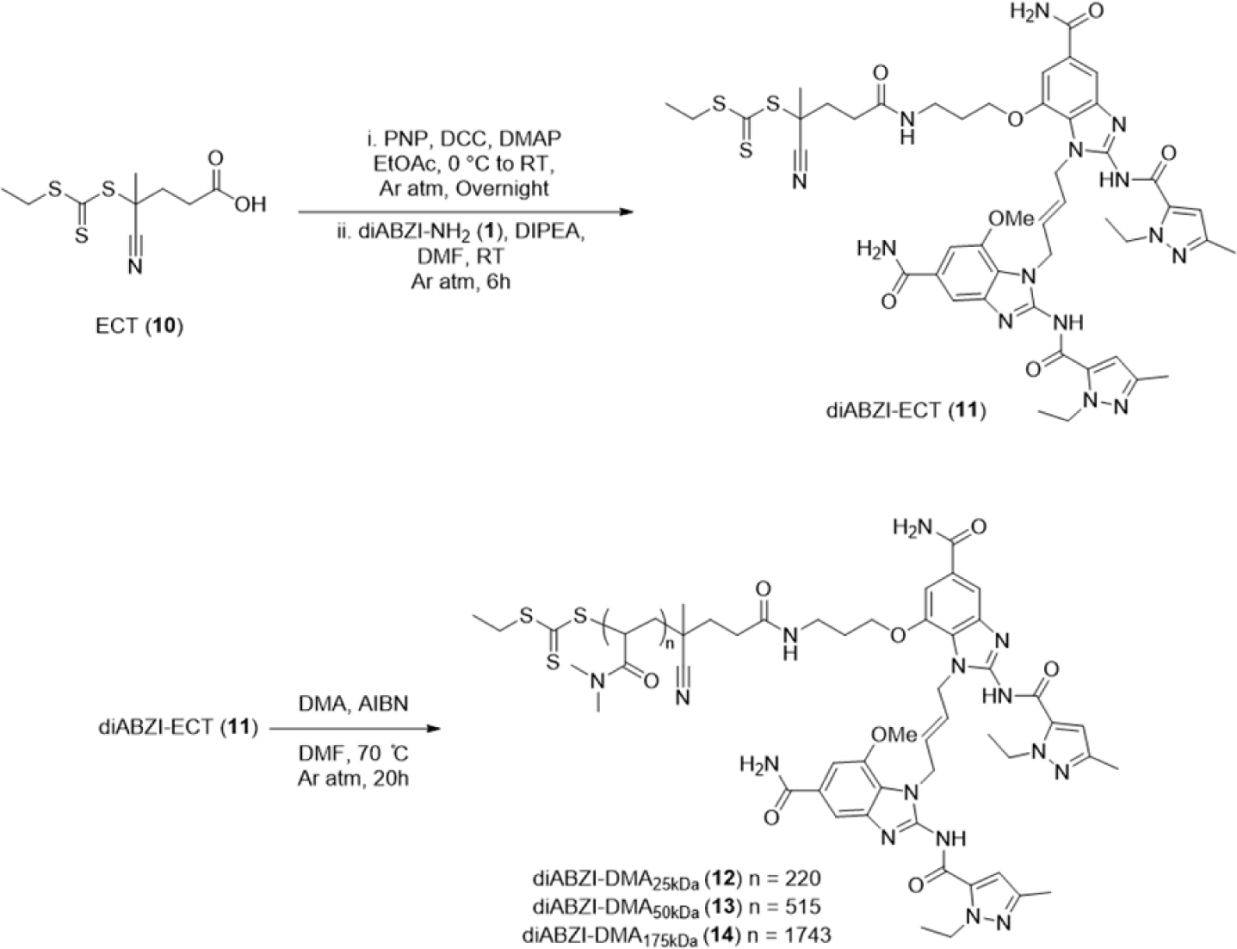
Synthesis of diABZI-ECT (**11**) and diABZI-DMA_25 kDa_ (**12**), diABZI-DMA_50 kDa_ (**13**), and diABZI-DMA_175 kDa_ (**14**)

### High Molecular Weight diABZI-DMA Conjugates Activate STING

We next evaluated the capacity of diABZI-DMA conjugates to activate STING in THP-1 dual reporter cells compared to the unpolymerized diABZI-ECT small molecule. Consistent with our findings with stable diABZI-PEG conjugates, we found that all stable diABZI-DMA constructs activated STING to a similar degree, with EC_50_ values in the 50–125 nM range, regardless of polyDMA molecular weight (Figure 3A). As expected, based on data in Figure 2B, we also found that diABZI-ECT was significantly more potent than diABZI-NH_2_, likely owing to differences in polarity and membrane permeability, though, similar to PEGylated variants, diABZI-DMA polymers exhibited comparable potency to diABZI-NH_2_. Since there were no significant differences between polymers of varying molecular weights, we selected 50 kDa diABZI-DMA (hereby referred to as diABZI-DMA) for the remaining studies. Since STING activation also activates NF-κB signaling, we utilized the dual-reporter functionality of THP-1 dual reporter cells to evaluate the relative NF-κB response, finding that both diABZI-ECT and diABZI-DMA could activate this arm of the STING pathway, with diABZI-ECT exhibiting higher potency as expected (Figure S4A,B). We also verified that responses to diABZI-ECT and diABZI-DMA were STING-dependent by evaluating activity in STING-knockout THP-1 dual reporter cells (Figure S4C,D). Collectively, these data further demonstrate that diABZI conjugated to bulky, water-soluble macromolecules via a stable amide linkage can still activate STING signaling with similar potency to the amine-functionalized parent compound, diABZI-NH_2_, despite an ∼150× difference in molecular weight.

**Figure 3:**
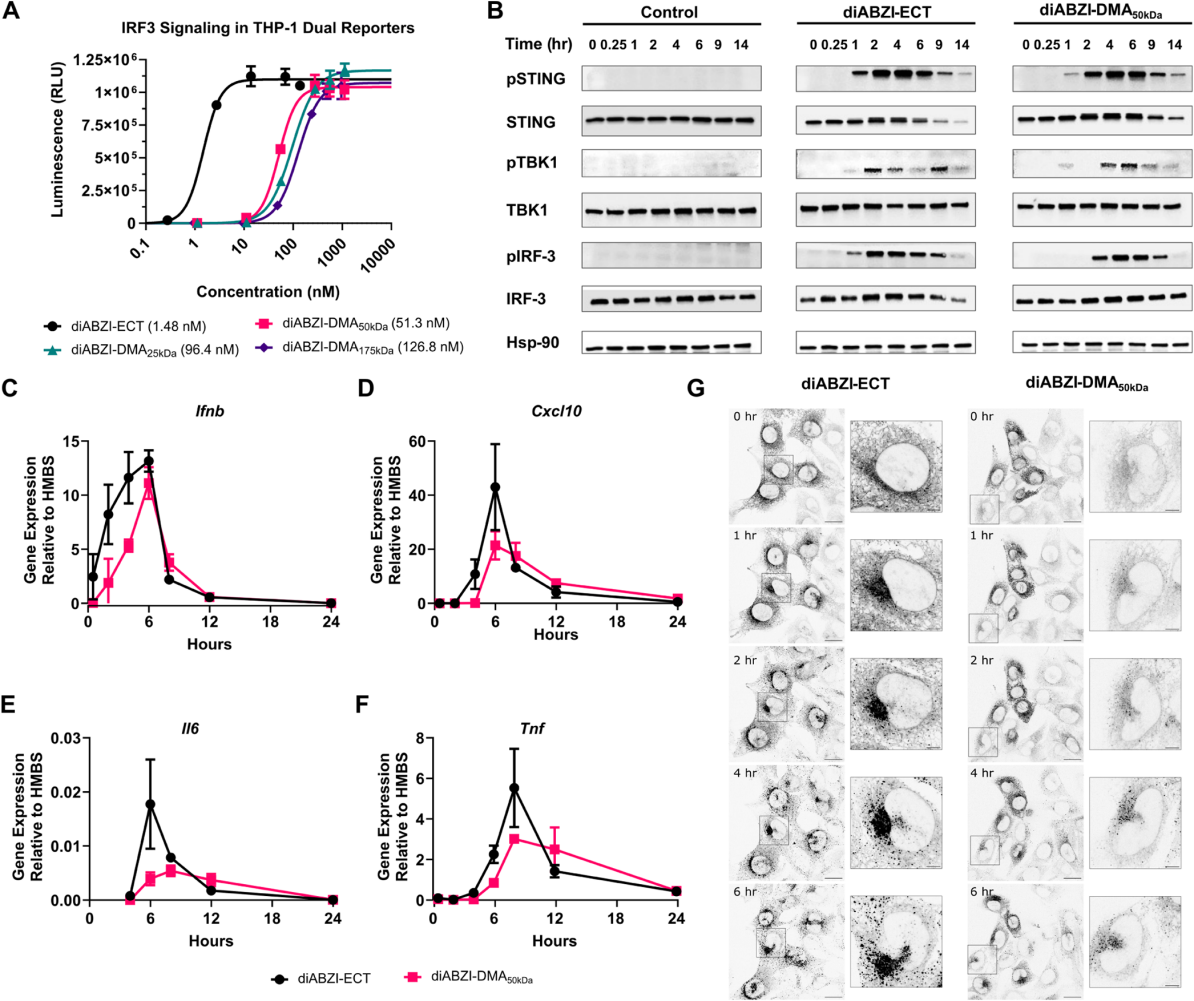
Evaluation of STING activation by diABZI-DMA conjugates. (A) Dose–response curves for relative IRF3 signaling in THP-1 dual reporter cells treated with diABZI-ECT and diABZI-DMA constructs of varying molecular weights (*n* = 3). Dose–response curves were fit to a variable slope (four-parameter) nonlinear regression to estimate EC_50_ values. (B) Western blot analysis of the STING pathway in response to treatment of THP-1 dual cells with diABZI-ECT or diABZI-DMA_50 kDa_ as a function of incubation time. (C—F) RT-qPCR of STING-associated genes in response to treatment of THP-1 dual cells with diABZI-ECT or diABZI-DMA_50 kDa_ as a function of incubation time (*n* = 3). (G) Representative fluorescence micrographs of MEF cells expressing a STING-GFP fusion protein treated with diABZI-ECT or diABZI-DMA for the indicated time. The formation of GFP puncta corresponds to multimerization of STING following ligand binding. Scale bar: 20 μm; inset: 5 μm.

We next aimed to better understand STING activation kinetics between a small molecule and a macromolecular diABZI variant, comparing diABZI-ECT and diABZI-DMA in THP-1 dual cells. RT-qPCR was utilized to measure the expression of STING-associated genes over time by THP-1 cells treated with 5 μM diABZI, a concentration exceeding the EC_50_ of both molecules (Figure 3C–F). Interestingly, the kinetics of gene expression were relatively similar between diABZI-ECT and diABZI-DMA, with gene expression of *Ifnb* and *Cxcl10* peaking around 6 h despite dramatic differences in molecular weight. Treatment with diABZI-DMA resulted in slightly delayed gene expression, with some prolongation of *Il6* and *Tnf* expression relative to diABZI-ECT, but inflammatory gene expression returned to baseline levels by 24 h in all cases. To further understand the kinetics of STING activation, we performed Western blot analysis for phosphorylated STING, TBK1, and IRF3 in THP-1 dual cells treated with 5 μM diABZI-ECT or diABZI-DMA. Following activation, STING traffics from the ER to the Golgi, where it recruits TBK1, which phosphorylates itself, STING, and IRF3.^33^ Consistent with gene expression data, we found that diABZI-DMA treatment results in slightly delayed STING activation, as measured by levels of pSTING, pTBK1, and pIRF3, which is evident within one h for both treatments and peaks between 2 and 4 h for diABZI-ECT and between 4 and 6 h for diABZI-DMA (Figure 3B). Finally, to qualitatively assess intracellular STING dynamics in the initial activation period, we employed murine embryonic fibroblasts (MEFs) expressing a STING-GFP fusion protein that has been used previously to evaluate the formation of fluorescent puncta associated with oligomerization of STING dimers on the ER following activation.^34^ After treating STING-GFP MEFs with 5 μM diABZI-ECT or diABZI-DMA, we used confocal microscopy to image relative puncta formation and GFP expression and degradation over time. Consistent with Western blot and PCR analysis, diABZI-ECT triggered puncta formation within 1 to 2 h, whereas this was slightly delayed with diABZI-DMA, which nonetheless activated STING within 2 to 4 h (Figure 3G). Collectively, these studies demonstrate that diABZI-DMA, linked via a stable amide bond, can activate STING with similar, though slightly delayed, kinetics relative to small-molecule diABZI compounds.

### diABZI-DMA Conjugates Are Endocytosed and Activate STING as Stable Macromolecules

Intrigued by the findings that diABZI-DMA conjugates could rapidly and potently activate STING, we further evaluated their intracellular uptake and localization using sCy5- and Cy5-labeled variants of diABZI-NH_2_ (**16**) and diABZI-DMA (**18**) synthesized as described in Scheme S5 and S6 and characterized in Figures S22,S23, S32, and S34. The activity of Cy5-labeled variants in THP-1 dual reporter cells is confirmed in Figure S5. First, using flow cytometry, we found that cellular uptake of diABZI-DMA-Cy5 is nearly entirely inhibited when incubated at 4 °C (Figure 4B), indicating internalization via an active endocytic process, which was expected for a large hydrophilic macromolecule that cannot diffuse through the cell membrane. By contrast, uptake of diABZI-sCy5 was only partially inhibited at 4 °C, consistent with passive membrane diffusion of small-molecule diABZI compounds that may have been decreased here due to reduced membrane permeability at lower temperatures and/or labeling with sCy5, which reduces hydrophobicity and membrane permeability (Figure 4A). This was further assessed by confocal microscopy of STING-GFP MEF cells treated with diABZI-DMA-Cy5 or diABZI-sCy5 and stained with LysoTracker to identify late endosomes and lysosomes, where STING ultimately traffics and is degraded following activation.^33^ Colocalization of diABZI-DMA-Cy5 with LysoTracker was significantly higher than that of diABZI-sCy5, further demonstrating that polymer-diABZI conjugates are internalized by endocytosis to a greater extent than small-molecule diABZI compounds, which can enter cells via passive diffusion across the cell membrane (Figure S6).

**Figure 4:**
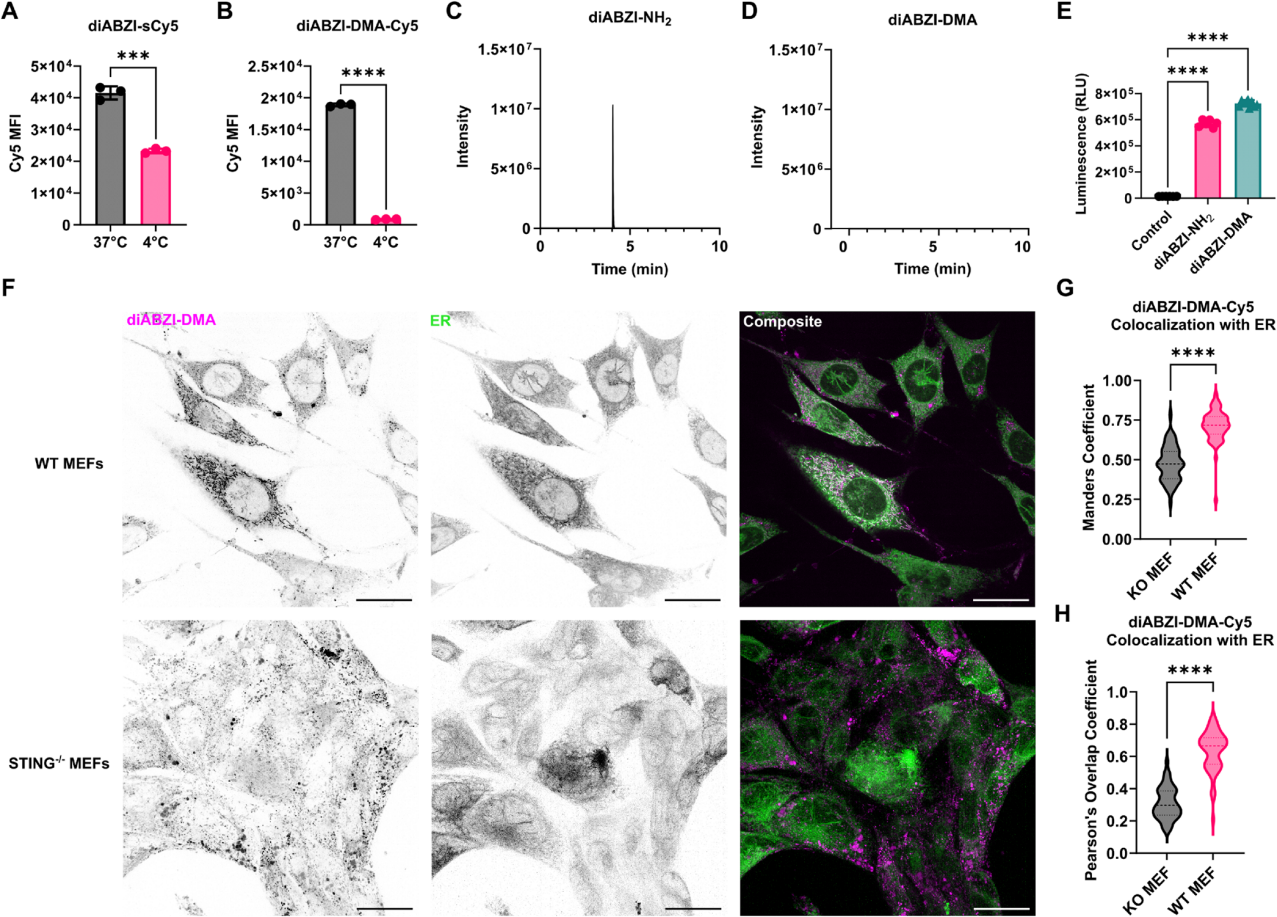
Intracellular uptake and ER localization of diABZI-DMA conjugates. (A and B) Flow cytometry analysis of THP-1 dual cell uptake of diABZI-sCy5 and diABZI-DMA-Cy5 at 37 and 4 °C to determine dependence on endocytosis (*n* = 3). (C and D) LC-MS/MS spectra for diABZI-NH_2_ adduct following 6 h incubation with diABZI-DMA and diABZI-NH_2_ treatments in THP-1 dual cells at 37 °C, demonstrating cellular stability of diABZI-DMA. (E) Relative IFN-I production by THP-1 dual reporter cells treated with diABZI-NH_2_ and diABZI-DMA constructs (*n* = 6 – 9). (F) Representative images of diABZI-DMA-Cy5 colocalization with the ER in wild-type and STING^–/–^ MEFs after 2 h treatment. Cells were stained with ER Red. Colocalization between Cy5-labeled diABZI variants and ER Red stain was quantified via (G) Manders and (H) Pearson’s coefficients. Scale bars, 25 μm. All data were plotted using GraphPad 10 and evaluated for statistical significance via an unpaired *t* test or one-way ANOVA with Tukey’s multiple comparisons test. *p < 0.05, **p < 0.01, ***p < 0.001, ****p < 0.0001.

While amide bonds on polymer carriers are considered to be biologically stable,^35,36^ particularly in the 1 to 4 h time frame during which diABZI-DMA activates STING, we evaluated the stability of diABZI-DMA in tritosomes—lysosomes isolated from the rat liver that are commonly used to investigate drug and conjugate stability and catabolism. We used MALDI mass spectrometry to assess the emergence of diABZI-NH_2_, which would be the product if the amide bond linking diABZI to the polymer backbone were cleaved by lysosomal proteases. Even after incubation of diABZI-DMA with tritosomes for 7 h, we did not observe the formation of peaks that would correspond to liberated diABZI; however, diABZI-NH_2_ remains detectable using MALDI from 0 to 7 h of incubation with tritosomes at a matched molar concentration (Figure S7). We also incubated THP-1 dual cells with diABZI-NH_2_ or diABZI-DMA at 60 μM for 6 h, followed by washing, cell lysis, and methanol-based extraction of free diABZI-NH_2_ for quantification by LC-MS/MS (Figure 4C,D). While we clearly observed the presence of diABZI-NH_2_ in cells treated with diABZI-NH_2_, nearly undetectable levels were found (∼0.04% of what was measured in cells treated with diABZI-NH_2_) in cells treated with diABZI-DMA, consistent with tritosome stability data. We also assayed the culture supernatant for relative IFN-I response via detection of secreted luciferase IRF3 reporter, again finding that diABZI-DMA conjugates activated STING at levels similar to or greater than those of diABZI-NH_2_ (Figure 4E). Together, these data indicate that endocytosed diABZI-DMA remains stable and is not rapidly degraded within endolysosomes, and, therefore, STING activation can be induced by diABZI-DMA as an intact macromolecule rather than by a released small-molecule diABZI byproduct that can passively diffuse across biological membranes to activate STING.

### diABZI-DMA Conjugates Target the ER to Activate STING

STING primarily resides on the ER membrane, where binding to diABZI or other agonists triggers its translocation to the Golgi apparatus, thereby recruiting TBK1 to phosphorylate STING and IRF3 and subsequently trafficking on vesicles to late endosomes and lysosomes, where STING is ultimately degraded. However, there is also evidence that STING does not remain stationary on the ER and instead moves continuously back and forth between the ER and the Golgi and may even require translocation to late endosomal membranes to activate IRF3.^33,37^ While mechanisms of STING trafficking and signaling are not completely understood, given the complex dependence on vesicular transport processes, it is conceivable that endocytosed STING agonists could ultimately reach the ER. Indeed, in STING-GFP MEF cells, treatment with diABZI-DMA results in the formation of puncta spatially distributed near the nucleus, consistent with multimerization of STING on the ER membrane (Figure 3G). To further investigate this, we quantified the degree of colocalization of diABZI-sCy5 and diABZI-DMA-Cy5 with the ER in STING-GFP MEF cells using an ER Tracker Red stain. As anticipated based on its more potent activity, we observed that diABZI-sCy5 colocalized with the ER to a greater degree than diABZI-DMA-Cy5, which also appeared distributed as fluorescent puncta surrounding the nucleus, with a Mander’s overlap coefficient with the ER stain of >0.5 (Figure S8). To determine if this colocalization pattern was STING-dependent, we evaluated diABZI-DMA-Cy5 colocalization with the ER in wild-type and STING-KO MEF cells, finding a significant decrease in Cy5-ER colocalization in STING-KO MEF cells via Manders and Pearson’s coefficients (Figure 4F–H).

Taken together, these findings suggest that at least a subset of endocytosed diABZI-DMA can access, bind to, and activate STING on the ER as an intact macromolecule. While polyDMA is not expected to induce endosomal disruption that would enable the escape of diABZI-DMA from endolysosomal confinement, we nonetheless validated this using a well-established galectin-8 (Gal8) recruitment assay^38^ used to test the endosomal escape properties of drug carriers (e.g., LNPs). As expected, we did not observe any evidence of endosomal destabilization that would enable diABZI-DMA to access the cytosol (Figure S9). Additionally, based on recent evidence that the CDN transport protein LRRC8A plays a role in fluxing CDNs from endolysosomes and phagosomes into the cytosol,^39^ we evaluated the activity of diABZI-DMA in LRRC8A knockout or vector control immortalized bone marrow-derived macrophages (iBMDM) but found no significant difference in IFN-β secretion, indicating that LRRC8A is unlikely to provide a route for cytosolic access (Figure S10). Therefore, much like mechanisms of CDN transport and the STING pathway itself, the mechanisms by which macromolecular diABZI adducts can access, bind, and activate STING remain dynamic and complex and are yet to be fully elucidated. Nonetheless, our data provide evidence that endocytosed, stably linked polymer-diABZI conjugates can still activate STING, a finding with potentially important implications for the rational design of drug carriers for STING agonists.

### Macromolecular diABZI Conjugates Are Therapeutically Active in Mouse Tumor Models

Since STING agonists are being actively explored as cancer immunotherapeutics and diABZI-based agonists (e.g., GSK3745417) have entered clinical trials (NCT03843359), we next sought to validate the antitumor activity of diABZI-PEG and diABZI-DMA constructs in mouse tumor models. First, we compared the efficacy of diABZI-NH_2_ and diABZI-PEG_20 kDa_ in a B16-F10 melanoma murine tumor model, a poorly immunogenic model commonly employed in preclinical immunotherapy development. After establishing ∼50 mm^3^ subcutaneous B16-F10 tumors, mice were intravenously injected with either PBS (vehicle), diABZI-NH_2_, or diABZI-PEG_20 kDa_ every 3 days for a total of 3 treatments at 0.035 μmol diABZI/mouse. Tumor volume was measured until day 18 when the first tumor reached 1500 mm^3^. Consistent with their similar *in vitro* activity, both molecules inhibited tumor growth to a comparable degree and were well tolerated (Figure 5A–D). We evaluated the efficacy of diABZI-DMA in a model of peritoneal metastasis in which luciferase-expressing MC38 colon cancer cells are injected intraperitoneally to seed tumor nodules throughout the peritoneal cavity. Mice were administered diABZI-ECT or diABZI-DMA (0.007 μmol diABZI) intraperitoneally on days 7 and 10 following tumor inoculation, and tumor burden was evaluated by quantification of excised tumor mass on day 14 (Figure 5E,F). Both diABZI-ECT and diABZI-DMA were effective in inhibiting tumor growth, with diABZI-ECT exhibiting greater, but statistically insignificant, antitumor effects. Hence, these studies demonstrate that stable macromolecular diABZI-PEG and diABZI-DMA conjugates can activate STING *in vivo*.

**Figure 5:**
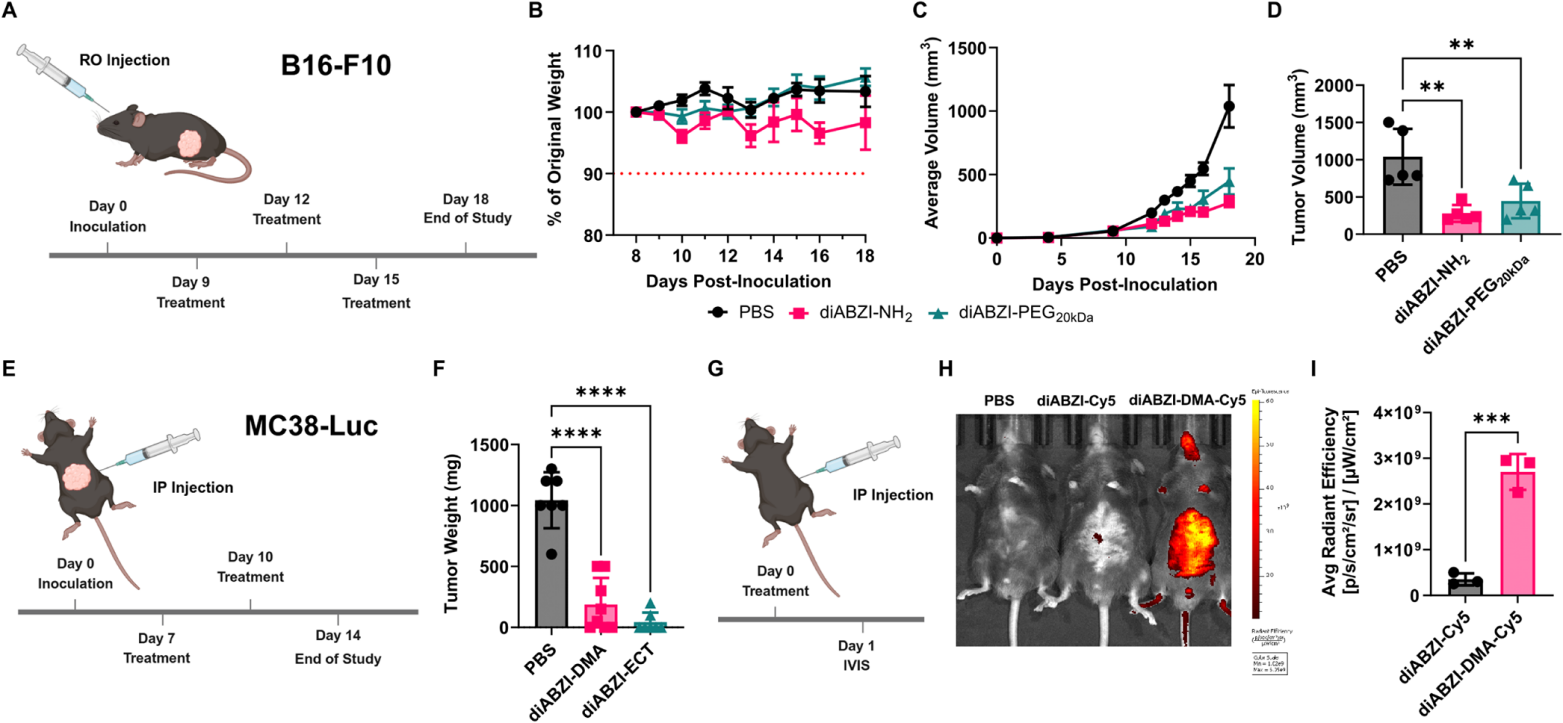
*In vivo* activity of macromolecular diABZI conjugates in tumor models. (A) Schematic of subcutaneous B16-F10 tumor inoculation, treatment schedule, and study end point for evaluation of the therapeutic activity of diABZI-NH_2_ or diABZI-PEG_20 kDa_ relative to vehicle (PBS) treatment. (B and C) Average weight loss and tumor volume growth curves for mice with B16-F10 melanoma tumors treated as indicated. (D) B16-F10 melanoma tumor volumes on day 18 (*n* = 5). (E) Schematic of intraperitoneal MC38-Luc tumor inoculation, treatment schedule, and study end point for evaluation of the therapeutic activity of diABZI-DMA and diABZI-ECT relative to vehicle (PBS) treatment. (F) Average weight of MC38-Luc tumors retrieved from the peritoneal cavity on day 14 post-inoculation (*n* = 7–8). (G) Schematic and study timeline for the intraperitoneal administration of diABZI-sCy5 and diABZI-DMA-Cy5. (H) Representative IVIS images of mice 24 h post-injection of either diABZI-sCy5 or diABZI-DMA-Cy5. (I) Quantitative analysis of Cy5 fluorescence 24 h post-injection of diABZI-sCy5 and diABZI-DMA-Cy5 (*n* = 3). All data were plotted using GraphPad 10 and evaluated for statistical significance via a one-way ANOVA with Tukey’s multiple comparisons test. *p < 0.05, **p < 0.01, ***p < 0.001, ****p < 0.0001.

The similar degree of efficacy observed between diABZI-ECT and diABZI-DMA may also reflect an interplay between potency and pharmacological properties that impact the magnitude and kinetics of STING activation. Notably, diABZI-ECT is more potent than diABZI-DMA (Figure 3A) owing to its high membrane permeability, while the higher molecular weight of diABZI-DMA may allow for increased half-life, modulated clearance rates and mechanisms, and/or altered cellular and tissue distribution profiles. For example, we found that intraperitoneally administered diABZI-DMA-Cy5 had an intraperitoneal residence time longer than that of diABZI-sCy5 (Figure 5G–I). Importantly, the objective of these studies was only to validate the *in vivo* activity of stable macromolecular diABZI agonists rather than to engineer constructs for maximal antitumor efficacy or to investigate the impact of STING activation on antitumor immunity or the tumor microenvironment, which we and others have described previously across a range of tumor types.^17,40–42^ However, these findings motivate future work focused on leveraging both stable and cleavable conjugates to optimally balance diABZI potency and pharmacological properties for disease-specific applications.

## Discussion

The conjugation of diABZI STING agonists to drug carriers, including polymers, nanoparticles, and proteins, holds great potential for improving their efficacy and/or safety in cancer immunotherapy and other biomedical applications.^16–18,25,43,44^ This is exemplified by a growing number of reports over the past several years leveraging diABZI variants with conjugatable handles, including the development of antibody-drug conjugates (ADCs) currently in clinical trials (XMT-2056)^43^ as well as polymer-drug conjugates recently reported by our group^18^ and others.^16^ While antibodies and other protein carriers can be readily degraded within lysosomes to release an active diABZI compound,^17,19^ many promising polymeric carriers (e.g., PEG) are not efficiently degraded, motivating the use of cleavable linkers to ensure diABZI release. Here, we report the unexpected finding that diABZI linked to large, hydrophilic, and nonbiodegradable polymer chains via amide linkages can activate STING with comparably rapid kinetics to unconjugated diABZI agonists despite a dependence on endocytosis for cellular internalization. This is broadly consistent with recent reports describing the conjugation of dyes, PET probes, and peptides to diABZI via noncleavable linkers, which can retain binding and activity,^23,25,45^ though the polymers we employed (PEG and polyDMA) were of significantly higher molecular weight. This property may be a distinctive feature of diABZI since other STING agonists, such as 2′3′-cGAMP or MSA-2, trigger a closed-lid conformation that may preclude the addition of steric bulk; additionally, these molecules lack sites for chemical modification that are not also essential for STING binding.^22^

Here we found that diABZI, linked to the end of the RAFT-synthesized 50 kDa polyDMA chain via an amide linkage, activated STING in THP-1 dual reporter and MEF cells within ∼1 h. As expected for a large molecule, diABZI-DMA was dependent on endocytosis for cellular internalization, resulting in trafficking to endolysosomes. However, we could not detect the presence of a liberated diABZI-NH_2_ compound that would be anticipated upon amide bond cleavage, either in cells treated with diABZI-DMA or following incubation of diABZI-DMA with tritosomes. This suggests that the rapid STING activation observed was not a result of diABZI release. While it is conceivable that the extent of diABZI release was below the limit of detection by our LC-MS/MS and MALDI methods, trace amounts of diABZI-NH_2_ are unlikely to contribute significantly to the activity of diABZI-DMA given the comparable EC_50_ of the compounds. Interestingly, we found that internalized diABZI-DMA constructs exhibited appreciable colocalization with the ER, where STING is localized, and this was reduced in STING-KO cells, further supporting a model in which diABZI-DMA accesses and binds to STING as an intact molecule. This phenomenon may be comparable to a recent report demonstrating that diABZI could be harnessed as an ER-targeting ligand when conjugated to peptide antigens.^25^

STING is a unique pattern recognition receptor because it is dynamically trafficked throughout the endomembrane system to regulate immunostimulatory activity.^33,46^ In addition to the established ER-to-Golgi transport that occurs following STING activation and multimerization, STING also traffics to lysosomes where it is degraded, though there is evidence that interferon signaling by STING requires translocation to late endosomes.^37^ Additionally, there is evidence supporting a “basal flux” model of STING trafficking, where it continuously moves from the ER to lysosomes at homeostasis, including a continuous movement between the ER and Golgi.^33^ There are even reports of a STING isoform that resides on the plasma membrane.^47^ While much remains to be learned about the mechanisms of intracellular STING transport, its vesicular distribution pattern may enable interaction with diABZI-functionalized macromolecules that contribute to STING activation. Notably, both endogenous and endocytosed molecules can be transported from endosomes to the ER through membrane contact sites and retrograde transport pathways.^48,49^ While, as expected, diABZI-DMA did not induce endosomal disruption, and our study did not implicate the CDN transporter LRRC8A,^39^ it is also possible that a small fraction of endocytosed diABZI-DMA leaks from endolysosomes into the cytosol, where it can directly access and bind STING. Like the STING pathway itself, additional investigation is necessary to understand the mechanisms by which diABZI-DMA and diABZI-PEG can activate STING—studies that may ultimately be enabled using stable polymer-diABZI conjugates as a research tool.

Our study also has potential implications for the emergent design of polymer-diABZI conjugates. First, while PEG-diABZI and diABZI-DMA can activate STING, it should be noted that their *in vitro* activities are ∼50–500-fold lower than those of small molecule diABZI variants with hydrophobic substitutions (e.g., Boc, morpholine) at the conjugation site. This bodes favorably for the design of diABZI-carrier conjugates with cleavable linkers to confer prodrug-like properties that enable environmentally- or stimuli-responsive release of a potent diABZI analog. While we ultimately believe that such approaches will prove more therapeutically valuable, stable polymer-diABZI conjugates may offer some advantages. For example, macromolecular STING agonists with lower potency but favorable pharmacological behaviors conferred by carrier properties may provide a route to improved efficacy or safety in some indications. Notably, we observed that the increased residence time of diABZI-DMA conferred comparable antitumor activity to a highly potent small molecule diABZI analogue in a model of peritoneal metastasis. Additionally, stable polymer-diABZI conjugates may also serve as carriers for targeting cargo to the ER via STING binding. Finally, the site-selective conjugation strategies employed yielded well-defined polymer-diABZI conjugates that were directly and readily water-soluble at physiological pH, which may afford a practical advantage for drug formulation and intravenous administration, as solubilization of hydrophobic diABZI compounds requires the use of viscous and/or organic excipients that may not be suitable for clinical use and/or increase the risk of adverse effects (e.g., Cremophor).^50^

## Conclusion

Dimeric amidobenzimidazole (diABZI) STING agonists hold promise as cancer immunotherapeutics, vaccine adjuvants, and antiviral agents, motivating the recent exploration of diABZI-polymer conjugates to modulate their pharmacological properties to improve efficacy and/or safety. Central to this effort is the design and synthesis of diABZI analogues with reactive handles for covalent ligation to carriers and drug linkers to modulate drug release, stability, and/or bioavailability. Here, we report the surprising finding that diABZI compounds linked to multiple highly water-soluble and large (up to 175 kDa) polymers via a stable amide bond can rapidly bind and activate STING both *in vitro* and *in vivo*. We demonstrate that while such macromolecular diABZI constructs enter cells through endocytosis, they can localize to the ER to bind STING, and this does not appear to be dependent on drug release and subsequent delivery to the cytosol. While the biological mechanisms through which this occurs remain to be elucidated, our studies support an alternative pathway whereby intracellular trafficking of diABZI-polymer conjugates may be harnessed to access and activate STING. This finding has potentially important implications for the continued design of polymer-drug conjugates, linkers, and prodrugs for STING activation and serves to motivate the exploration of diABZI-carrier conjugates with stable linkers for certain therapeutic applications.

## Experimental Methods

### Molecule Synthesis (Additional Details Provided in Supporting Information)

We synthesized diABZI-NH_2_ (**1**) with a reactive primary amine handle, enabling it to be tethered to macromolecules through stable bonds that do not release small-molecule diABZI analogues. To achieve the desired macromolecules, we employed two distinct strategies. In the first strategy, we synthesized stable analogues of diABZI-NH_2_ by reacting it with activated *N*-hydroxysuccinimide (NHS) esters of MeO-PEG_XkDa_-NHS, producing two analogues: diABZI-PEG_5 kDa_ (**2**) and diABZI-PEG_20 kDa_ (**3**). The primary amine group of diABZI-NH_2_ attacks the carbonyl group of the activated ester, forming an intermediate product with a tetrahedral structure. Subsequently, a more labile leaving group is released, resulting in the formation of a stable secondary amide through nucleophilic substitution. In the second strategy, we reacted diABZI-NH_2_ with the activated PNP ester of a chain-transfer agent (**10**), forming a diABZI-based chain-transfer agent analogue (**11**). This analogue was used to synthesize macromolecules **12** through **14** via RAFT polymerization in a controlled manner.

### Cathepsin B Activity Assay

Recombinant mouse cathepsin B (R&D Systems) was prepared at 50 μM in MES buffer (pH 5.0) upon opening and stored at −80 °C. To activate the enzyme, cathepsin B was diluted to 0.2 μM in MES buffer (pH 5.0) containing 1 mM EDTA and 2 mM DTT and placed at 37 °C for 15 min. After activation, cathepsin B was combined with 50 μM of substrate at 37 °C for 48 h at a total volume of 100 μL. Activity was determined by observing molecular weight shifts in the substrate using matrix-assisted laser desorption and ionization mass spectrometry (MALDI-MS).

### MALDI-MS

A 4 μL portion of matrix (20 mg mL^–1^ THAP and 20 mg mL^–1^ CHCA in dry acetone) was combined with 1 μL of sample from the cathepsin B activity assay and spotted on a stainless steel MALDI-MS plate (Bruker). After evaporation of the matrix, three technical replicates were collected for each sample using FlexControl software (Bruker Daltonics) on a Bruker AutoFlex MALDI-TOF. The laser pulse rate was 1000 Hz, and spectra were obtained with a mass window of 600–5000 *m*/*z* at high resolution (4.00 GS/s). FlexAnalysis software (Bruker Daltonics) was used to perform unbiased smoothing and to obtain baseline spectra for all samples.

### THP-1 Dual Reporter Cell Assay

THP-1 dual cells and STING-knockout THP-1 dual cells (InvivoGen) were cultured in Roswell Park Memorial Institute (RPMI) 1640 Medium (Gibco) supplemented with 2 mM l-glutamine, 25 mM HEPES, 10% heat-inactivated fetal bovine serum (HI-FBS; Gibco), 100 U ml^–1^ penicillin/100 μg mL^–1^ streptomycin (Gibco), and 100 μg/mL Normocin. Cells were subjected to 10 μg/mL blasticidin and 100 μg/mL Zeocin for continual selection after every cell passage. 96-well plates (REF 655180; Greiner Bio-One) were used for screening agonist activity. Reporter cells were seeded at 25,000 cells/well in 100 μL media, and treatments were administered in 100 μL of medium. Results were collected 24 h after treatment using a Quanti-Luc or Quanti-Blue (InvivoGen) assay on cell supernatants following manufacturer’s instructions. Luminescence was quantified using a plate reader (Synergy H1Multi-Mode Microplate Reader; Biotek) after supernatants were transferred to opaque-bottom 96-well plates (REF 655073; Greiner Bio-One).

### Splenocyte Isolation and IFN-β Reporter ELISA

Spleens were harvested from female C57BL/6 mice (8 weeks old), mechanically disrupted into single-cell suspensions through a 70 μm cell strainer (Fisherbrand; Thermo Fisher Scientific), and suspended in complete RPMI 1640 medium (Gibco) supplemented with 10% FBS, 10% HI-FBS (Gibco), 100 U ml^–1^ penicillin/100 μg mL^–1^ streptomycin (Gibco), 50 μM 2-mercaptoethanol, and 2 mM l-glutamine. The cells were centrifuged for 5 min at 1500 rpm and resuspended in ACK lysis buffer (KD Medical) for 5 min. Cells were centrifuged again and resuspended in fresh media at a concentration of 3 million cells per mL. Cells were seeded in a 96-well round-bottom plate at 100 μL per well, and treatments were administered in 100 μL of medium. Results were collected 24 h after treatment using a mouse IFN-β solid-phase sandwich ELISA kit (Invivogen Cat#42400-1) on cell supernatants following manufacturer’s instructions. Luminescence was quantified using a plate reader (Synergy H1 Multimode Microplate Reader; Biotek).

### mSting Binding Inhibition Assay

A HTRF murine STING WT binding competitive assay kit (Revvity) was utilized to evaluate the IC_50_ values for diABZI and diABZI-PEG_20 kDa_. The assay was performed in a 20 μL volume in a round-bottom, low-volume white 384-well plate (Sigma) according to the manufacturer’s instructions and incubated in the dark overnight to ensure equilibrium binding was reached. HTRF readouts were performed on the Synergy Neo2 plate reader with the following settings: excitation filter at 330 nm with a gain of 120, dual emission filters at 620 and 665 nm with a gain of 128, 50 measurements per data point, 8.25 mm read height, xenon flash with high sensitivity, 50 μs delay, and 500 μs data collection time. Commerical diABZI molecule compound 3 was purchased through Selleck Chem (Catalog No.S8796).

### RAFT Polymerization of diABZI-DMA Polymers (12–14)

Reversible addition–fragmentation chain transfer (RAFT) polymerization was used to synthesize analogues of polymers with distinct molar masses. For synthesis, *N,N*-dimethyl acrylamide (DMA) monomer was filtered over activated alumina and allowed to react under an inert atmosphere in DMF (30 wt % monomer) at 70 °C for 24 h in an oil bath. The initial monomer ([M]_o_) to diABZI-ECT compound **11** ([CTA]_o_) to initiator ([I]_o_) ratio was n:1:0.2. The resultant desired polymers, DMA­(nkDa)-diABZI-ECT, were isolated by dialysis against pure acetone (2×) and pure deionized water (2×). Following dialysis, the purified compound was frozen at −80 °C for 8 h and then lyophilized for 3 days. Final polymers were characterized by ^1^H NMR and GPC (TOSOH) in HPLC-grade DMF with 0.01 M LiBr via a LenS3Multi-Angle Light Scattering Detector (TOSOH).

### THP-1 Dual qRT-PCR

THP-1 dual cells (InvivoGen) were cultured in Roswell Park Memorial Institute (RPMI) 1640 Medium (Gibco) supplemented with 2 mM l-glutamine, 25 mM HEPES, 10% heat-inactivated fetal bovine serum (HI-FBS; Gibco), 100 U ml^–1^ penicillin/100 μg mL^–1^ streptomycin (Gibco), and 100 μg/mL Normocin. Cells were subjected to 10 μg/mL blasticidin and 100 μg/mL Zeocin for continual selection after every cell passage. 96-well plates (REF 655180; Greiner Bio-One) were used for screening agonist activity. Reporter cells were seeded at 300000 cells/well and treated with 5 μM drug. Cell pellets and RNA were extracted using the Qiagen RNeasy Plus Mini Kit. An iScript cDNA synthesis kit (Bio-Rad) was used to synthesize cDNA per manufacturer’s protocol. RT-qPCR on the cDNA was performed using TaqMan gene expression kits (primer and master mix) and run on the Bio-Rad CFX Connect Real-Time System, with threshold cycle number determination made by Bio-Rad CFX Manager software v3.0. Primers used included mouse Ifnb1 (Mm00439552_s1), mouse Tnf (Mm00443258_m1), mouse Cxcl10 (Mm00445235_m1), mouse IL-6 (Mm00446190_m1), and mouse Hmbs (Mm01143545_m1). Gene expression relative to Hmbs was calculated using 2^–(Cq‑CqHmbs)^.

### Western Blot Analysis

Cells were lysed with 50 μL of RIPA buffer (Sigma-Aldrich) supplemented with protease inhibitors (Sigma-Aldrich). Protein concentration was measured by using a BCA protein assay kit (Thermo Scientific). Equal amounts of protein (10–30 μg) were loaded onto SDS/PAGE and transferred onto nitrocellulose membranes using the semidry transfer protocol (Bio-Rad Laboratories). After transfer, membranes were probed with each respective primary antibody (anti-STING, anti-p-STING, anti-TBK1, anti-p-TBK1, anti-IRF3, anti-p-IRF3, and anti-HSP90) overnight at 4 °C. All antibodies were purchased from Cell Signaling. Following incubation, the membranes were probed with HRP-conjugated secondary antibodies. Protein bands were visualized using an ECL Western blotting substrate (Thermo Scientific). Images of immunoblots were obtained by using an LI-COR Odyssey Imaging System.

### Confocal Microscopy for STING Kinetics

STING-GFP murine embryonic fibroblasts (MEFs)^34^ (provided by N. Yan, UT Southwestern) were grown in complete DMEM medium supplemented with 10% (v/v) heat-inactivated fetal bovine serum (HI-FBS; Gibco), 2 mM l-glutamine, and 100 U mL^–1^ penicillin/100 μg mL^–1^ streptomycin (Gibco). Cells were seeded at 2 × 10^5^ cells per dish on fibronectin-coated 4-chamber 35 mm Cellvis dishes and incubated overnight. Cells were washed with PBS, and the medium was replaced with complete FluoroBrite DMEM (Gibco). Immediately following imaging of the zero-hour time point, cells were dosed with 10 μM drug (2× concentration) in FluoroBrite DMEM for a final concentration of 5 μM. Live-cell imaging was performed on a Nikon Ti2 inverted light microscope equipped with a Yokogawa CSU-W1 spinning disk head, a Photometrics Prime 95B sCMOS camera, four excitation lasers (488, 568, 647, and 405 nm), and a 100*X*/1.49 NA TIRF oil immersion objective. Cells were maintained in a Tokai Hit stage-top incubator at 37 °C with 5% CO_2_. Images presented in the figures were deconvolved using Nikon Elements software (3D-Deconvolution Richardson-Lucy, 12–20 iterations) and analyzed through ImageJ.

### RAFT Polymerization for diABZI-DMA-AzPMAm (18)

For synthesis, DMA was filtered over activated alumina, combined with a solution of diABZI-ECT, AIBN, and AzPMAm, and allowed to react under an inert atmosphere in DMF (30 wt % monomer) at 70 °C for 24 h in an oil bath. The initial DMA/AzPMAm/diABZI-ECT/AIBN ratio was 496:11:1:0.2. The resultant desired polymer was isolated by dialysis against pure acetone (2×), 1:1 pure acetone and deionized water (1×), and then pure deionized water (2×). Following dialysis, the purified compound was frozen at −80 °C for 8 h and then lyophilized for 72 h. It was further characterized by ^1^H NMR and GPC (TOSOH) in HPLC-grade DMF with 0.01 M LiBr via the LenS3Multi-Angle Light Scattering Detector (TOSOH).

### Flow Cytometry for Cell Uptake

THP-1 dual cells were plated in FBS-containing RPMI media at 100000 cells per well in a 96-well plate at 180 μL and incubated at either 4 °C or 37 °C for 2 hours. The media were diluted to 5 μM in FBS-containing RPMI media for a final volume of 200 μL and incubated at either 4 or 37 °C for 2 h. After incubation, the plates were centrifuged at 500 g for 5 min at 4 °C, decanted, and washed three times with refrigerated 1% BSA-containing PBS. A 20000× dilution of DAPI was used to prepare a DAPI-containing 1% BSA in PBS solution for a final cell suspension at 100 μL per well. Data were collected and analyzed for cell uptake on a CellStream Flow Cytometer (Luminex) equipped with SSC, FFC, 405 nm (DAPI), and 642 nm (Cy5) lasers.

### Confocal Microscopy for Intracellular Colocalization

Primary wild-type cells (kind gift from Marjan Rafat, Vanderbilt University), STING-GFP^34^ (provided by N. Yan, UT Southwestern), and STING*–/–* ^34^ (provided by N. Yan, UT Southwestern) murine embryonic fibroblasts (MEFs) were grown in complete DMEM medium supplemented with 10% (v/v) heat-inactivated fetal bovine serum (HI-FBS; Gibco), 4.5 g/L glucose, 2 mM l-glutamine, 25 mM HEPES, and 100 U mL^–1^ penicillin/100 μg mL^–1^ streptomycin (Gibco). Cells were seeded at 1 × 10^5^ cells per slip on fibronectin-coated coverslips or 2 × 10^5^ cells per dish on fibronectin-coated 35 mm MatTek dishes and incubated overnight. Cells were washed with PBS and dosed with 5 μM drug in complete DMEM (no phenol red, Gibco) for 2 or 4 h. For Lysotracker assays, 75 nM Lysotracker Red DND-99 (Thermo Fisher) was added 1 h before imaging, and cells were washed with complete DMEM (no phenol red, Gibco) immediately before imaging. For ER colocalization assays, 1 μM ER Tracker Red (Thermo Fisher) was added 30 min before imaging, and cells were washed and incubated in complete FluoroBrite DMEM (Gibco) immediately before imaging. Confocal images for Lysotracker analysis were taken over time using a Nikon A1R-HD25 confocal microscope with a motorized stage, Tokai Hit stage-top incubation system with a built-in digital gas mixer for 100% CO_2_ use, equipped with an Apo TIRF 60*x*/1.49 oil immersion lens. Images were acquired using Nikon NIS-Elements software and analyzed using the JACoP ImageJ plugin. Live-cell imaging for WT and STING–/– MEFs with ER Tracker was performed on a Nikon Ti2 inverted light microscope equipped with a Yokogawa CSU-W1 spinning disk head, a Photometrics Prime 95B sCMOS camera, four excitation lasers (488, 568, 647, and 405 nm), and a 100*X*/1.49 NA TIRF oil immersion objective. Cells were maintained in a Tokai Hit stage-top incubator at 37 °C with 5% CO_2_. Images presented in the figures were deconvolved using Nikon Elements software (3D-Deconvolution Richardson-Lucy, 12–20 iterations). Manders’ and Pearson’s coefficients were calculated using the BIOP JACoP ImageJ plugin and Moments auto-threshold after selecting regions of interest in each image.

### Tritosome Assay

Isolated rat lysosomes (tritosomes) and a lysosome/tritosome buffer were purchased from BioIVT. diABZI variants were incubated at 100 μM in sterile water, complete with 10% tritosome and 10% buffer, for 7 h at 37 °C in a total reaction volume of 50 mL. 20 mL samples were taken at 0 and 7 h and frozen at −80 °C until MALDI-MS analysis was performed as described above.

### Instrumentation for LC-MS Analysis

Chromatographic separation and acquisition were performed on a Waters Acquity UPLC system equipped with an Agilent PoroShell 120 EC-C18 column (2.7 μm particle size). The UPLC system was coupled to a Finnigan TSQ Quantum Ultra triple quadrupole mass spectrometer (Thermo Scientific, USA) for mass detection. Data acquisition, spectral analysis, peak integration, and quantification were carried out by using the latest version of the instrument control and data processing software.

### Mass Spectrometric Detection and Optimization Method

LC-MS experiments were performed to optimize the mass spectrometric detection of diABZI-amine. Solutions ranging from 100 ng/mL to 100 μg/mL were prepared in acetonitrile: water (1:1, v/v) by diluting a 1 mg/mL DMSO stock. Analysis was conducted in positive electrospray ionization (ESI) mode for sensitive ESI-MS/MS method development. The protonated molecular ion [M + H]^+^ was observed at *m*/*z* 780.400 and selected as the precursor for MS/MS fragmentation. At a collision energy of 56 eV, this precursor yielded a prominent product ion at *m*/*z* 109, attributed to a characteristic fragment loss (Figure S35). Additional fragments were detected at *m*/*z* 137 and 364, and the proposed fragmentation scheme is presented in Figure S35.

Chromatographic separation was carried out using a Waters Acquity UHPLC system (Waters, USA) interfaced with a Finnigan TSQ-Quantum Ultra triple quadrupole mass spectrometer (Thermo Scientific, USA) equipped with a heated electron ionization (HESI) source and a HyperQuad quadrupole mass analyzer. An Agilent PoroShell 120 EC-C18 column (3 × 50 mm, 2.7 μm) was used for the UHPLC separation. The mobile phases consisted of water containing 0.1% trifluoroacetic acid (TFA) (A) and acetonitrile with 0.1% TFA (B). The gradient program was as follows: 0–1 min, 5% B; 1.1–7 min, linear increase to 95% B; 8 min, hold at 95% B; and 8.1–10 min, re-equilibration at 5% B. The flow rate was maintained at 300 μL/min. Mass spectrometric conditions in positive ESI mode were optimized as follows: ion spray voltage, 4500 V; capillary temperature, 300 °C; sheath gas pressure, 30; ion sweep gas, 4; auxiliary gas, 5; tube lens offset, 109; skimmer offset, −10; collision pressure, 1.6 mTorr; and collision energy, 56 eV (Figure S36). This optimized LC-MS/MS method was subsequently applied for the sensitive detection and quantification of diABZI-amine and its polymeric conjugates in biological samples.

### Extraction and Sample Preparation for LC-MS Analysis

THP1-Dual cells were seeded in 6-well plates at a density of 2 × 10^6^ cells per well in 1 mL of complete media and incubated for 24 h. Cells were treated with diABZI-amine and other test compounds at a final concentration of 60 μM, prepared by adding 1 mL of compound-containing media to each well. Following incubation for the designated time points, media were carefully collected, and aliquots were retained for the Quanti-Luc assay to quantify relative interferon (IFN) production.

Cells were transferred to 2.0 mL microcentrifuge tubes, centrifuged at 1500 rpm for 5 min, and washed with cold PBS. After a second centrifugation under the same conditions, the cell pellets were resuspended in 700 μL of ice-cold 80:20 (v/v) methanol/water (prestored at −80 °C). The suspensions were vortexed and sonicated for 2 min and then stored at −80 °C for at least 2 h to ensure complete lysis and protein precipitation. Samples were thawed on ice or at 4 °C for 15 min, followed by centrifugation at 14000 × g for 5 min at 4 °C. The supernatant was collected, and a second extraction was performed by adding 300 μL of chilled 80:20 methanol/water to the remaining pellet, vortexing briefly, and centrifuging under the same conditions. Combined supernatants were used for HPLC and LC-MS analyses to quantify diABZI-amine content.

### Galectin 8 Reporter Assay

Galectin 8 (Gal8) reporter assays were conducted as previously described, with minor modifications.^51^ Briefly, MDA-MB-231 human breast adenocarcinoma cells expressing a Gal8-YFP fusion protein were seeded at a density of 5000 cells per well in 96-well black-walled, clear-bottom, TC-treated plates (Greiner) and allowed to incubate overnight. Once adhered, cells were treated with formulations of PBS as a control. After 24 h, the medium was discarded and replaced with Fluorobrite DMEM imaging medium (Gibco) supplemented with 1:5000 Hoechst nuclear stain (Thermo Fisher Scientific). Cells were imaged using an ImageXpress Nano Automated Imaging System with a 20× Nikon CFI60 series objective (Molecular Devices), courtesy of the Vanderbilt High-Throughput Screening Facility. Images were then analyzed using MetaXpress software (Molecular Devices), which quantified the number of Gal8-YFP pixels per cell, representing points of endosome disruption. A nonlinear regression curve was fit to the data points, and EC50 values were obtained by using GraphPad Prism 10.2.3 software.

### Immortalized Bone Marrow-Derived Macrophages (iBMDM) Dosing and IFN-β Reporter ELISA

LRRC8A knockout or vector control iBMDMs^39^ (provided by J. Kagan, Harvard Medical School) were cultured in Dulbecco’s Modified Eagle Medium (DMEM) (Gibco) supplemented with 4.5 g/L glucose, 2 mM l-glutamine, 25 mM HEPES, 10% HI-FBS (Gibco), 100 U/mL penicillin (Gibco), and 100 μg/mL streptomycin (Gibco). For dosing, 750000 iBMDM cells were plated in a 12-well plate in 1 mL of media, and cells were allowed to adhere over a 24-h period. After the cells were adherent, treatments in 1 mL of medium were administered at a 20 μM diABZI concentration. Results were collected 24 h after treatment using a mouse IFN-β solid-phase sandwich ELISA kit (Invivogen Cat#42400-1) on cell supernatants following manufacturer’s instructions. Luminescence was quantified using a plate reader (Synergy H1Multi-Mode Microplate Reader; Biotek).

### B16-F10 Melanoma Tumor Model

6–8-week-old C57BL/6 mice (6–8 weeks old) were inoculated with B16-F10 tumors by subcutaneously injecting 1 × 10^6^ cells suspended in 100 μL of PBS into the rear right flank. When tumors were ∼50 mm^3^, the mice were given a total of 3100 μL intravenous injections administered every 3 days containing either PBS, diABZI-NH_2_, or diABZI-PEG20_kDa_ at 0.035 μmol/mouse. Tumor volume and murine weight were measured for the duration of the study. The study end point for maximum tumor volume was 1500 mm^3^.

### MC38-Luc Tumor Model

6–8-week-old C57BL/6 mice were inoculated with MC38-Luc tumors by injecting 1 × 10^6^ cells suspended in 100 μL of PBS into the IP space. Tumors were qualitatively measured using IVIS imaging and randomized on day 6 post-inoculation. Mice were treated on day 7 and day 10 post-inoculation with either PBS, diABZI-ECT, or diABZI-DMA at 0.007 μmol/mouse. Mice were sacrificed on day 14 post-inoculation, and tumor weights were reported.

### IP Retention Study

Seven-week-old C57BL/6 mice were injected (IP) with PBS, diABZI-sCy5, or diABZI-DMA-Cy5 (0.006 μmol/mouse) at 100 μL injection volume and imaged under isoflurane 24 h post-injection using an IVIS Lumina III (PerkinElmer). Fluorescence (radiant efficiency) was measured, and average radiant efficiency values (per cm²) were calculated using the Living Image software (version 4.5).

### Statistical Analysis

Significance for each experiment was determined as indicated in the corresponding figure captions. All analyses were done using GraphPad Prism software, version 9. Plotted values represent experimental means, and error bars represent SD unless otherwise noted in the figure captions. **** *p* < 0.0001, *** *p* < 0.005, **p < 0.01, * *p* < 0.05.

## Supplementary Material







## Notes

Additional details on the chemical synthesis, polymer synthesis, and material characterization, as well as supplemental data and microscopy videos (PDF)
Time-lapse from MEF cells expressing a STING-GFP fusion protein treated with diABZI-ECT for the indicated time (ZIP)
Time-lapse from MEF cells expressing a STING-GFP fusion protein treated with diABZI-DMA for the indicated time (ZIP)
