# Supplementary material for: Macromolecular Diamidobenzimidazole Conjugates Can Activate Stimulator of Interferon Genes
Source: J Am Chem Soc. 2025 Sep 12;147(38):35149–63. doi: 10.1021/jacs.5c13195 (PMC12464991; doi:10.1021/jacs.5c13195)
Supplement: Supplementary file 1 [file ja5c13195_si_001.pdf]

Macromolecular Diamidobenzimidazole Conjugates Can Activate Stimulator of Interferon Genes

Karan Arora[1,^], Taylor L. Sheehy[2,^], Jacob A. Schulman[2,^], Jack R. Loken[3], Zachary Lehmann[4], Blaise R. Kimmel[1], Caitlin McAtee[4], Vijaya Bharti[1], Payton T. Stone[1], Alissa M. Weaver[4], Matthew Tyska[4], Rakesh Kumar Pathak[1,5], John T. Wilson[1-3,6-10*]

[1]Department of Chemical and Biomolecular Engineering, Vanderbilt University, Nashville, TN, 37212, USA
[2]Department of Biomedical Engineering, Vanderbilt University, Nashville, TN, 37240, USA
[3]Interdisciplinary Materials Science Program, Vanderbilt University, Nashville, TN, 37240, USA
[4]Department of Cell and Developmental Biology, Vanderbilt University, Nashville, TN, 37240, USA
[5]Department of Chemical Sciences, Indian Institutes of Science Education and Research (IISER Berhampur), Berhampur, Odisha, 760010, India
[6]Vanderbilt Institute of Chemical Biology, Vanderbilt University, Nashville, TN, 37240, USA
[7]Vanderbilt Institute of Nanoscale Science and Engineering, Vanderbilt University, Nashville, TN, 37212, USA
[8]Vanderbilt Institute for Infection, Immunology, and Inflammation, Vanderbilt University, Nashville, TN, 37232, USA
[9]Vanderbilt Center for Immunobiology, Vanderbilt University Medical Center, Nashville, TN, 37232, USA
[10]Vanderbilt Ingram Cancer Center, Nashville, TN, 37232, USA

^equally contributing authors

***To whom correspondence should be addressed:**
John T. Wilson, Ph.D.
2400 Highland Avenue
107 Olin Hall
Nashville, TN 37212
Phone: +1-615-322-6406
e-mail: john.t.wilson@vanderbilt.edu

Supporting Information
(41 Pages)

Part A. General Considerations
Part B. Synthesis — Schemes S1-S8
Part C. Supplemental Data — Figures S1-S8
Part E. ^{1}H-NMR spectra for new compounds. — Figures S9-S23
Part F. ^{13}C-NMR spectra for new compounds. — Figures S24-S27
Part G. HRMS spectra for new compounds. — Figures S28-S32
Part H. GPC Analysis for polymers. — Figures S33-S34

Part I. MS/MS Fragmentation. Figure S35
Part J. Microscopy Videos Videos S1-S2
Part K. References

Part A: General Considerations

All reagents were purchased from commercial suppliers and used as received. ^{1}H-NMR, ^{13}C-NMR spectra were recorded on a Bruker – 400 MHz or 600 MHz Spectrometer. Mass spectra were recorded on a Thermo-Fisher Scientific LTQ-Orbitrap XL Mass Spectrometer. All reactions were performed under ambient atmosphere unless otherwise noted. Anaerobic reactions were performed by purging the reaction solutions with argon or nitrogen. MeO-PEG$_{5KDa}$-NHS ester, MeO-PEG$_{20Kda}$-NHS ester, MeO-PEG$_{5Kda}$-SH and MeO-PEG$_{20Kda}$-SH were purchased from JenKem Technology USA. Compounds **1**[1], **4**[2], **8**[3], **10**[4], and **15**[5] were synthesized as per the literature protocol.

Part B: Synthesis

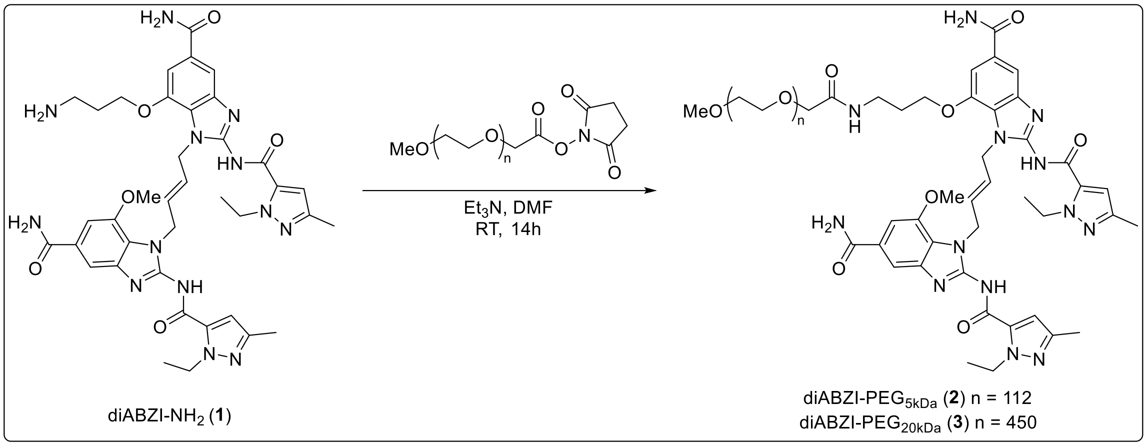

Scheme S1: Synthesis of diABZI-PEG$_{5kDa}$ (**2**) and diABZI-PEG$_{20kDa}$ (**3**).

diABZI-PEG$_{5kDa}$ (2): A solution of MeO-PEG$_{5KDa}$-NHS ester (300 mg, 60 µmol, 1 eq.) in 4 mL 1:1 DCM:DMF was added dropwise to a solution of diABZI-NH$_2$ (**1**) (81 mg, 72 µmol, 1.2 eq.) and Et$_3$N (42 µL, 300 µmol, 5 eq.) in 2 mL DMF and was stirred overnight. The desired compound (**2**) was purified by dialysis (3 kDa MWCO) against (1:1) DCM:MeOH (2x), acetone (2x), and then deionized water (2x). The desired purified product (180 mg, 31.2 µmol, 52%) was lyophilized to obtain a white, fluffy powder and characterized by ^{1}H-NMR. ^{1}H-NMR (400 MHz, DMSO) δ 7.95 (broad s, 2H), 7.94 (broad s, 3H), 7.73 (t, 1H), 7.64 (s, 2H), 7.31 – 7.28 (m, 5H), 6.5 (d, 2H), 5.87 – 5.82 (m, 2H), 4.96 – 4.89 (m, 4H), 4.53 – 4.50 (m, 4H), 3.97 (t, *J* = 6.1 Hz, 2H), 3.81 (s, 3H, terminal methoxy of PEG), 3.72 (s, 3H), 3.51 (broad s, 462H, -CH$_2$-CH$_2$- of PEGMA), 2.10 (s, 3H), 2.09 (s, 3H), 1.70 (p, *J* = 6.1 Hz, 2H), 1.28 – 1.24 (m, 6H).

diABZI-PEG$_{20kDa}$ (3): A solution of MeO-PEG$_{20KDa}$-NHS ester (500 mg, 25 µmol, 1 eq.) in 5 mL 1:1 DCM:DMF was added dropwise to a solution of diABZI-NH$_2$ (**1**) (34 mg, 30 µmol, 1.2 eq.) and Et$_3$N (17.5 µL, 125 µmol, 5 eq.) in 1.5 mL DMF and was stirred overnight. The desired compound (**3**) was purified by dialysis (3 kDa MWCO) against (1:1) DCM:MeOH (2x), acetone (2x), and then deionized water (2x). The desired purified product (393 mg, 19 µmol, 76%) was lyophilized to obtain a white, fluffy powder and characterized by ^{1}H-NMR.

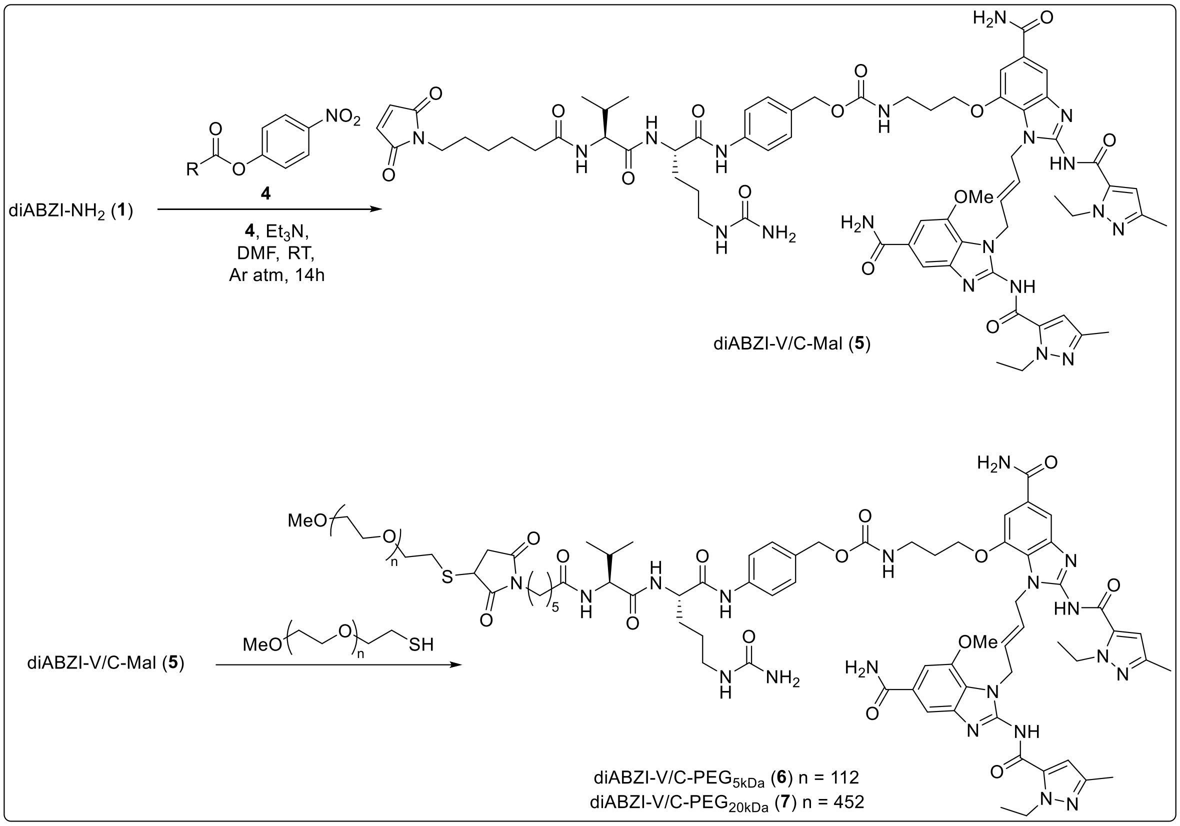

Scheme S2: Synthesis of diABZI-V/C-Mal (**5**), diABZI-V/C-PEG$_{5KDa}$ (**6**), and diABZI-V/C-PEG$_{20KDa}$ (**7**)

4-((*S*)-2-((*S*)-2-(6-(2,5-dioxo-2,5-dihydro-1*H*-pyrrol-1-yl)hexanamido)-3-methylbutanamido)-5-ureidopentanamido)benzyl (3-((5-carbamoyl-1-((*E*)-4-(5-carbamoyl-2-(1-ethyl-3-methyl-1*H*-pyrazole-5-carboxamido)-7-methoxy-1*H*-benzo[*d*]imidazol-1-yl)but-2-en-1-yl)-2-(1-ethyl-3-methyl-1*H*-pyrazole-5-carboxamido)-1*H*-benzo[*d*]imidazol-7-yl)oxy)propyl)carbamate, diABZI-V/C-Mal (5). A solution of 4-((*S*)-2-((*S*)-2-(6-(2,5-dioxo-2,5-dihydro-1*H*-pyrrol-1-yl)hexanamido)-3-methylbutanamido)-5-ureidopentanamido)benzyl (4-nitrophenyl) carbonate (**4**) (253 mg, 0.34 mmol, 1.1 eq.) in 3 mL DMF was added dropwise to a solution of diABZI-NH$_2$ (**1**) (350 mg, 0.3 mmol, 1 eq.) and Hunig's base (0.27 mL, 1.56 mmol, 5 eq.) in 5 mL DMF under inert atmosphere and was stirred overnight at room temperature. Then, diethyl ether added to precipitate out the crude solid desired product. The solid was filtered over a Büchner funnel and resuspended in 5 mL 1:1 DCM:MeOH and stirred for 2 hours. The suspension was filtered over a Büchner funnel to obtain the pure desired product as a light pink solid (**4**) (413 mg, 0.29 mmol, 96%). ^{1}H-NMR (400 MHz, DMSO) δ 9.95 (s, 1H), 8.06 (d, *J* = 7.5 Hz, 1H), 7.95 (broad s, 2H), 7.79 (d, *J* = 8.5 Hz, 1H), 7.63 (d, *J* = 6.7 Hz, 2H), 7.56 (d, *J* = 8.5 Hz, 2H), 7.34 – 7.23 (m, 7H), 6.99 (s, 2H), 6.5 (d, *J* = 5.6 Hz, 2H), 5.96 (t, *J* = 5.0 Hz, 1H), 5.88 – 5.79 (m, 2H), 5.40 (s, 2H), 4.93 – 4.87 (m, 6H), 4.53 – 4.49 (m, 4H), 4.38 – 4.35 (m, 1H), 4.19 – 4.16 (m, 1H), 3.98 (t, *J* = 5.0 Hz, 2H), 3.71 (s, 3H), 3.40 – 3.34 (m, 2H), 3.08 – 2.90 (m, 4H), 2.20 – 1.92 (m, 9H), 1.73 – 1.54 (m, 4H), 1.52 – 1.14 (m, 16H), 0.84 (d, *J* = 6.7 Hz, 3H), 0.80 (d, *J* = 6.7 Hz, 3H). ^{13}C NMR (151 MHz, DMSO) δ 172.7, 171.8, 171.5, 171.0, 168.1, 167.3, 159.4, 156.6,

145.5, 145.3, 140.4, 139.0, 134.9, 132.2, 130.5, 129.1, 119.4, 109.7, 65.5, 58.0, 56.4, 53.6, 46.0, 37.5, 35.4, 30.8, 29.8, 28.2, 27.3, 26.2, 25.4, 19.7, 18.7, 16.6, 13.6. HRMS (ESI-MS) calculated for $C_{67}H_{83}N_{19}O_{14}$ $[M+H]^+$: 1378.6440, found 1378.6415.

diABZI-V/C-PEG$_{5KDa}$ (6): A solution of N-methyl morpholine (NMM, 9 μL, 79.1 μmol, 4.0 eq.) in 0.5 mL DMF was added dropwise to a solution of diABZI-V/C-Mal (**5**) (30 mg, 21.7 μmol, 1.1 eq.) and mPEG$_{5kDa}$-SH (100 mg, 19.8 μmol, 1.0 eq.) in 2 mL DMF and was stirred overnight. The desired product was purified by dialysis (3 kDa MWCO) against (1:1) DCM:MeOH (2x), acetone (2x), and then deionized water (2x) and subsequently lyophilized to obtain a white, fluffy powder (84 mg, 13.2 μmol, 66%). ^{1}H-NMR (400 MHz, DMSO) δ 9.96 (s, 1H), 8.08 (s, 1H), 7.95 (broad s, 2H), 7.79 (d, *J* = 8.5 Hz, 1H), 7.64 (d, *J* = 6.7 Hz, 2H), 7.57 (d, *J* = 8.5 Hz, 2H), 7.34 – 7.23 (m, 6H), 6.5 (s, 1H), 5.96 (t, *J* = 5.0 Hz, 1H), 5.89 – 5.79 (m, 2H), 5.40 (s, 2H), 4.94 – 4.88 (m, 6H), 4.53 – 4.49 (m, 4H), 4.39 – 4.35 (m, 1H), 4.20 – 4.18 (m, 1H), 3.98 (t, *J* = 5.0 Hz, 2H), 3.71 (s, 3H), 3.50 (broad s, 395H, -CH_2-CH_2- of PEGMA), 3.19 – 2.79 (m, 6H), 2.20 – 1.92 (m, 9H), 1.73 – 1.54 (m, 4H), 1.52 – 1.14 (m, 14H), 0.85 (d, *J* = 6.7 Hz, 3H), 0.82 (d, *J* = 6.7 Hz, 3H).

diABZI-V/C-PEG$_{20kDa}$ (7): A solution of N-methyl morpholine (NMM, 9 μL, 79.1 μmol, 4.0 eq.) in 0.5 mL DMF was added dropwise to a solution of diABZI-V/C-Mal (**5**) (30 mg, 21.7 μmol, 1.1 eq.) and mPEG(20k)-SH (396 mg, 19.8 μmol, 1.0 eq.) in DMF (2.0 mL) and was stirred overnight. The desired product was purified by dialysis (3 kDa MWCO) against (1:1) DCM:MeOH (2x), acetone (2x), and then deionized water (2x) and subsequently lyophilized to obtain white fluffy powder (271 mg, 12.7 μmol, 64%).

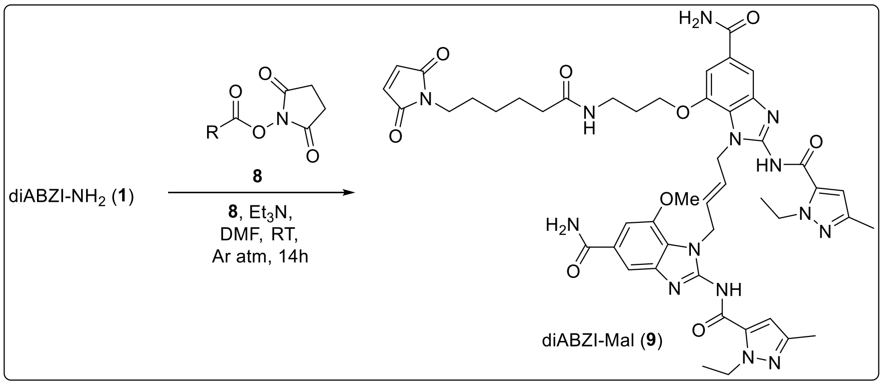

Scheme S3: Synthesis of diABZI-Mal (**9**).

(*E*)-1-(4-(5-carbamoyl-2-(1-ethyl-3-methyl-1*H*-pyrazole-5-carboxamido)-7-methoxy-1*H*-benzo[*d*]imidazol-1-yl)but-2-en-1-yl)-7-(3-(6-(2,5-dioxo-2,5-dihydro-1*H*-pyrrol-1-yl)hexanamido)propoxy)-2-(1-ethyl-3-methyl-1*H*-pyrazole-5-carboxamido)-1*H*-benzo[*d*]imidazole-5-carboxamide, diABZI-Mal (9). A solution of 2,5-dioxopyrrolidin-1-yl 6-(2,5-dioxo-2,5-dihydro-1*H*-pyrrol-1-yl)hexanoate (**8**[3]) (66 mg, 0.2 mmol, 1.2 eq.) in 2 mL DMF was added dropwise to a stirred solution of diABZI-NH_2 (**1**) (200 mg, 0.18 mmol, 1 eq.) and Hunig's base (0.12 mL, 0.89 mmol, 5 eq.) in 3 mL DMF under inert atmosphere and was stirred overnight at room temperature. Then, diethyl ether was added to precipitate out the crude solid desired product. The crude solid was purified over silica gel chromatography (DCM:MeOH 0-20%) to

obtain the diABZI-Mal (**9**) as an off white solid (80 mg, 0.08 mmol, 46%). ^{1}H-NMR (400 MHz, DMSO) δ 7.95 (broad s, 2H), 7.76 (t, *J* = 5.5 Hz, 1H), 7.63 (s, 2H), 7.32 (braod s, 2H), 7.29 (d, *J* = 6.2 Hz, 2H), 6.97 (s, 2H), 6.5 (d, *J* = 7.6 Hz, 2H), 5.89 – 5.77 (m, 2H), 4.95 – 4.88 (m, 4H), 4.54 – 4.48 (m, 4H), 3.96 (t, *J* = 6.1 Hz, 2H), 3.71 (s, 3H), 3.09 (dt, *J* = 6.2, 5.9 Hz, 2H), 2.10 (s, 3H), 2.09 (s, 3H), 1.98 (t, *J* = 7.2 Hz, 2H), 1.67 (p, *J* = 6.1 Hz, 2H), 1.47 – 1.40 (m, 4H), 1.25 (dt, J = 7.1, 3.2 Hz, 6H), 1.18 – 1.10 (m, 2H). ^{13}C NMR (151 MHz, DMSO) δ 172.4, 171.5, 168.1, 167.3, 152.5, 145.5, 145.3, 144.7, 140.4, 140.3, 134.9, 130.5, 130.5, 128.7, 128.2, 120.1, 109.7, 56.4, 46.0, 37.4, 35.6, 29.2, 28.2, 26.3, 25.2, 16.6, 13.6. HRMS (ESI-MS) Calculated for $C_{48}H_{56}N_{14}O_9$ $[M+H]^+$: 973.4427, found 973.4417.

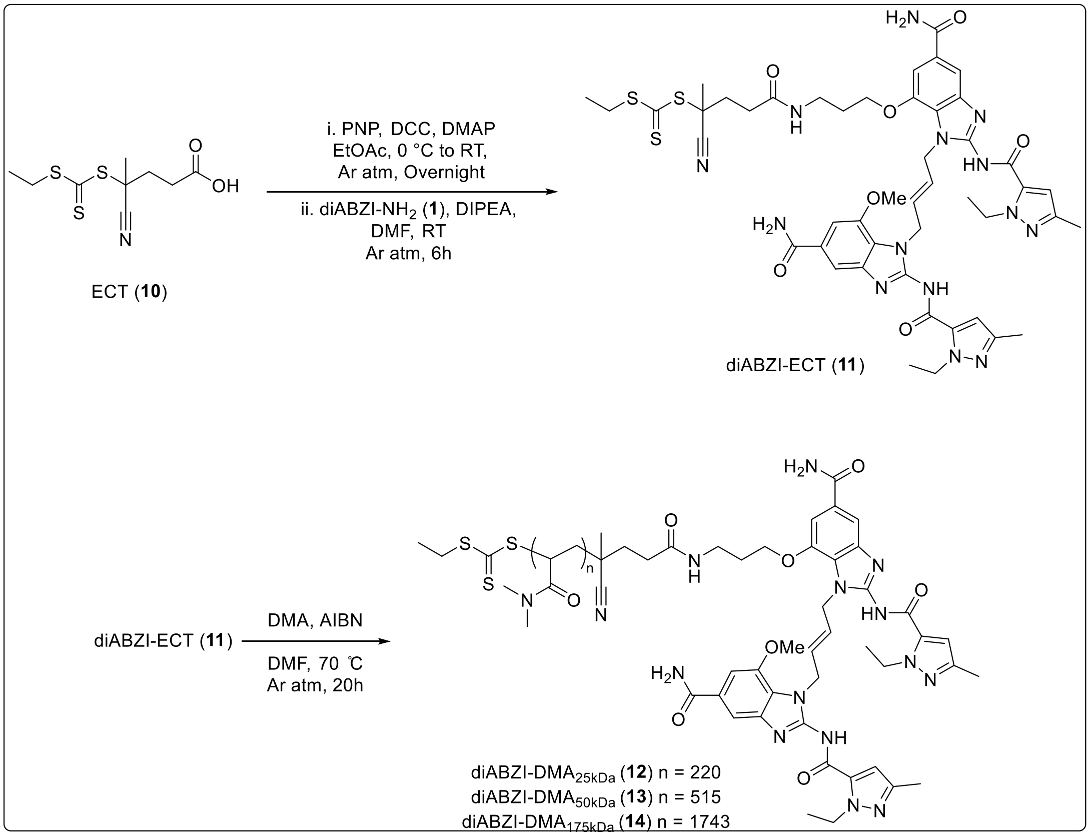

Scheme S4: Synthesis of diABZI-ECT (**11**), diABZI-DMA$_{25kDa}$ (**12**), diABZI-DMA$_{50kDa}$ (**13**), and diABZI-DMA$_{175kDa}$ (**14**).

(*E*)-5-((3-((5-carbamoyl-1-(4-(5-carbamoyl-2-(1-ethyl-3-methyl-1*H*-pyrazole-5-carboxamido)-7-methoxy-1*H*-benzo[*d*]imidazol-1-yl)but-2-en-1-yl)-2-(1-ethyl-3-methyl-1*H*-pyrazole-5-carboxamido)-1*H*-benzo[*d*]imidazol-7-yl)oxy)propyl)amino)-2-cyano-5-oxopentan-2-yl ethyl carbonotrithioate, diABZI-ECT (11). A solution of 4-cyano-4-(((ethylthio)carbonothioyl)thio)pentanoic acid (**10**[4]) (100 mg, 0.38 mmol, 1 eq.), *p*-nitrophenol (53 mg, 0.38 mmol, 1 eq) and DMAP (5 mg, 0.038 mmol, 0.1 eq.) in 4 mL EtOAc was maintained at 0 °C under argon atmosphere. A solution of DCC (82 mg, 0.40 mmol, 1.05 eq.) in 4 mL EtOAc was added dropwise. The reaction mixture was allowed to warm to room temperature and stirred overnight for 18 hours. After consumption of the starting material, as judged by TLC analysis, the

reaction mixture was filtered through celite. The solvent was evaporated *in vacuo* to obtain the activated ester as a crude product. The crude product was analyzed by 1H NMR spectroscopy and was used without further purification. ^{1}H-NMR (400 MHz, Chloroform-*d*) δ 8.28 (d, *J* = 9.2 Hz, 2H), 7.31 (d, *J* = 9.2 Hz, 2H), 3.36 (q, *J* = 7.4 Hz, 2H), 2.95 (t, *J* = 7.9 Hz, 2H), 2.70 – 2.46 (m, 2H), 1.95 (s, 3H), 1.37 (t, *J* = 7.4 Hz, 3H).

A solution of diABZI-NH_2 (**1**) (300 mg, 0.27 mmol, 1 eq) and Hunig's base (0.23 mL, 1.35 mmol, 5 eq) in 5 mL DMF was stirred under argon atmosphere at room temperature. A solution of crude *p*-nitrophenol ester (144 mg, 0.37 mmol, 1.4 eq) in 4 mL DMF was added dropwise to the reaction mixture and was stirred for 6 hours. The solvent was evaporated *in vacuo* to obtain the crude desired product. The crude solid was purified over silica gel chromatography (DCM:MeOH 0-25%) to obtain diABZI-ECT (**11**) as a light yellow solid (200 mg, 0.19 mmol, 73%). ^{1}H-NMR (400 MHz, DMSO) δ 8.04 (t, *J* = 5.2 Hz, 1H), 7.98 (broad s, 2H), 7.56 (broad s, 2H), 7.35 (broad s, 2H), 7.31 (d, *J* = 5.9 Hz, 2H), 6.56 (d, *J* = 8.5 Hz, 2H), 4.58 – 4.52 (m, 4H), 4.38 – 4.34 (m, 4H), 4.10 (t, *J* = 5.90 Hz, 2H), 3.85 (s, 3H), 3.19 – 3.14 (m, 2H), 2.35 – 2.20 (m, 4H), 2.09 (s, 6H), 1.86 (broad s, 6H), 1.80 (s, 3H), 1.29 (t, *J* = 7.0 Hz, 6H), 1.25 (t, *J* = 7.3 Hz, 3H). HRMS (ESI-MS) Calculated for $C_{47}H_{56}N_{14}O_7S_3$ $[M+H]^+$: 1025.3691, found 1025.3685.

General method of RAFT Polymerization: Reversible addition-fragmentation chain transfer (RAFT) polymerization was used to synthesize three polymer variants with distinct molar masses (25 kDa, 50 kDa, 175 kDa). For synthesis N'N'-dimethylacrylamide (DMA) monomer was filtered over activated alumina and allowed to react under inert atmosphere in DMF (30 wt% monomer) at 70 ºC for 24 h in an oil bath. The initial monomer ($[M]_o$) to diABZI-ECT (**11**) ($[CTA]_o$) to initiator ($[I]_o$) mole ratio was n:1:0.2. The resultant desired polymers diABZI-DMA_{XkDa} were isolated by dialysis (3 kDa MWCO) against pure acetone (2x) and then pure deionized water (2x). Following dialysis, the purified compound was frozen at -80 ºC for 8 hours and then lyophilized for 3 days.

diABZI-DMA_{25kDa} (12): The initial monomer ($[M]_o$) to diABZI-ECT (**11**) ($[CTA]_o$) to initiator ($[I]_o$) ratio was 242:1:0.2. The desired polymer (**12**) was obtained after dialysis as white solid (255 mg, 84%) which was further characterized by ^{1}H NMR. ^{1}H-NMR (400 MHz, DMSO) δ 3.06 – 2.64 (dimethyl group PDMA), 2.62 – 2.11 (-CH- backbone of PDMA), 1.67 – 1.00 (-CH_2- backbone of PDMA),

diABZI-DMA_{50kDa} (13): The initial monomer ($[M]_o$) to diABZI-ECT (**11**) ($[CTA]_o$) to initiator ($[I]_o$) ratio was 525:1:0.2. The desired polymer (**13**) was obtained after dialysis as white solid (365 mg, 72%) which was further characterized by ^{1}H-NMR.

diABZI-DMA_{175kDa} (14): The initial monomer ($[M]_o$) to diABZI-ECT (**11**) ($[CTA]_o$) to initiator ($[I]_o$) ratio was 1755:1:0.2. The desired polymer (**14**) was obtained after dialysis as white solid (663 mg, 65%) which was further characterized by ^{1}H-NMR.

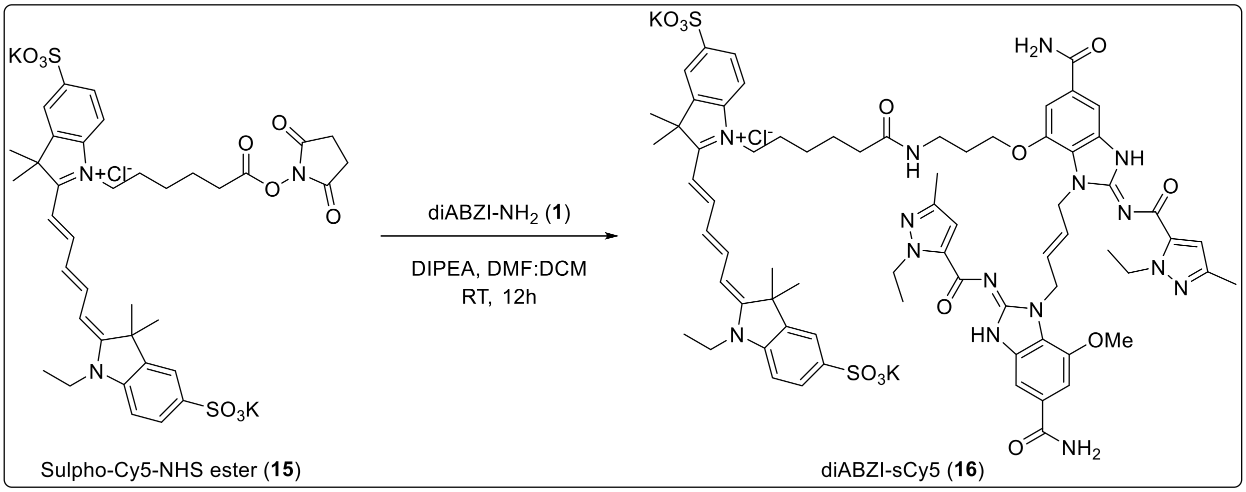

Scheme S5: Synthesis of diABZI-sCY5 (**16**)

Potassium 1-(6-((3-(((*E*)-6-carbamoyl-3-((*E*)-4-((*E*)-5-carbamoyl-2-((1-ethyl-3-methyl-1*H*-pyrazole-5-carbonyl)imino)-7-methoxy-2,3-dihydro-1*H*-benzo[*d*]imidazol-1-yl)but-2-en-1-yl)-2-((1-ethyl-3-methyl-1*H*-pyrazole-5-carbonyl)imino)-2,3-dihydro-1*H*-benzo[*d*]imidazol-4-yl)oxy)propyl)amino)-6-oxohexyl)-2-((1*E*,3*E*)-5-((*E*)-1-ethyl-3,3-dimethyl-5-sulfonatoindolin-2-ylidene)penta-1,3-dien-1-yl)-3,3-dimethyl-3*H*-indol-1-ium-5-sulfonate chloride, diABZI-sCy5 (16). A solution of activated NHS ester compound (**15**[5]) in 1 mL DMF was added dropwise to a stirred solution of diABZI-NH_2 (**1**) (18 mg, 16 µmol, 1.0 eq) and Hunig's base (8.4 µL, 48 µmol, 3 eq) in 1.5 mL DMF. The reaction mixture was stirred overnight and precipitated by adding diethyl ether to obtain the desired product diABZI-sCy5 (**16**) as a blue solid (17.2 mg, 11.2 µmol, 70%). ^{1}H NMR (400 MHz, DMSO) δ 8.33 (t, *J* = 13.0 Hz, 2H), 7.99 (broad s, 2H), 7.82 – 7.79 (m, 3H), 7.66 – 7.61 (m, 4H), 7.35 – 7.28 (m, 8H), 6.61 – 6.44 (m, 3H), 6.35 – 6.21 (m, 2H), 5.88 – 5.70 (m, 2H), 5.00 – 4.83 (m, 4H), 4.52 – 4.45 (m, 4H), 4.14 – 3.93 (m, 6H), 3.72 (s, 3H), 3.11 – 3.05 (m, 2H), 2.09 (s, 3H), 2.08 (s, 3H), 2.00 (t, *J* = 7.4 Hz, 2H), 1.69 – 1.65 (m, 13H), 1.51 – 1.46 (m, 2H), 1.29 – 1.20 (m, 14H). HRMS (ESI-MS) Calculated for $C_{71}H_{84}N_{15}O_{13}S_2$ $[M+H]^{2+}/2$ = 709.7941, found 709.7946.

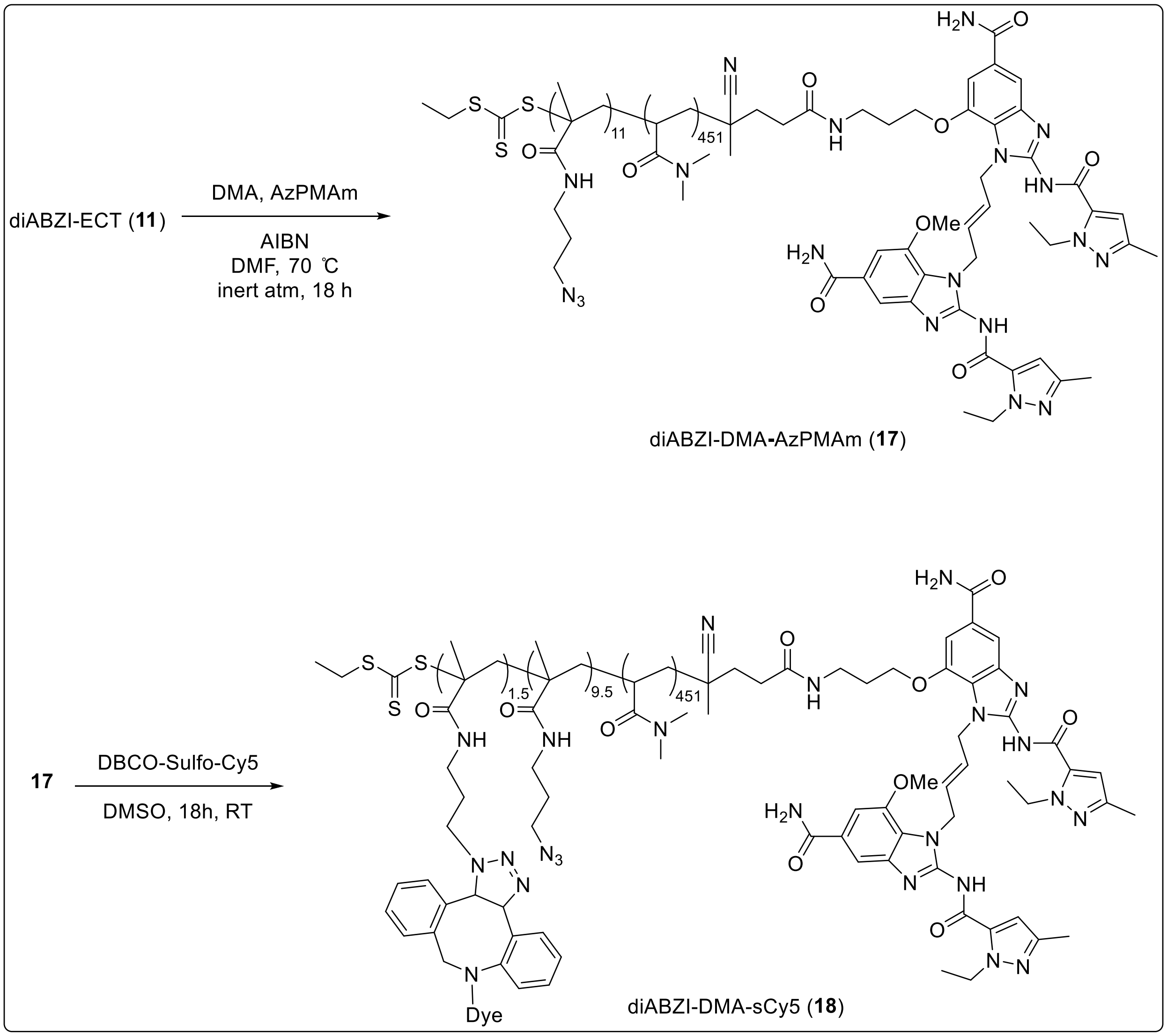

Scheme S6: Synthesis of diABZI-DMA-co-AzPMAm (**17**) and diABZI-DMA-sCy5 (**18**)

diABZI-DMA-sCy5 (18): RAFT polymerization was used to synthesize diABZI-DMA-co-AzPMAm (**17**). For synthesis, DMA was filtered over activated alumina, combined with a solution of diABZI-ECT, AIBN, and AzPMAm, and allowed to react under inert atmosphere in DMF (30 wt% monomer) at 70 ºC for 24 h in an oil bath. The initial DMA to AzPMAm to diABZI-ECT to AIBN mole ratio was 496:11:1:0.2. The resultant desired polymer, diABZI-DMA-coAzPMAm, was isolated by dialysis against pure acetone (2x) and then pure deionized water (2x). Following dialysis, the purified compound was frozen at -80 ºC for 8 h and then lyophilized for 72 hours (494 mg, 91%), which was further characterized by ^{1}H-NMR and GPC in HPLC-grade DMF with 0.01 M LiBr via Light Scattering (TOSOH). GPC analysis showed a monodisperse polymer (PDI = 1.10) at a 69.0 kDa molecular weight.

Copper-free click chemistry was utilized to conjugate DBCO-sCy5 to diABZI-DMA-co-AzPMAm (**17**), forming diABZI-DMA-sCy5 (**18**). diABZI-DMA-co-AzPMAm (**17**) and DBCO-Sulfo-Cy5 were

dissolved separately at 20 mg/mL in DMSO. A molar excess of 2.5:1 DBCO-Sulfo-Cy5 to polymer was utilized for the reaction. Briefly, ~63 µL of DBCO-sCy5 solution was added to 1200 µL of diABZI-DMA-co-AzPMAm (**17**) solution. The reaction was continuously stirred at room temperature in darkness for 24 hours. diABZI-DMA-sCy5 (**18**) was purified via dialysis (3 kDa MWCO) against pure acetone (2x) and then pure deionized water (2×). diABZI-DMA-sCy5 (**18**) was then lyophilized for 72 hours and stored at -20 ºC until use (~15 mg, ~53%). sCy5 concentration was determined using absorbance at 650 nm and diABZI concentration was determined at 325 nm using the NanoDrop UV-Vis Spectrophotometer (Thermo).

Part C: Supplementary Data

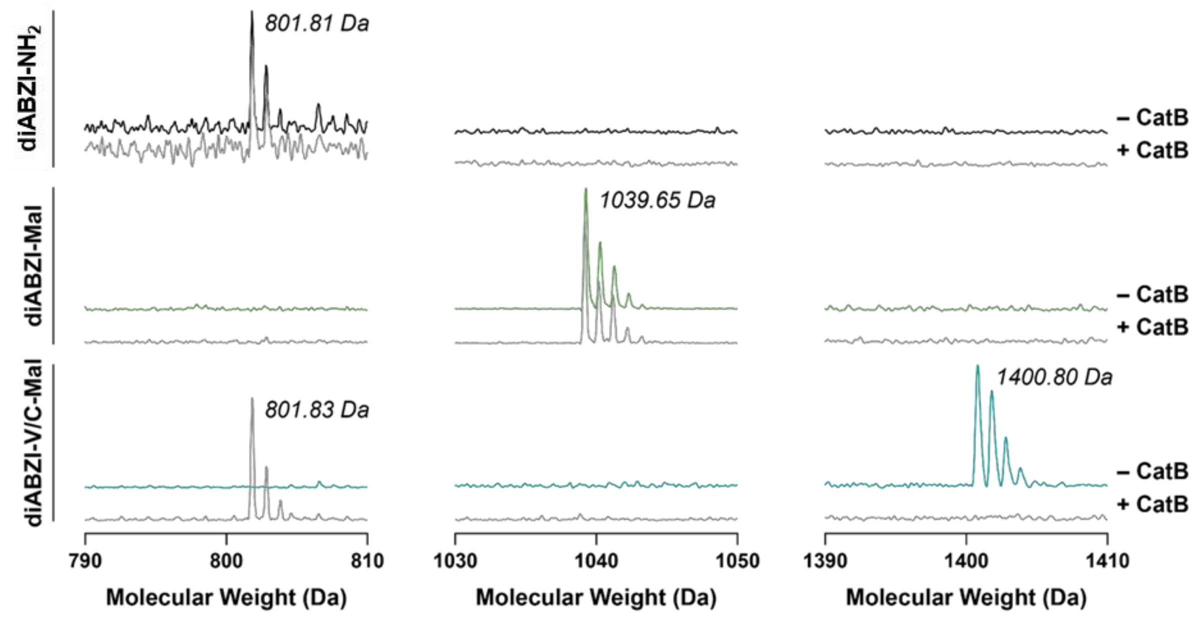

Figure S1. Analysis of cathepsin B-mediated linker cleavage of diABZI-V/C-Mal with diABZI-Mal and diABZI-NH_2 as controls. Molecules were incubated at 50 μM diABZI in a solution of cathepsin B at 37 ºC for 48 hours and MALDI-MS used to detect starting materials and expected cleavage products.

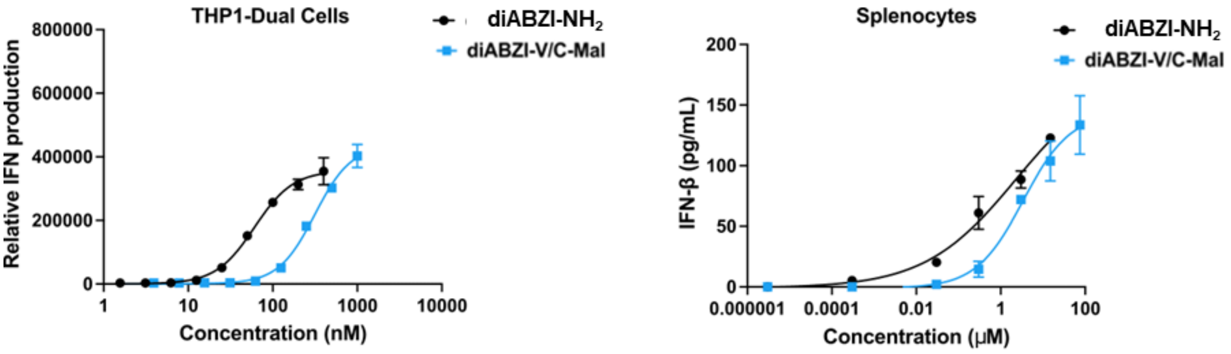

Agonist	EC_{50} (nM)
diABZI-NH_2	61 ± 13
diABZI-V/C-Mal	314 ± 67

Agonist	EC_{50} (μM)
diABZI-NH_2	~2.4
diABZI-V/C-Mal	3.6 ± 2.0

Figure S2. STING activation assays comparing the activity of diABZI-NH_2 and diABZI-V/C-Mal in THP-1 Duals (left) and splenocytes (right) (n = 3).

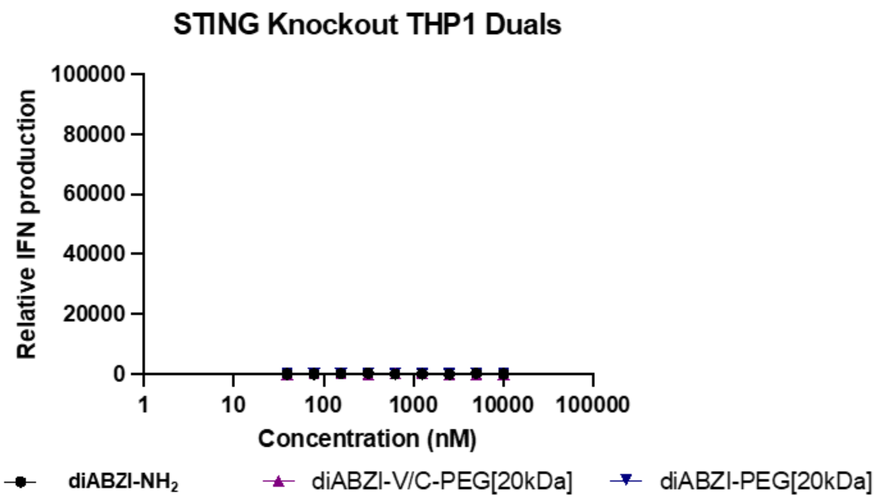

Figure S3: Dose-response curve for relative IFN-I production by STING knockout THP1-Dual reporter cells (n = 3).

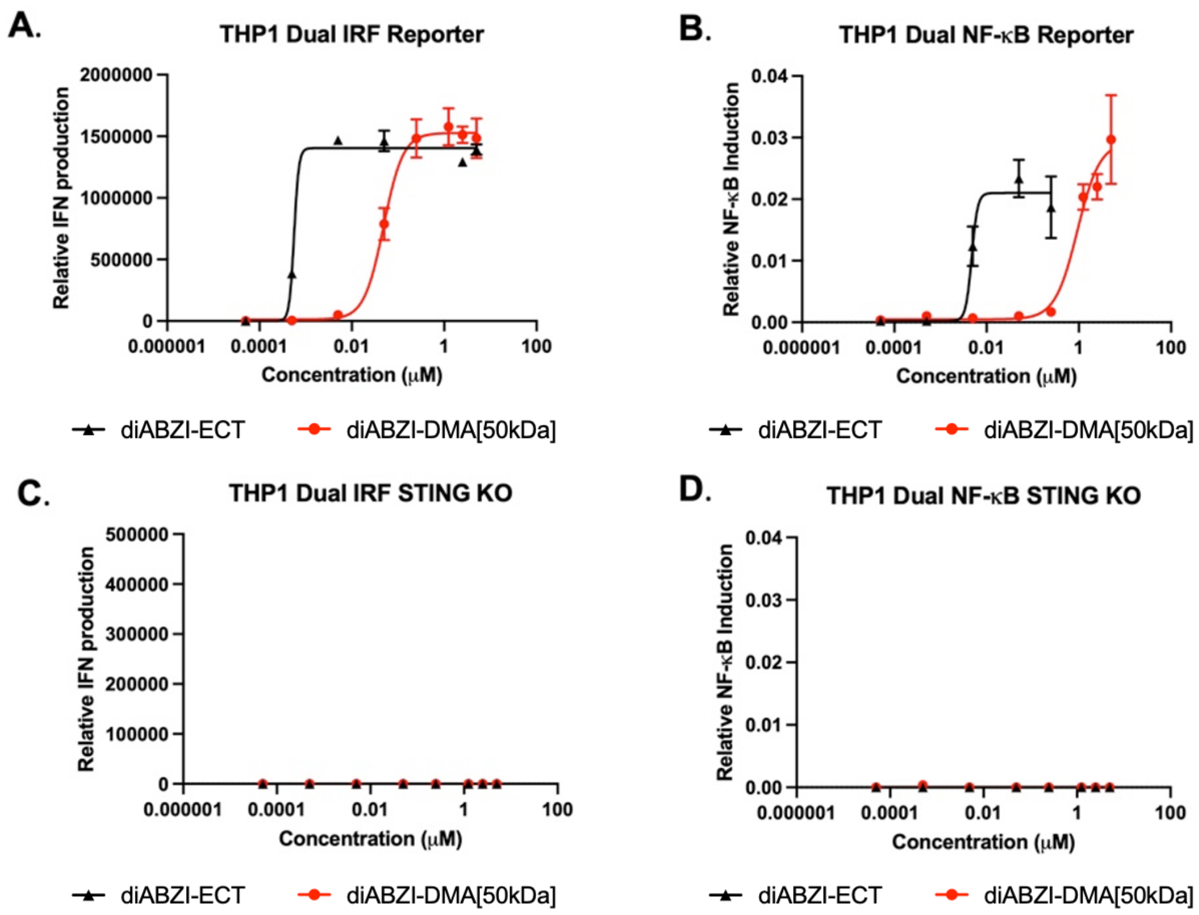

Figure S4: STING activation assays comparing the activity of diABZI-ECT and diABZI-DMA$_{50kDa}$. **(A)** Dose-response curves for relative IFN-I production by THP1-Dual reporter cells treated with diABZI-ECT and diABZI-DMA$_{50kDa}$. **(B)** Dose-response curves for relative NF-κB production by

THP1-Dual reporter cells treated with diABZI-ECT and diABZI-DMA$_{50kDa}$. **(C)** Dose-response curve for relative IFN-I production by STING knockout THP1-Dual reporter cells. **(D)** Dose-response curve for relative NF-κB production by STING knockout THP1-Dual reporter cells. Dose-response curves were fit to a variable slope (four parameter) non-linear regression to estimate EC50 values (n = 3).

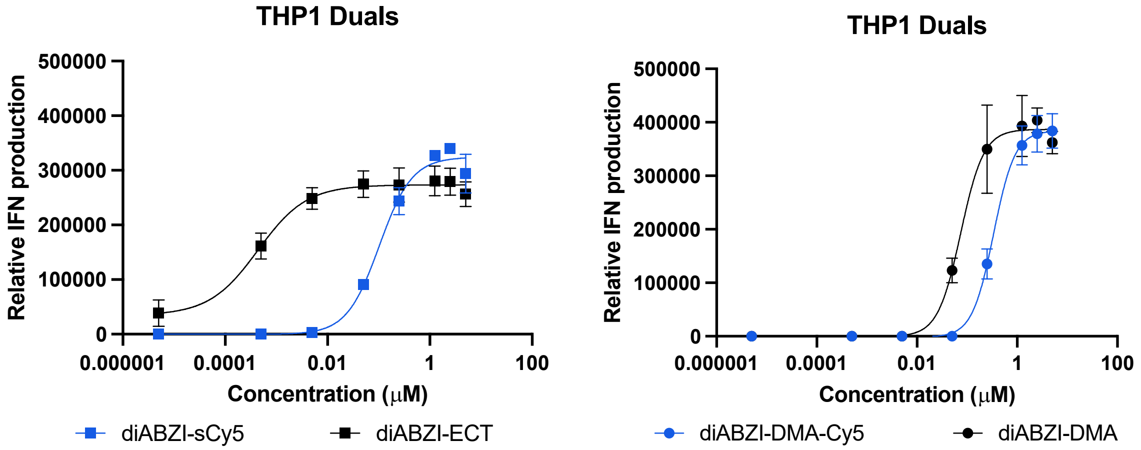

Figure S5: Dose-response curves for relative IFN-I production by THP1-Dual reporter cells treated with diABZI-ECT, diABZI-sCy5, diABZI-DMA-Cy5, and diABZI-DMA. Dose-response curves were fit to a variable slope (four parameter) non-linear regression to estimate EC_{50} values (n = 3).

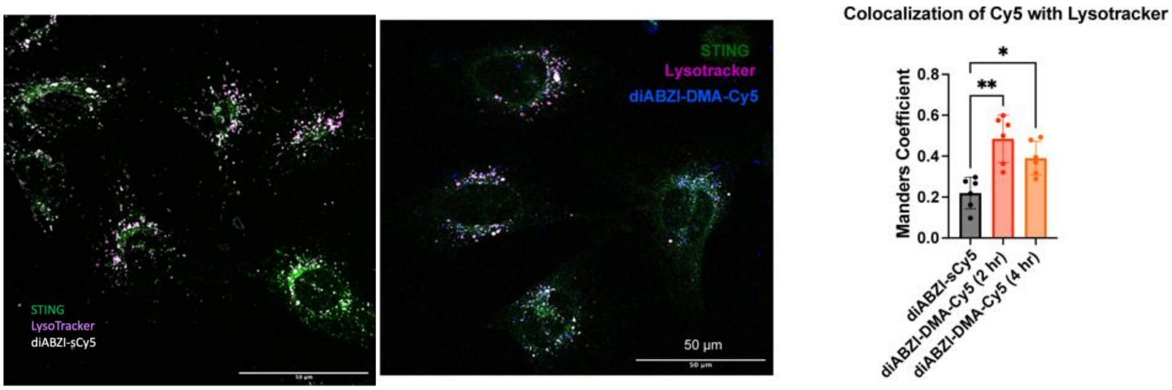

Figure S6: Representative images of lysotracker assay for diABZI-sCy5 (left) and diABZI-DMA (middle) at 2 hours. Representative confocal image of STING-GFP MEFs treated with diABZI-sCy5 (left) and diABZI-DMA-Cy5 (middle) for 2 hours and stained with Lysotracker Red. Quantification of colocalization between labeled diABZI variants and Lysotracker Red (right) described by Manders coefficient (n = 6).

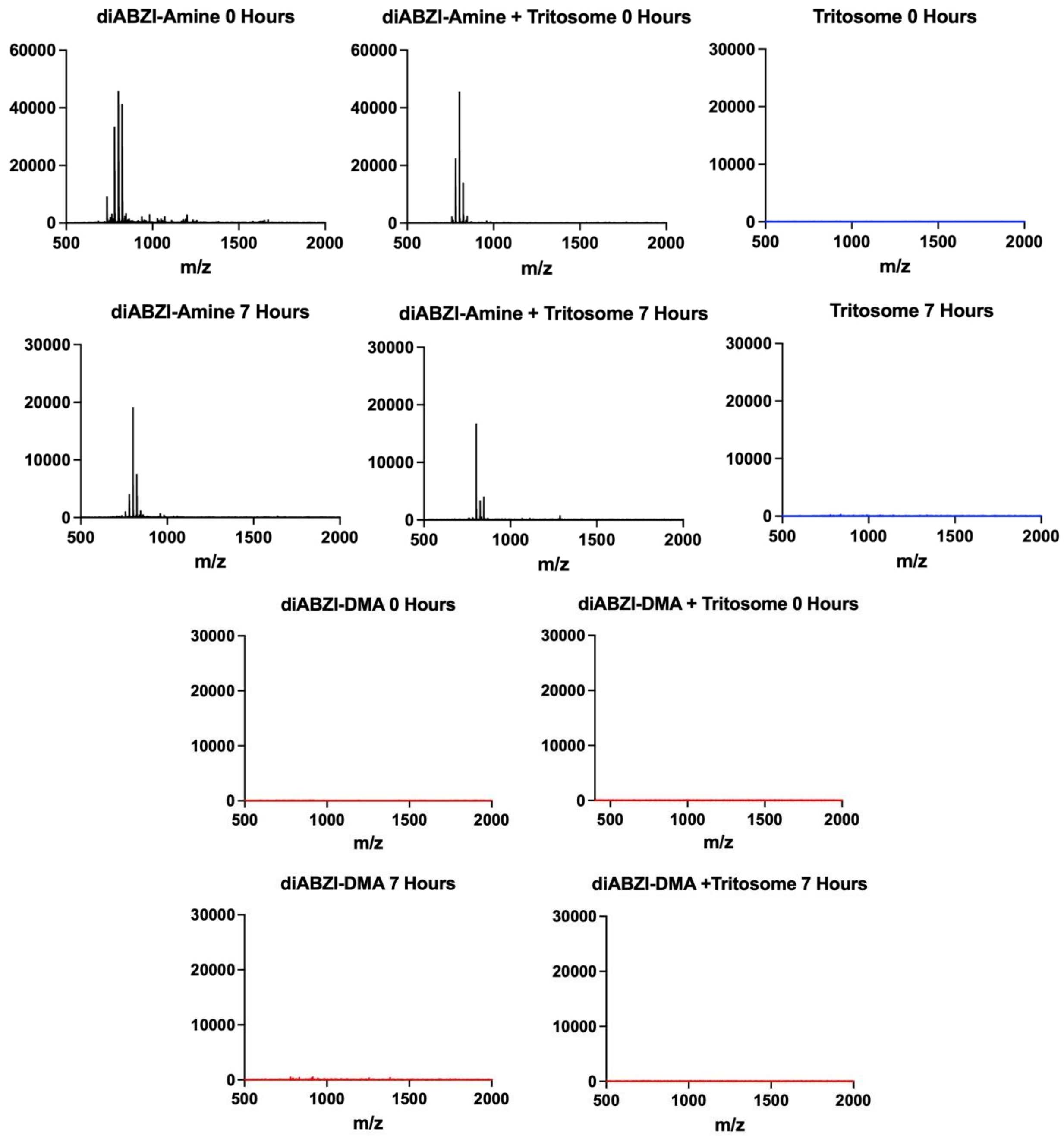

Figure S7: Analysis of diABZI-NH_2 and diABZI-DMA stability in rat liver Tritosomes measured using MALDI-MS. DiABZI-NH_2 or diABZI-DMA_{50kDa} (100 μM) were incubated at 37°C for 7 hours in either water or 10% isolated rat lysosomes (Tritosomes) and MALDI-MS was performed on the samples to detect diABZI-NH_2 or other related variants with m/z between 500-2000. After incubation in Tritosomes for 7h, diABZI-NH_2 remains stable and cannot be detected in diABZI-DMA_{50kDa} sample, indicating lysosomal stability of diABZI-DMA.

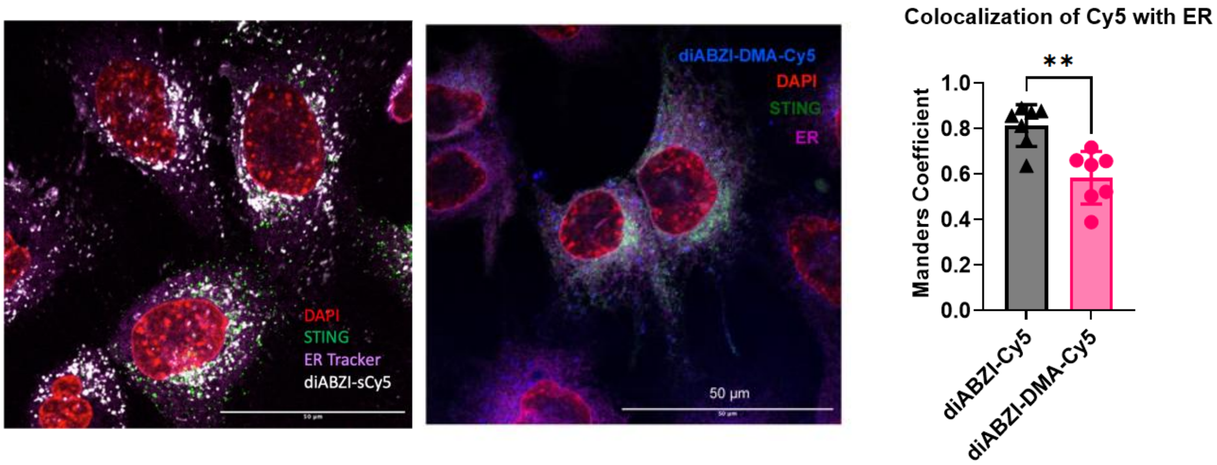

Figure S8: Representative confocal image of STING-GFP MEFs treated with diABZI-sCy5 (left) and diABZI-DMA-sCy5 (middle) for 2 hours and stained for ER Red and DAPI (nuclear stain). Quantification of colocalization between sCy5-labeled diABZI variants and ER Red stain (right) as determined using a Manders coefficient (n = 7) calculated by JACoP ImageJ Plugin.

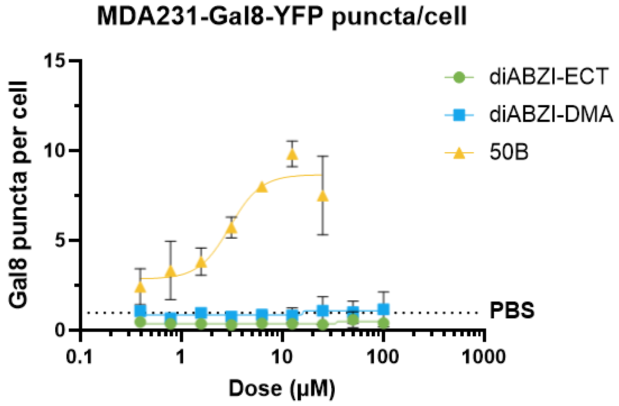

Figure S9: Galectin-8 (Gal8) recruitment assay in MDA231-Gal8-YFP cells used to confirm diABZI-DMA and diABZI-ECT do not have endosomal escape properties, where 50B, a 15 kDa copolymer of 50% DMAEMA and 50% BMA is utilized as an endosomolytic polymer control.

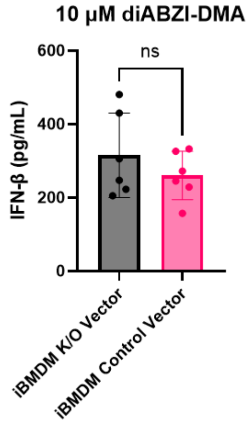

Figure S10: Evaluation of activity of diABZI-DMA in LRRC8A knockout or vector control immortalized bone marrow derived macrophages (iBMDMs) via IFN-b ELISA (n = 6).

Part E: ^{1}H-NMR Spectra for New Compounds

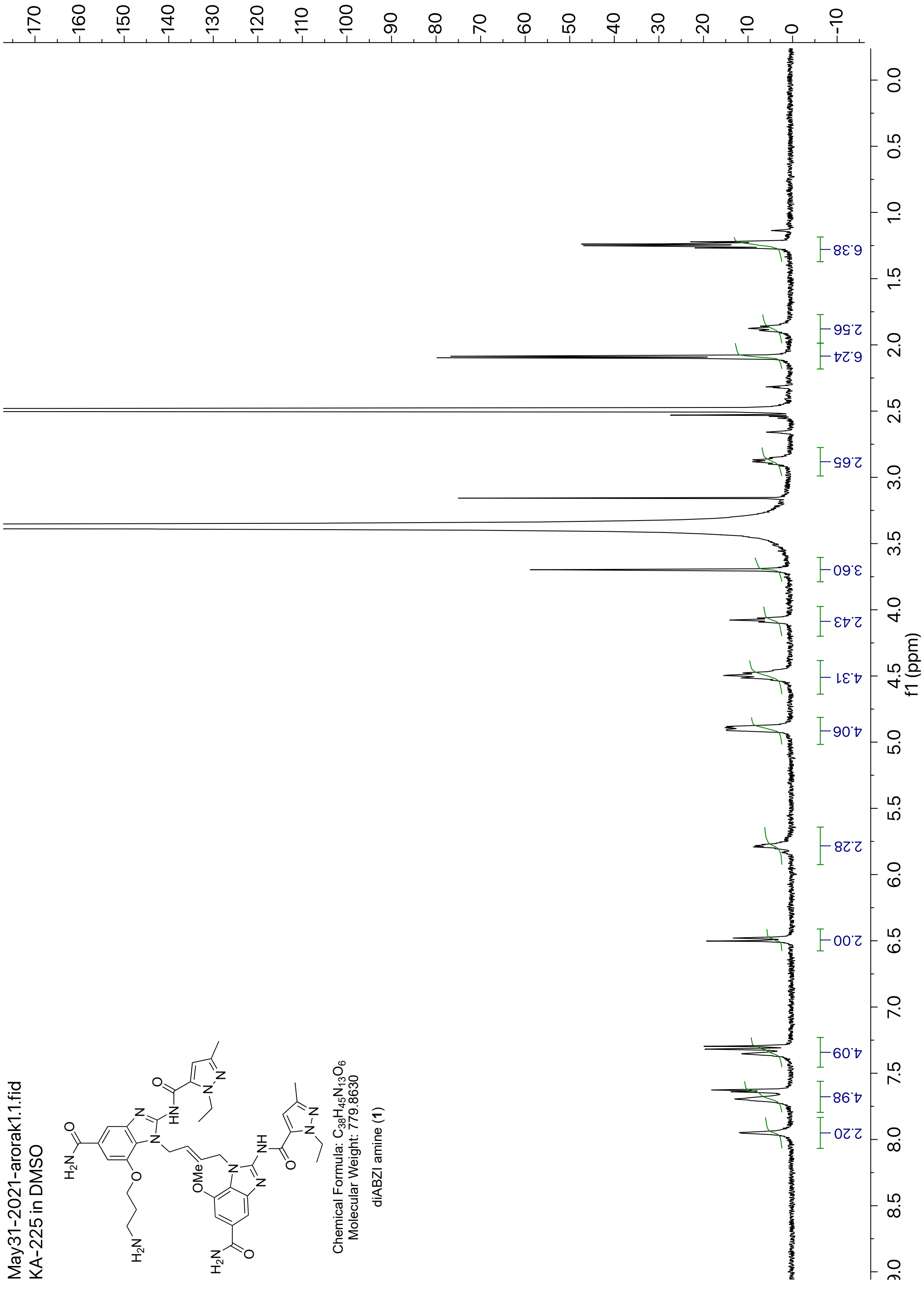

Figure S11: ^{1}H-NMR of diABZI-NH_2 (**1**) in DMSO.

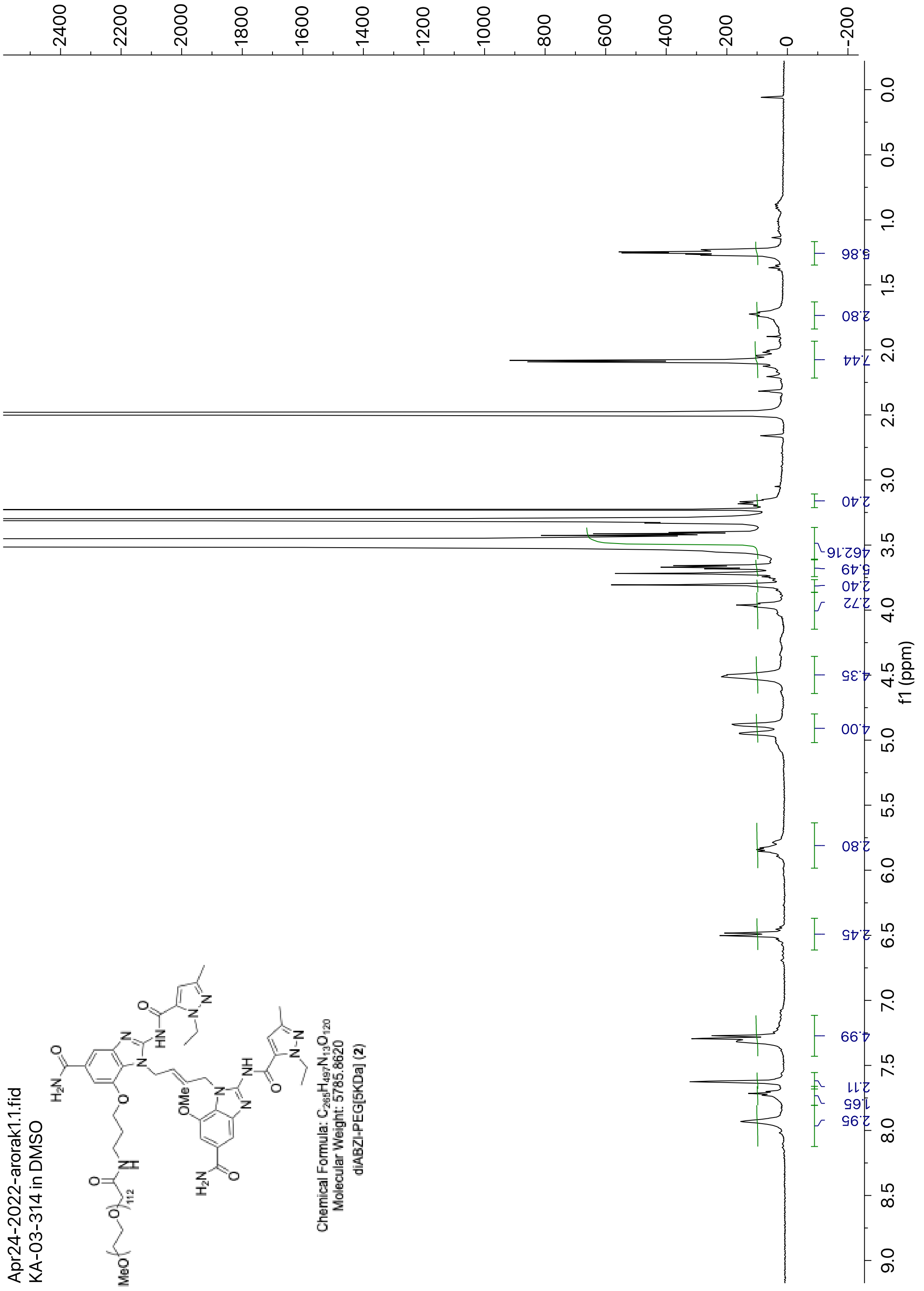

Figure S12: ^{1}H-NMR of diABZI-PEG$_{5kDa}$ (**2**) in DMSO.

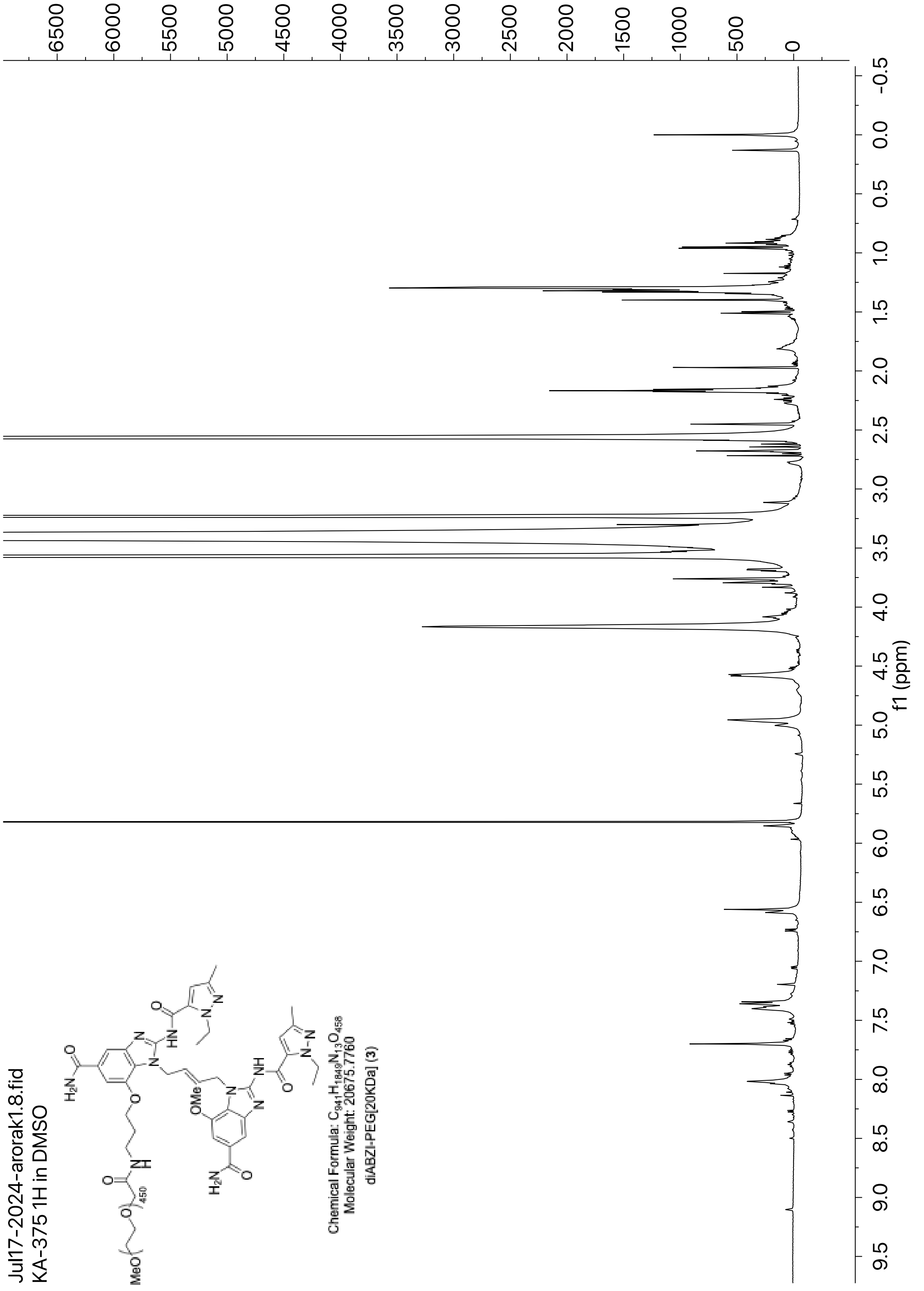

Figure S13: ^{1}H-NMR of diABZI-PEG$_{20kDa}$ (**3**) in DMSO.

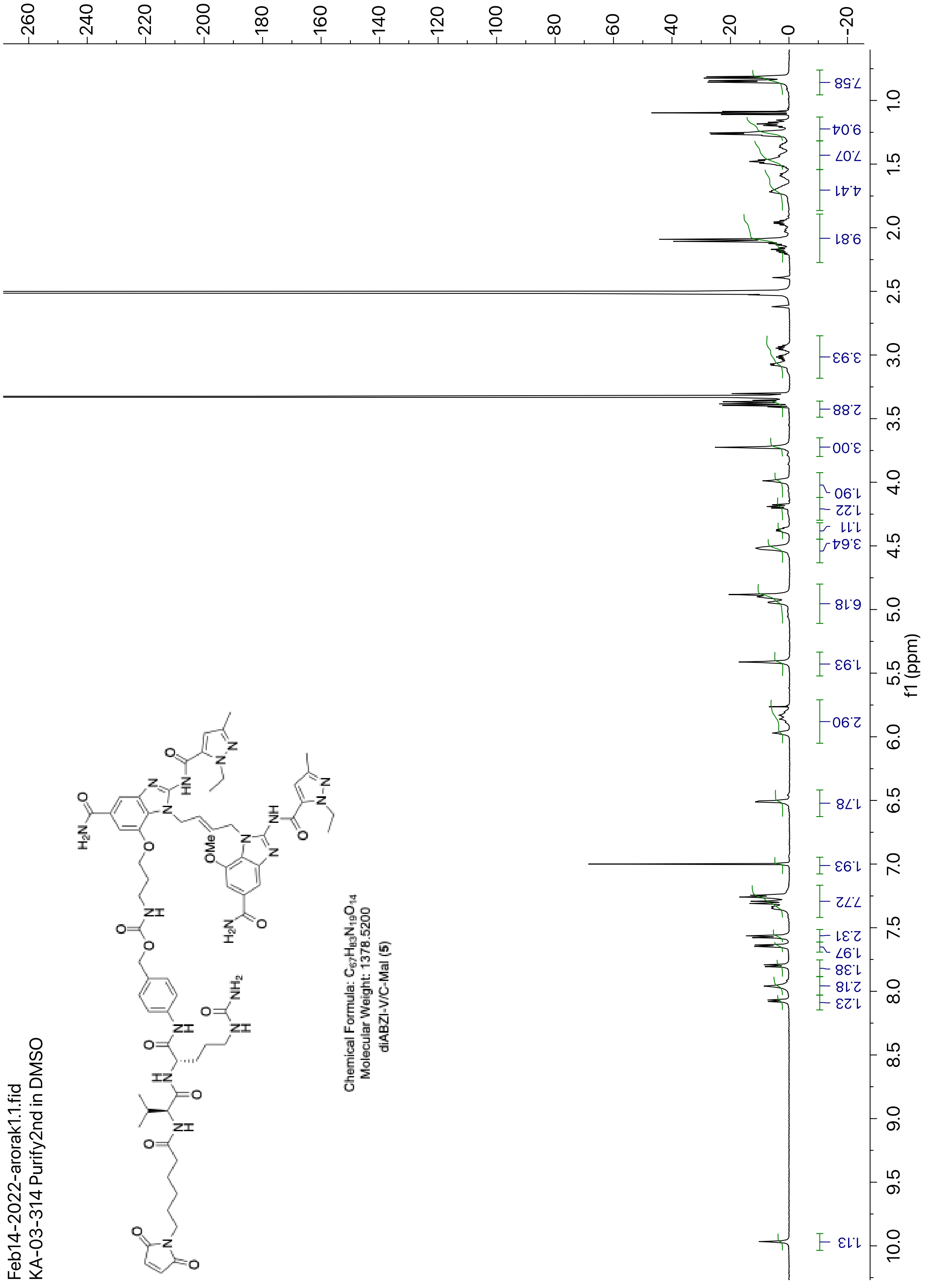

Figure S14: ^{1}H-NMR of diABZI-V/C-Mal (**5**) in DMSO.

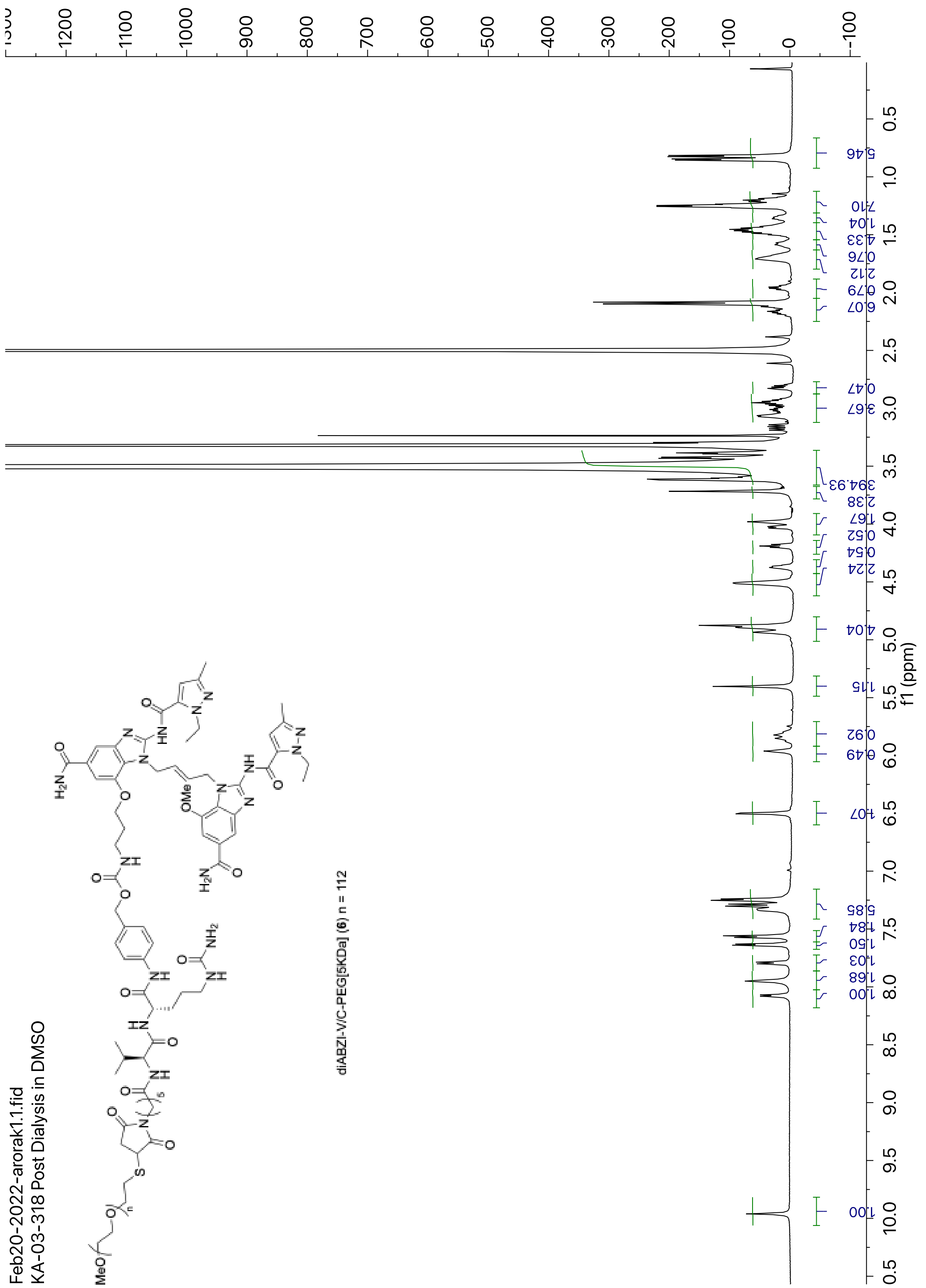

Figure S15: ^{1}H-NMR of diABZI-V/C-PEG$_{5kDa}$ (**6**) in DMSO.

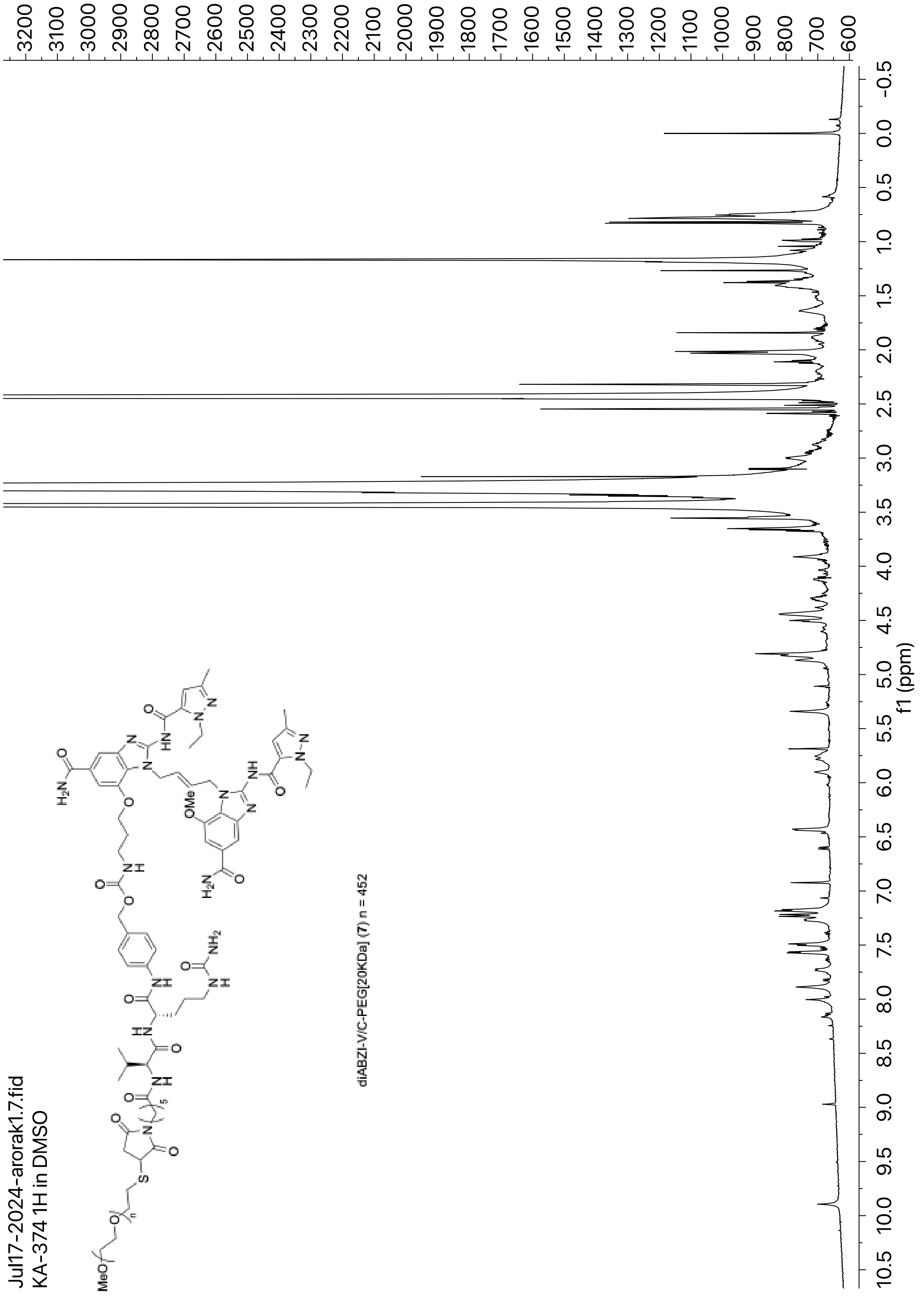

Figure S16: ^{1}H-NMR of diABZI-V/C-PEG$_{20kDa}$ (**7**) in DMSO.

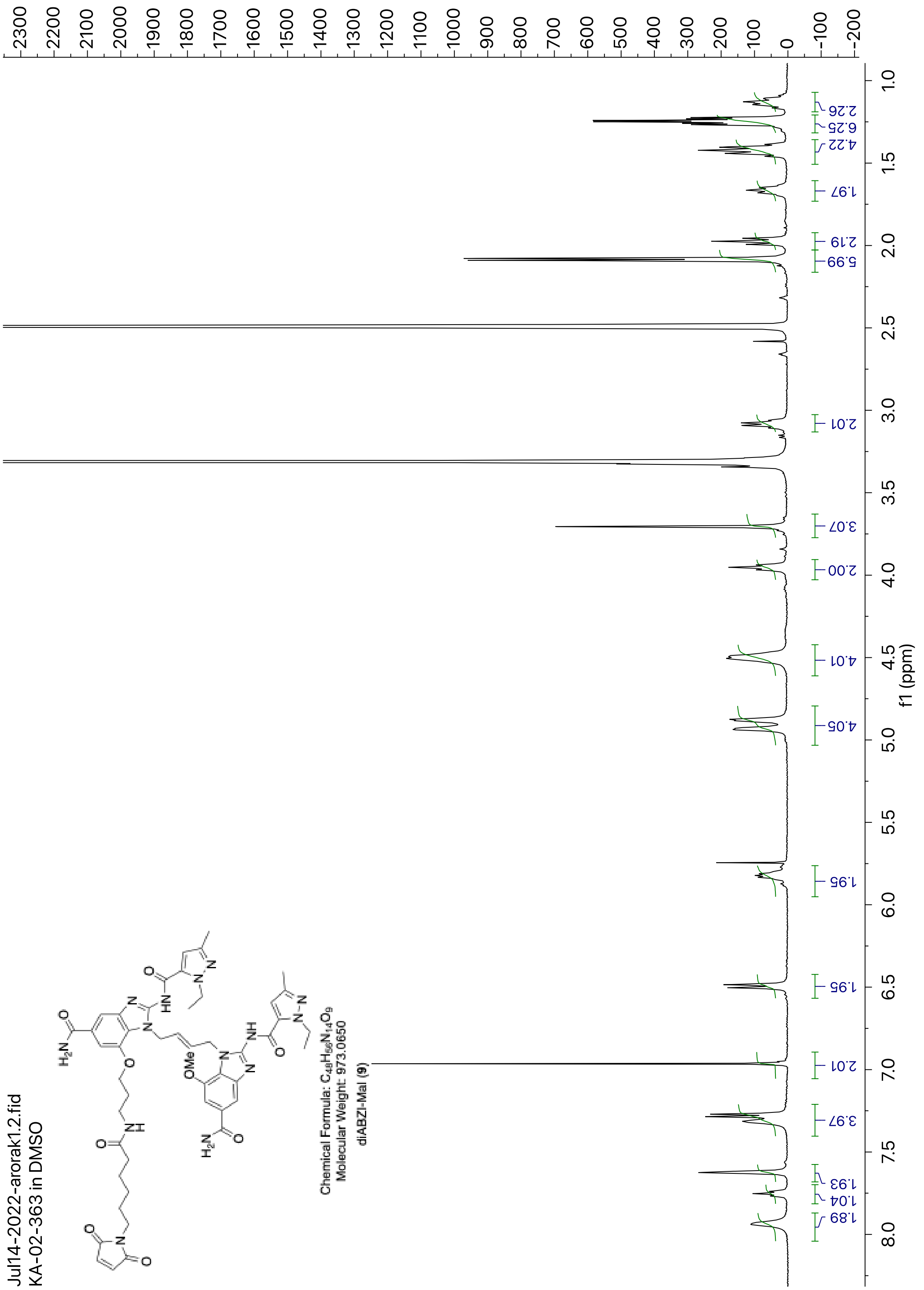

Figure S17: ^{1}H-NMR of diABZI-Mal (**9**) in DMSO.

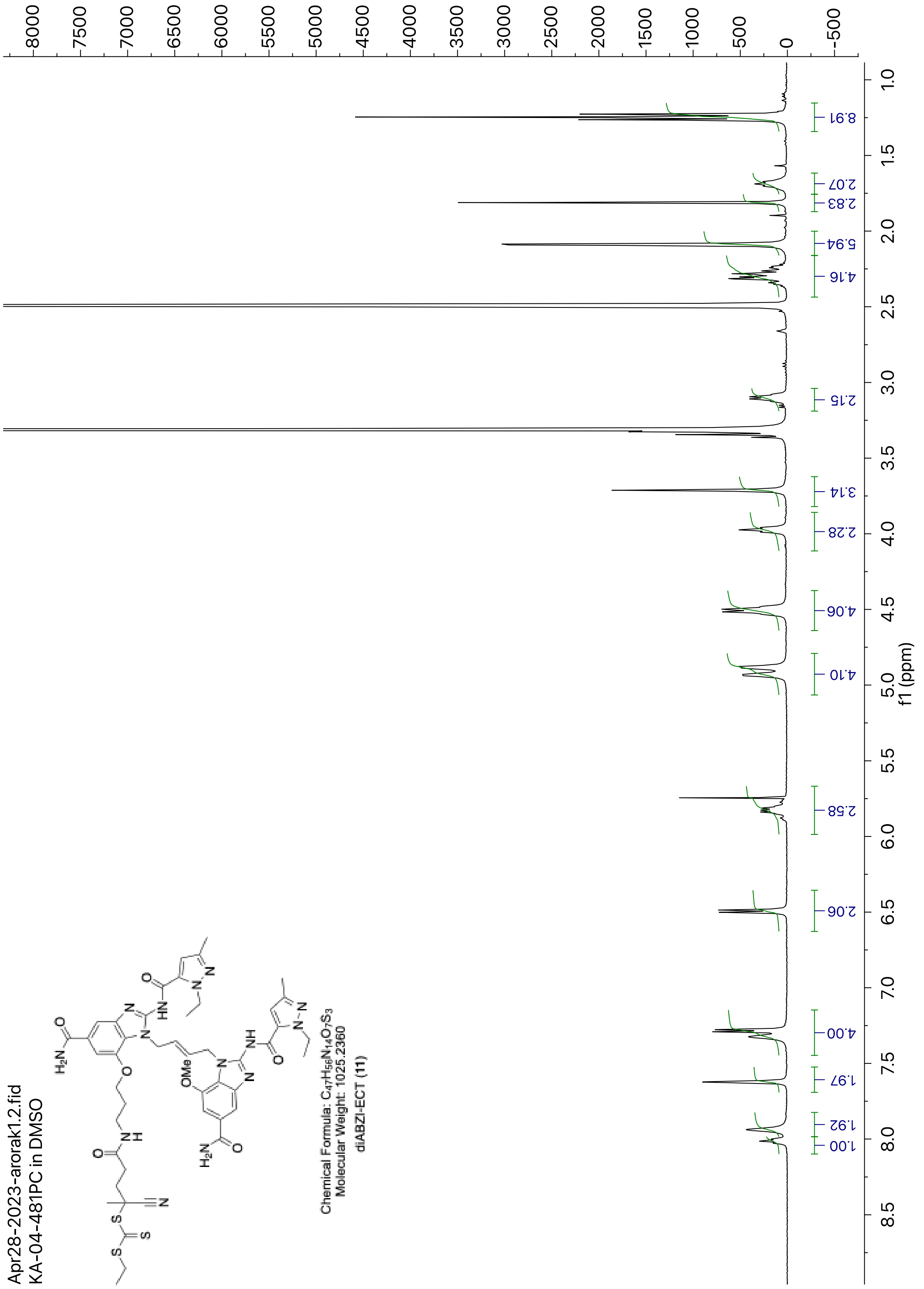

Figure S18: ^{1}H-NMR of diABZI-ECT (**11**) in DMSO.

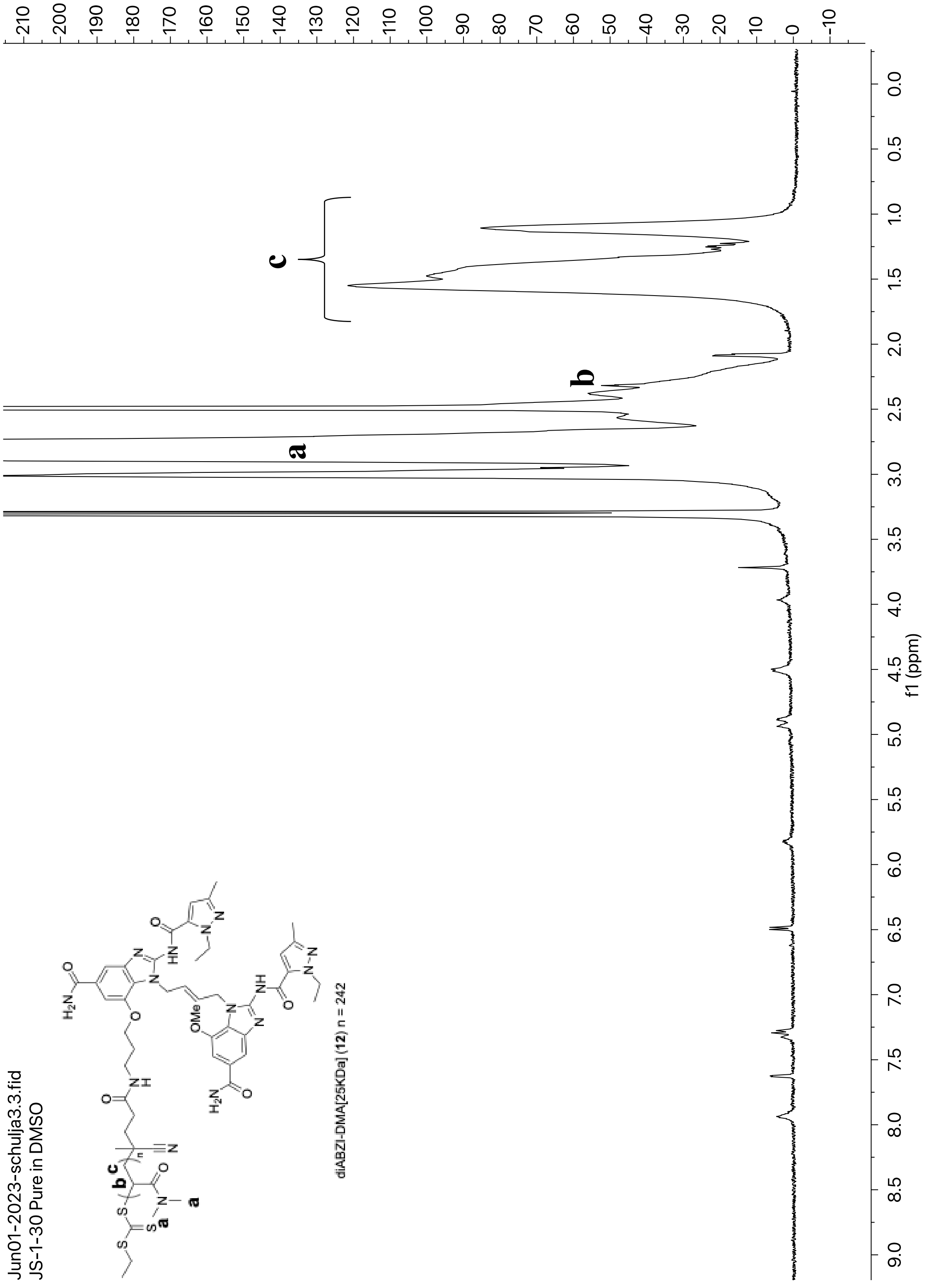

Figure S19: ^{1}H-NMR of diABZI-DMA$_{25kDa}$ (**12**) in DMSO.

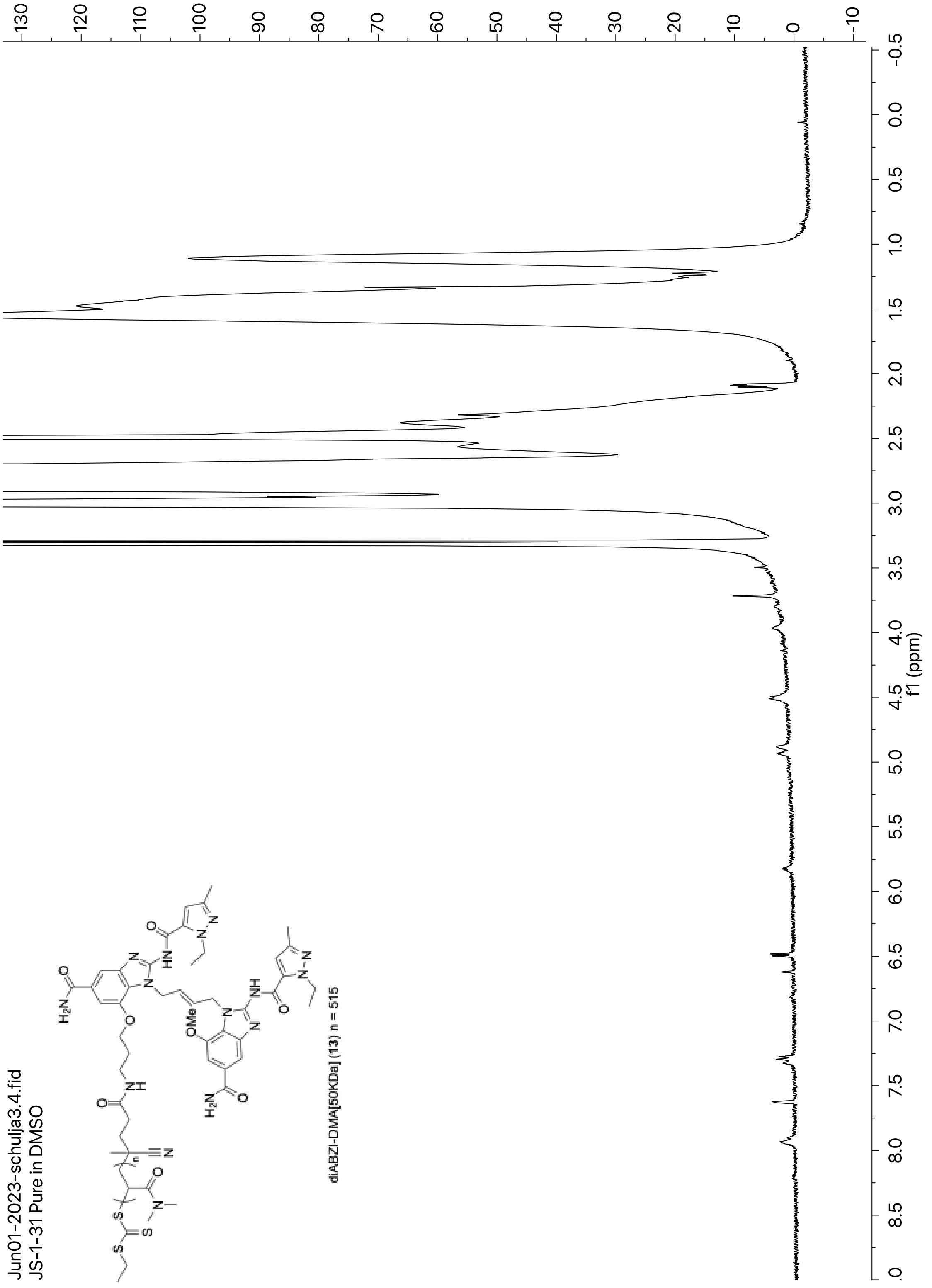

Figure S20: ^{1}H-NMR of diABZI-DMA_{50kDa} (**13**) in DMSO.

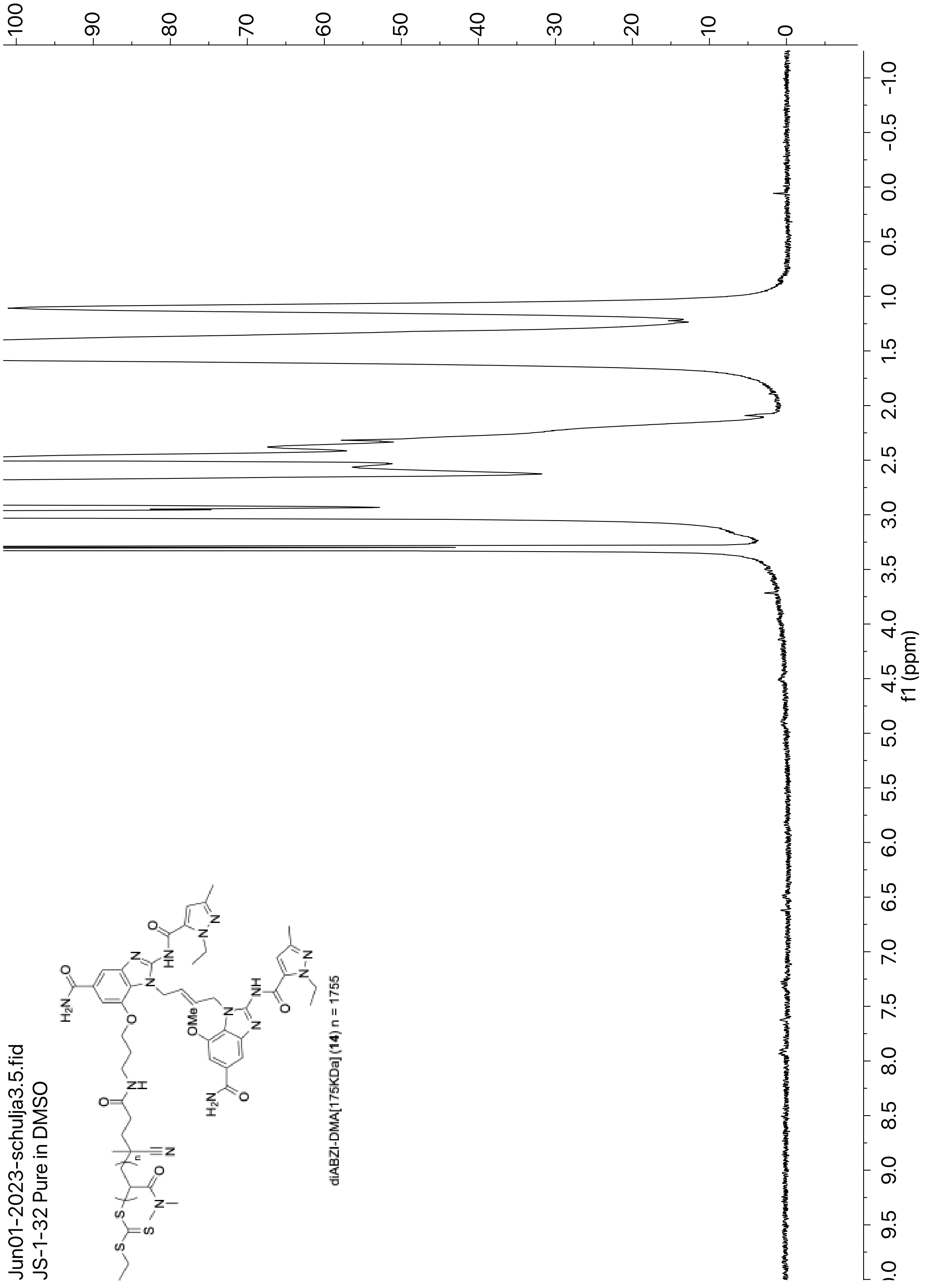

Figure S21: ^{1}H-NMR of diABZI-DMA$_{175kDa}$ (**14**) in DMSO.

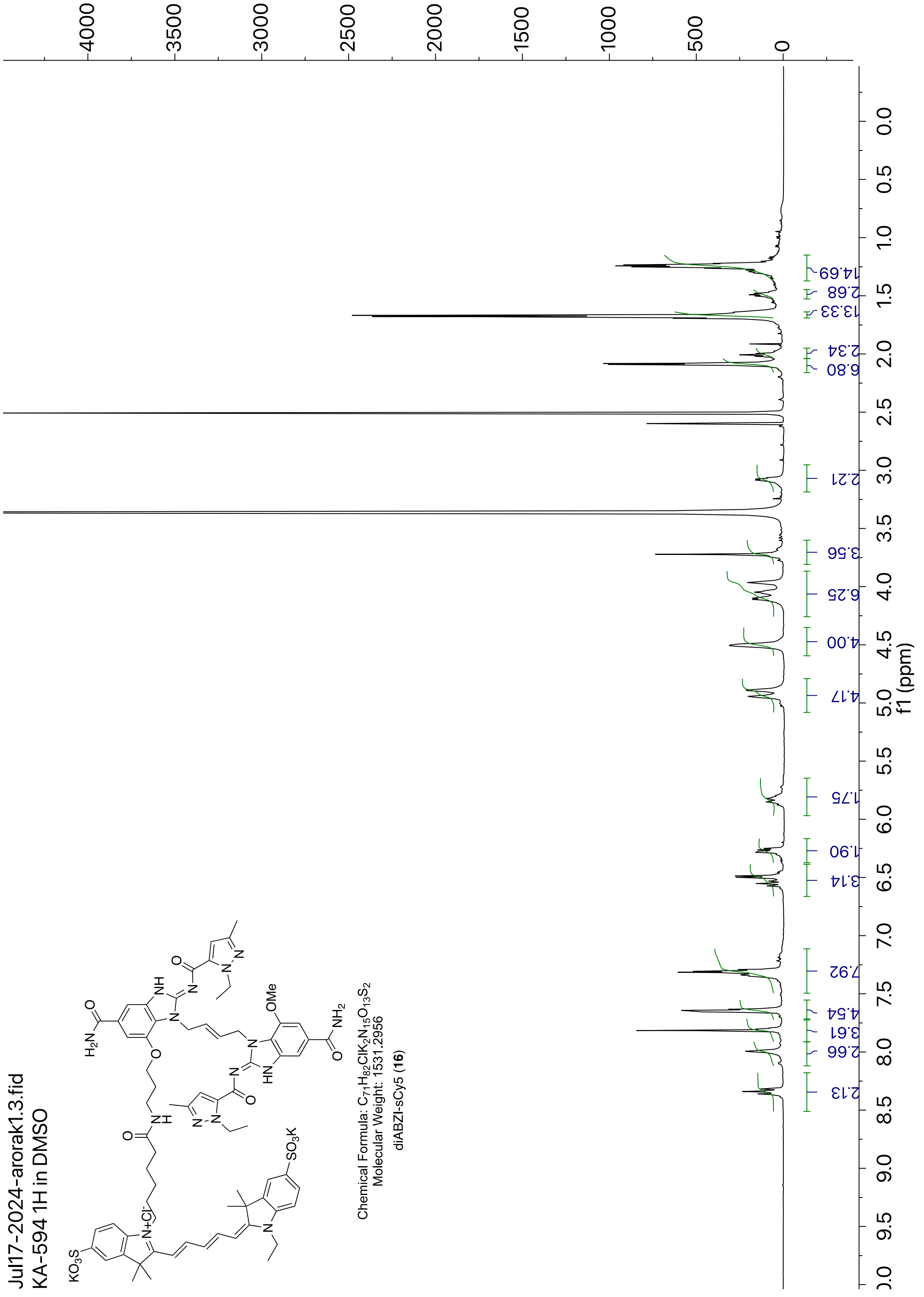

Figure S22: ^{1}H-NMR of diABZI-sCy5 (**16**) in DMSO.

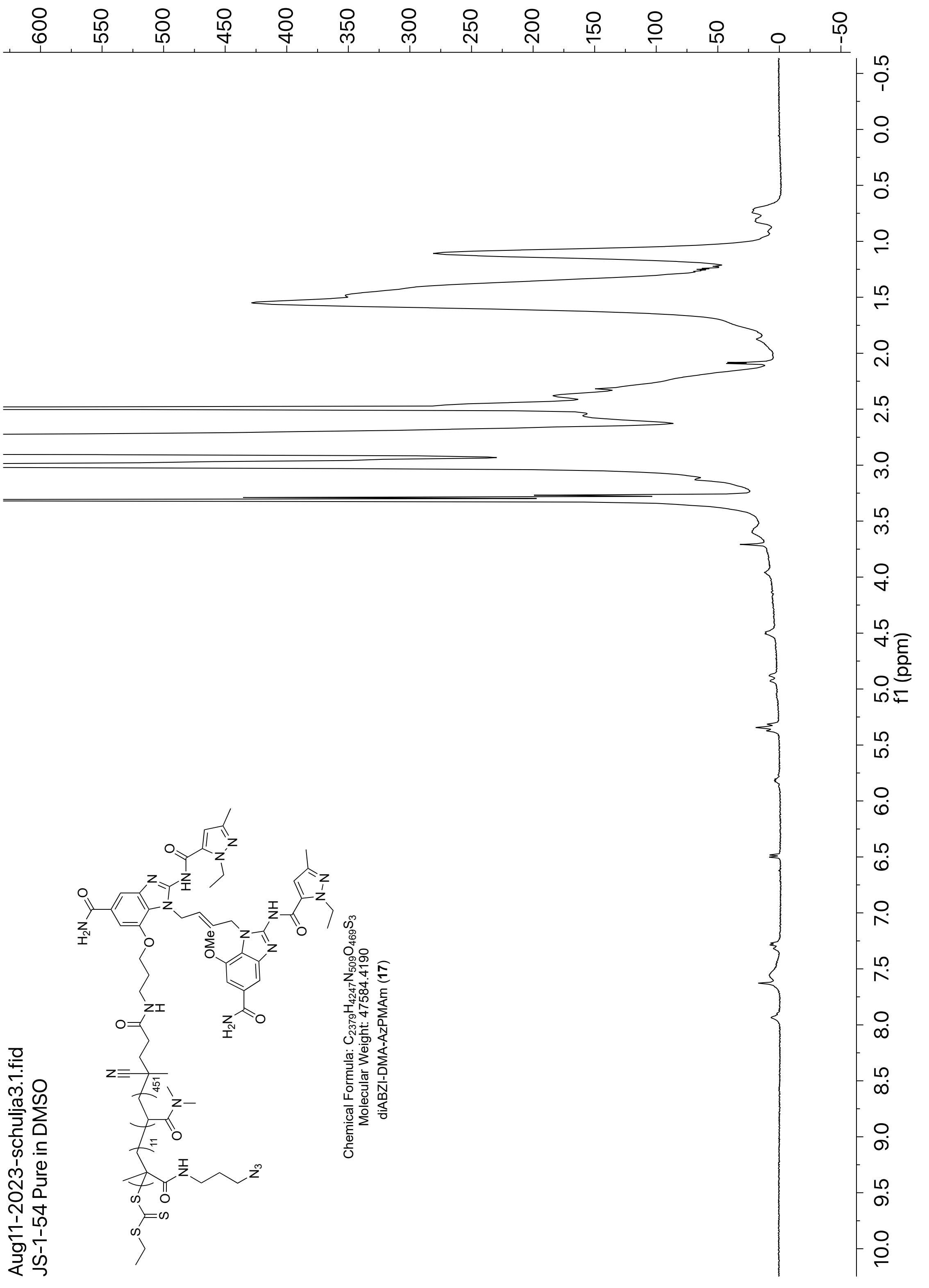

Figure S23: ^{1}H-NMR of diABZI-DMA-co-AzPMAm (**17**) in DMSO.

Part F: ^{13}C-NMR Spectra for New Compounds

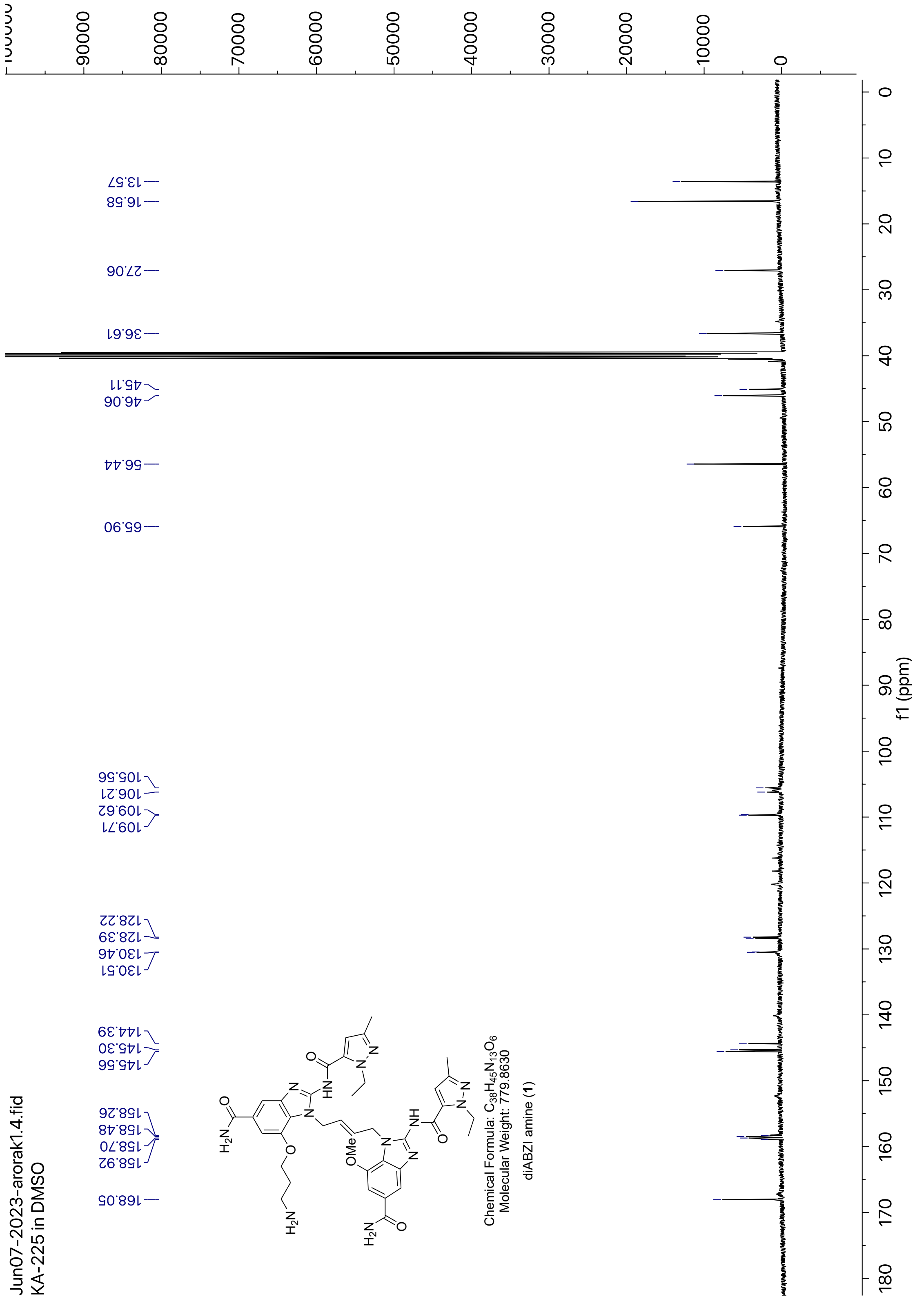

Figure S24: ^{13}C-NMR of diABZI-NH$_2$ (**1**) in DMSO.

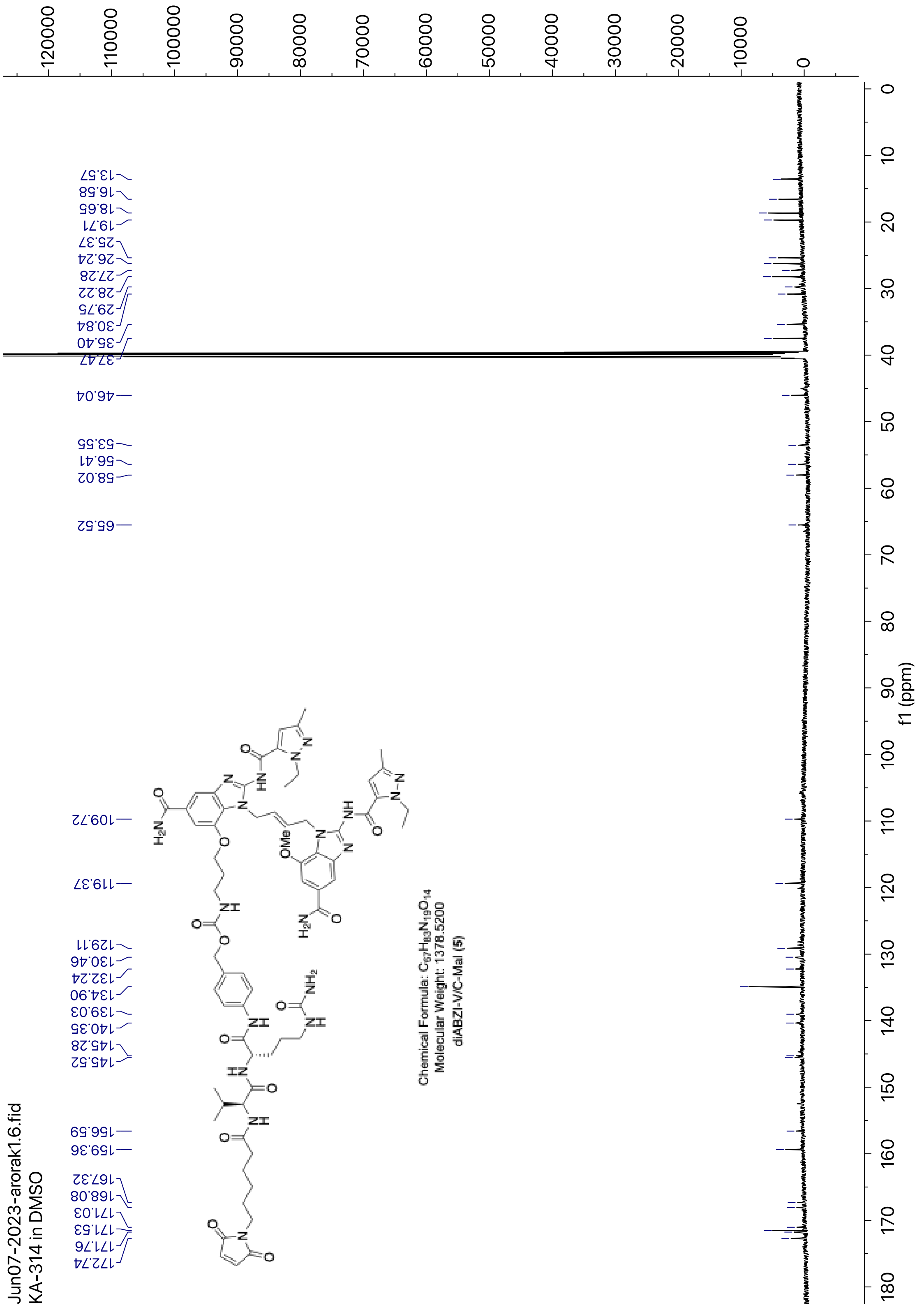

Figure S25: ^{13}C-NMR of diABZI-V/C-Mal (**5**) in DMSO.

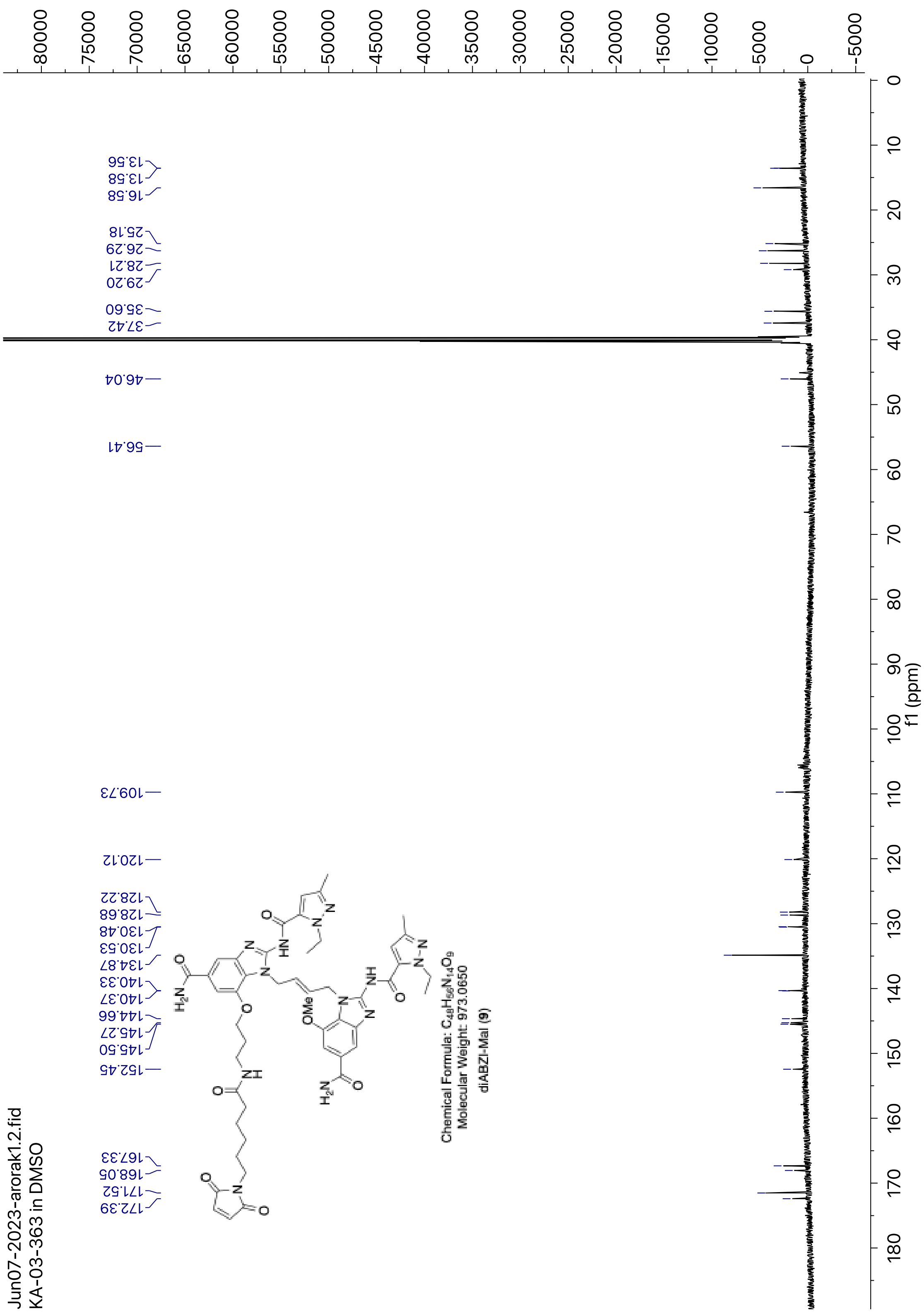

Figure S26: ^{13}C-NMR of diABZI-Mal (**9**) in DMSO.

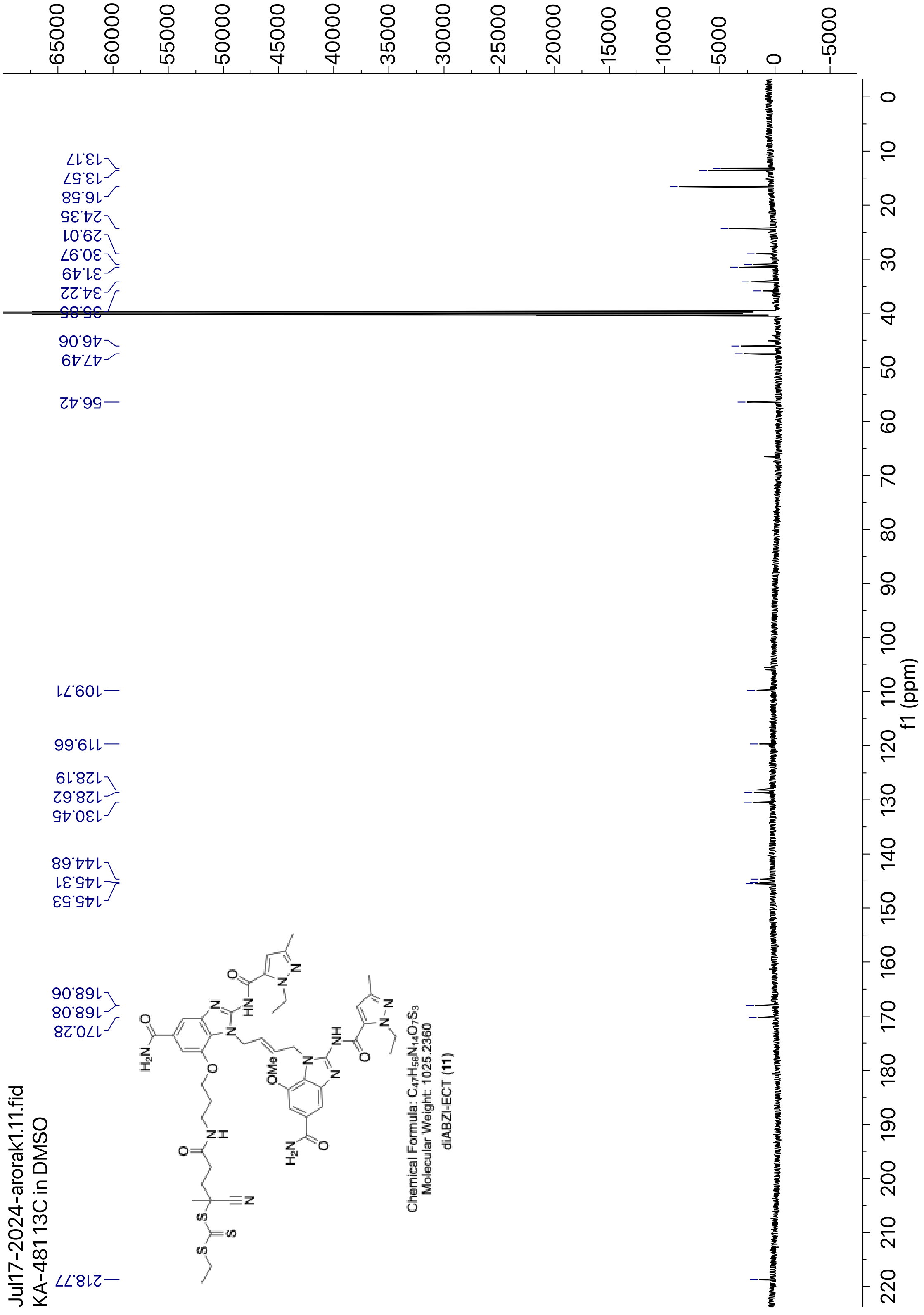

Figure S27: ^{13}C-NMR of diABZI-ECT (**11**) in DMSO.

Part G: HRMS spectra for new compounds

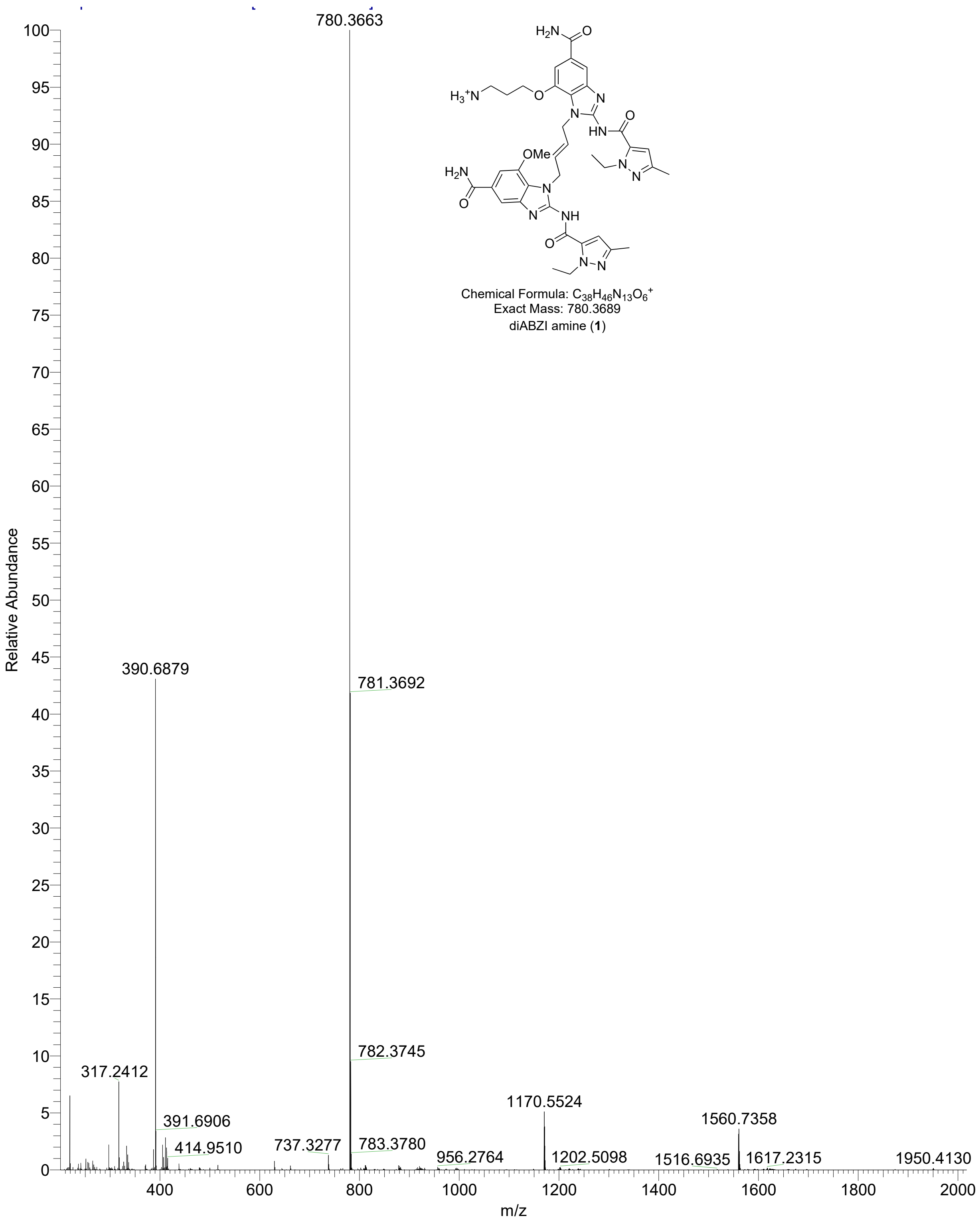

Figure S28: Mass spectrum of diABZI-NH_2 (**1**).

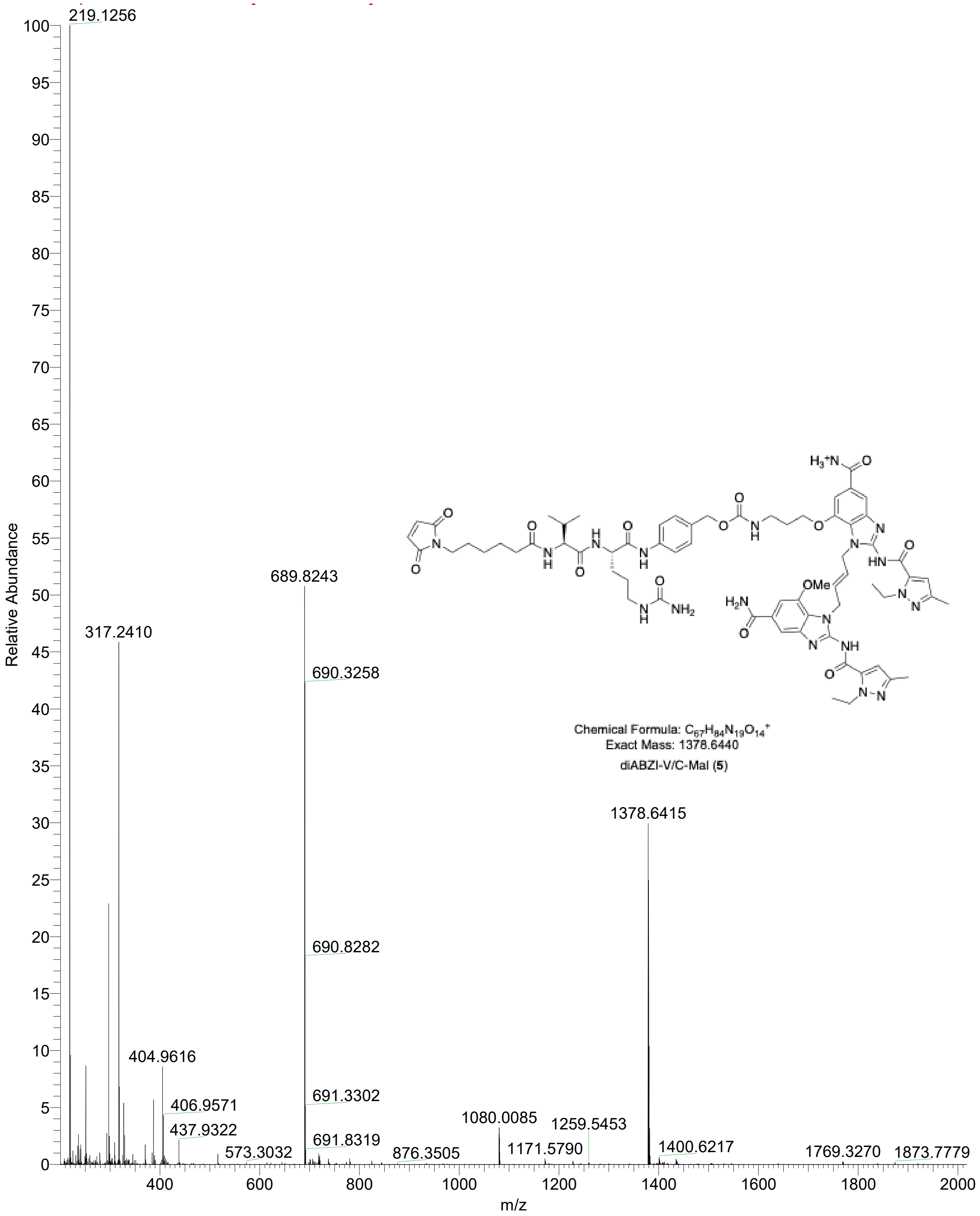

Figure S29: Mass spectrum of diABZI-V/C-Mal (**5**).

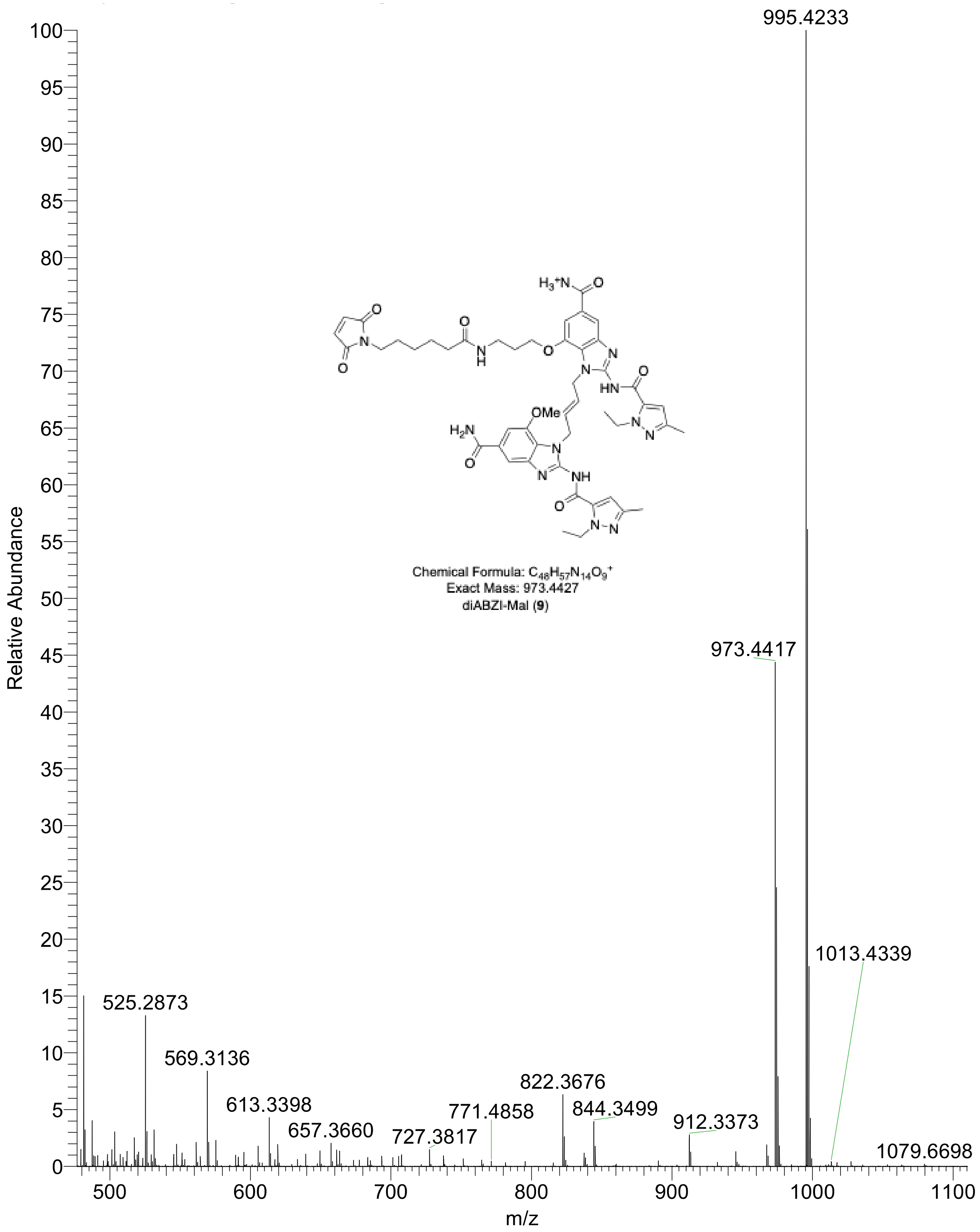

Figure S30: Mass spectrum of diABZI-Mal (**9**).

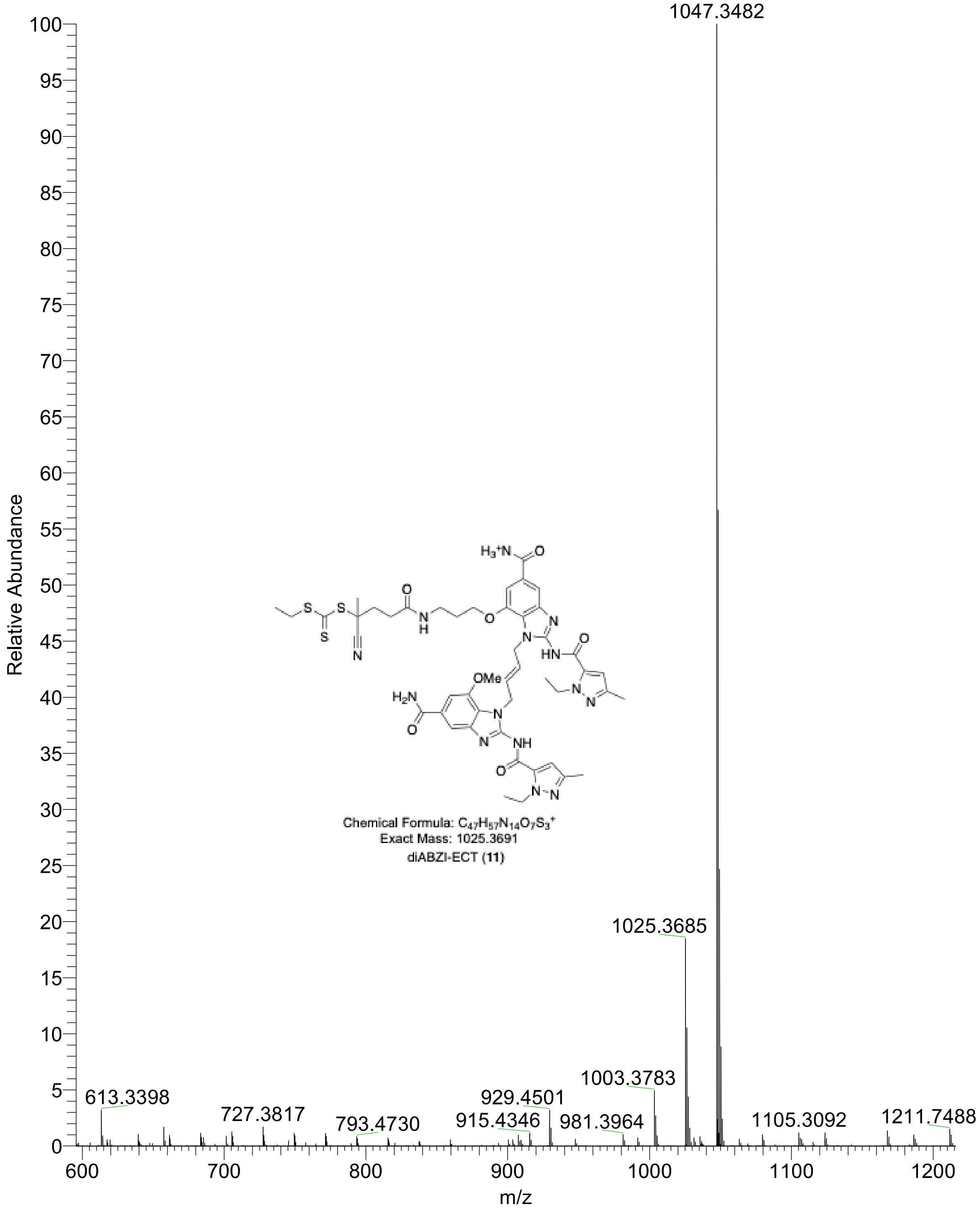

Figure S31: Mass spectrum of diABZI-ECT (**11**).

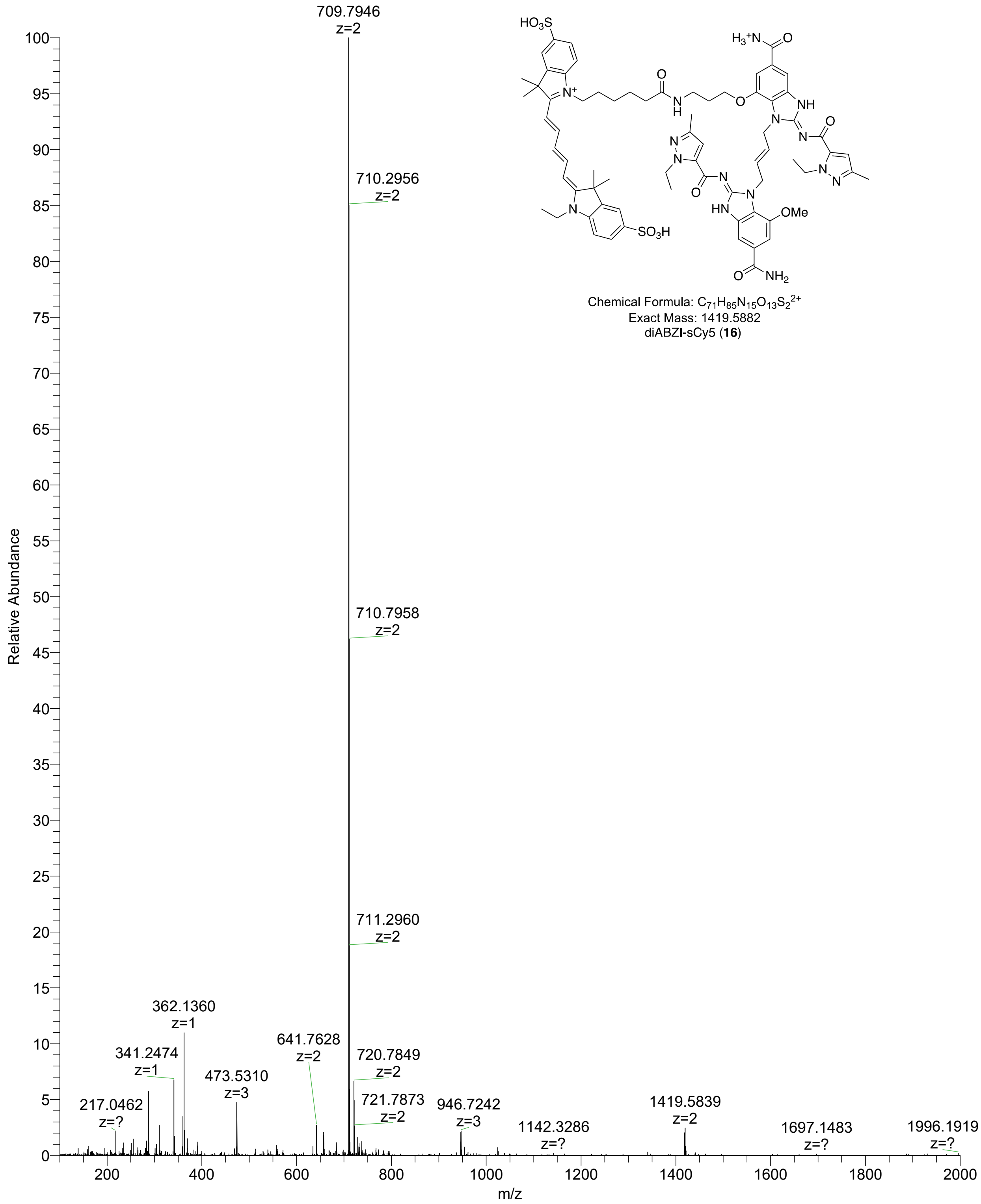

Figure S32: Mass spectrum of diABZI-sCy5 (**16**).

Part H: GPC/LS of Polymers

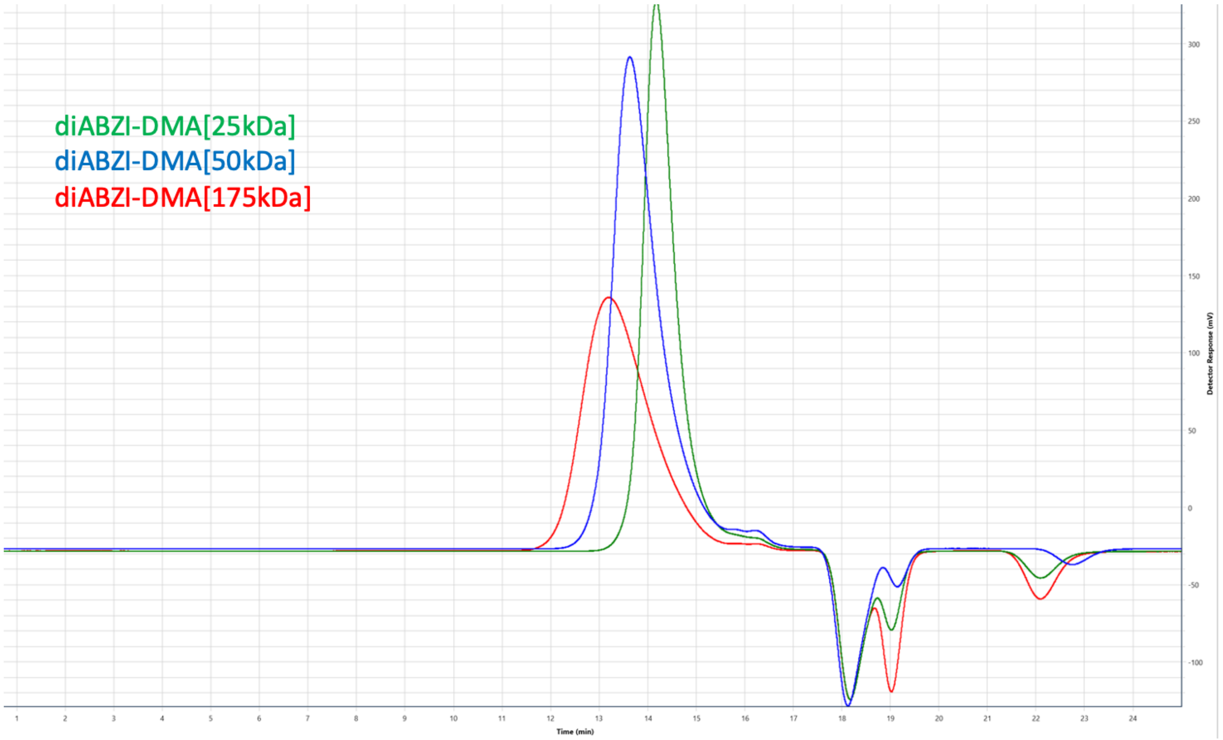

Figure S33: GPC/LS Analysis of diABZI-DMA$_{25kDa}$, diABZI-DMA$_{50kDa}$, diABZI-DMA$_{175kDa}$.

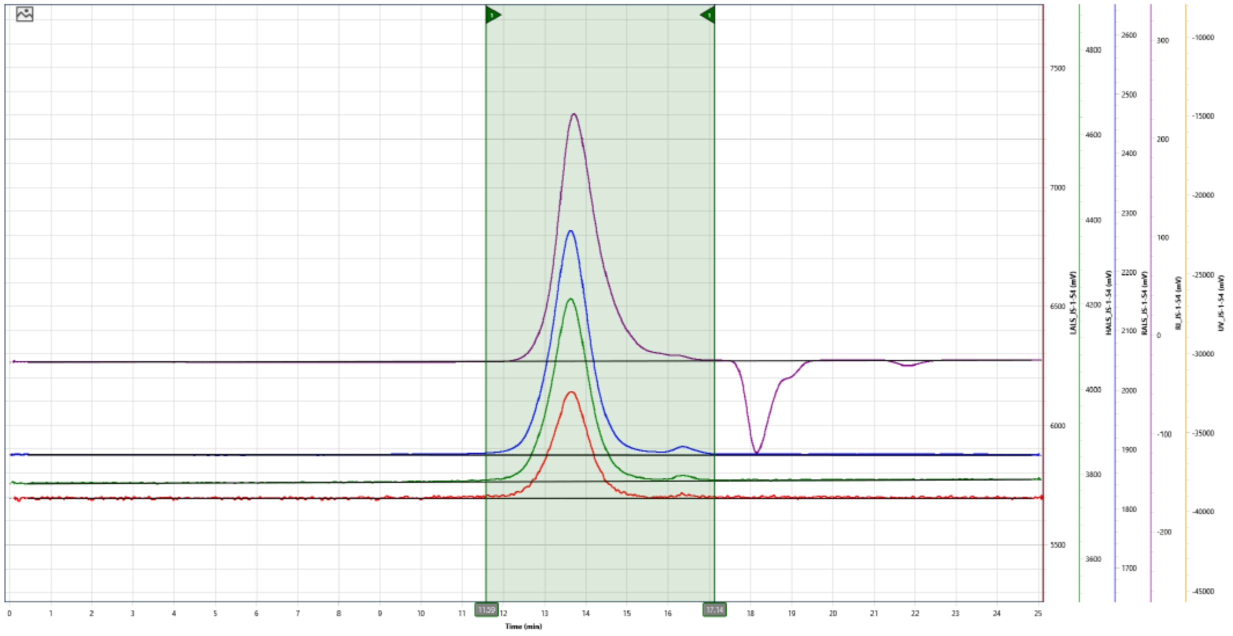

Figure S34: GPC/LS Analysis of diABZI-DMA-co-AzPMAm.

Part I: MS/MS Fragmentation

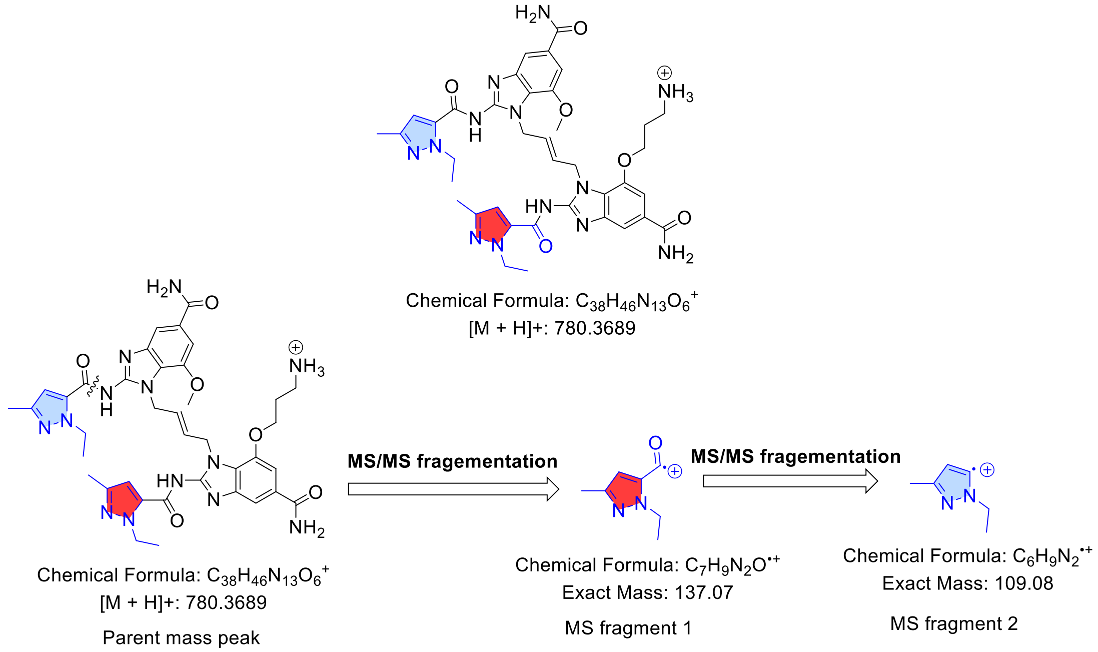

Figure S35. Proposed MS/MS fragmentation pattern of diABZI-amine in positive ESI mode. The protonated molecular ion $[M+H]^+$ was detected at m/z 780.400. Fragmentation of the precursor ion at m/z 109, corresponding to the loss of a characteristic fragment. Additional product ions were observed at m/z 137 and 364 (not shown). The proposed fragmentation pathway and product ions are illustrated based on the observed spectra.

Part J: Microscopy Videos

See uploaded .avi files

Videos 1 and 2: Time-lapse from MEF cells expressing a STING-GFP fusion protein treated with diABZI-ECT or diABZI-DMA for the indicated time. The formation of GFP puncta corresponds to multimerization of STING following ligand binding.

Part K: References

(1) Sheehy, T.L.; Kwiatkowski, A.J.; Arora, K.; Kimmel, B. R.; Schulman, J.A.; Gibson-Corley, K. N.; Wilson, J.T.; STING-Activating Polymer-Drug Conjugates for Cancer Immunotherapy. *bioRxiv* **2024**, 2024.2003.2023.585817. DOI: 10.1101/2024.03.23.585817.

(2) Blanchard, S.; Coats, J. Process for the preparation of peptide drug linker compounds. WO2019108797, 2019.

(3) van Dongen, S. F. M.; Clerx, J.; Norgaard, K.; Bloemberg, T. G.; Cornelissen, J. J. L. M.; Trakselis, M. A.; Nelson, S. W.; Benkovic, S. J.; Rowan, A. E.; Nolte, R. J. M. A clamp-like biohybrid catalyst for DNA oxidation. *Nat. Chem.* **2013**, *5* (11), 945-951. DOI: 10.1038/nchem.1752.

(4) Qiu, J.; Meng, F.; Wang, M.; Huang, J.; Wang, C.; Li, X.; Yang, G.; Hua, Z.; Chen, T. Recyclable DMAP-Functionalized polymeric nanoreactors for highly efficient acylation of alcohols in aqueous systems. *Polymer* **2021**, *222*, 123660. DOI: 10.1016/j.polymer.2021.123660.

(5) Goor, O. J. G. M.; Keizer, H. M.; Bruinen, A. L.; Schmitz, M. G. J.; Versteegen, R. M.; Janssen, H. M.; Heeren, R. M. A.; Dankers, P. Y. W. Efficient Functionalization of Additives at Supramolecular Material Surfaces. *Adv. Mater. (Weinheim, Ger.)* **2017**, *29* (5), n/a. DOI: 10.1002/adma.201604652. Luzuriaga, M. A.; Welch, R. P.; Dharmarwardana, M.; Benjamin, C. E.; Li, S.; Shahrivarkevishahi, A.; Popal, S.; Tuong, L. H.; Creswell, C. T.; Gassensmith, J. J. Enhanced Stability and Controlled Delivery of MOF-Encapsulated Vaccines and Their Immunogenic Response In Vivo. *ACS Appl. Mater. Interfaces* **2019**, *11* (10), 9740-9746. DOI: 10.1021/acsami.8b20504.
